# Tumor-Targeted Delivery of an EGFR Inhibitor Prodrug via Site-Specific Albumin Conjugation

**DOI:** 10.1021/acs.jmedchem.5c02536

**Published:** 2026-01-23

**Authors:** Anja Federa, Rastislav Pitek, Orsolya Dömötör, Éva A. Enyedy, Alessio Terenzi, Monika Caban, Alessia Stefanelli, Luisa D’Anna, Faye White, Petra Heffeter, Christian R. Kowol

**Affiliations:** † Institute of Inorganic Chemistry, Faculty of Chemistry, 27271 University of Vienna, Waehringer Str. 42, A-1090 Vienna, Austria; ‡ Vienna Doctoral School in Chemistry, University of Vienna, Waehringer Str. 42, 1090 Vienna, Austria; § Center for Cancer Research and Comprehensive Cancer Center, Medical University of Vienna, Borschkegasse 8a, A-1090 Vienna, Austria; ∥ Department of Molecular and Analytical Chemistry, 27258 University of Szeged, Dóm tér 7-8, H-6720 Szeged, Hungary; ⊥ Department of Biological, Chemical and Pharmaceutical Sciences and Technologies, 18998 University of Palermo, Viale delle Scienze, Edificio 17, 90128 Palermo, Italy; # Research Cluster “Translational Cancer Therapy Research”, 1090 Vienna, Austria

## Abstract

Albumin is a promising vehicle for anticancer drug delivery due to its high plasma concentration, long half-life and known tumor accumulation. Drugs can be covalently conjugated to albumin via the free thiol at Cys^34^, using maleimide chemistry. Interestingly, such strategies have not yet been applied to tyrosine kinase inhibitors (TKIs), e.g. crucial in lung cancer treatment. This study investigates a prodrug delivery system for a derivative of the approved epidermal growth factor receptor (EGFR) inhibitor osimertinib, incorporating a maleimide for albumin binding and a cathepsin B-cleavable valine-citrulline (ValCit) dipeptide for selective drug release. *In silico* and *in vitro* studies confirmed the prodrug nature. Additionally, selective albumin-binding and efficient cathepsin B-mediated drug release were demonstrated. In non-small cell lung cancer (NSCLC) xenografts, the prodrug exhibited enhanced anticancer activity compared to osimertinib and a noncleavable glycine-glycine (GlyGly) control. These results highlight covalent albumin-binding as a promising strategy for TKI delivery.

## Graphical abstract

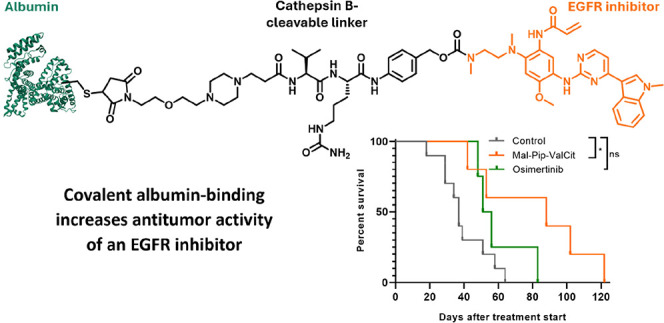

## Introduction

A major challenge in anticancer therapy is the limited tumor specificity of many drugs, which often leads to severe adverse effects.^1^ Human serum albumin (HSA) has long been identified as a very promising tool for improved drug delivery and tuning of pharmacokinetic properties. With a half-life of ∼19 days, a plasma concentration of 35–50 g/L (∼640 μM), and several hydrophobic binding pockets, HSA serves as a natural transporter of diverse endogenous ligands and drugs, significantly prolonging their circulation.^2–4^ The remarkably long half-life of HSA results from binding to the neonatal fragment crystallizable (Fc) receptor after endocytosis, effectively protecting it from lysosomal degradation.^5^ Moreover, albumin accumulates in solid tumors through both active and passive targeting mechanisms. First, it is actively taken up by cancer cells via endocytosis, serving as a nutrient source.^6^ Second, albumin passively targets tumors through the enhanced permeability and retention (EPR) effect, entering malignant tissue through leaky vasculature and accumulating due to impaired lymphatic drainage.^7–9^ Emphasizing this, our recent study demonstrated albumin accumulation in tumors using a radioactive [^89^Zr]­Zr-DFO* complex covalently conjugated to HSA via a maleimide linker.^10^ The use of a rather long-lived radioisotope in form of a highly stable HSA-conjugate yielded exceptionally high tumor-to-tissue ratios, visualizing the remarkable efficiency of albumin-mediated tumor targeting. Some oncological drugs already make use of albumin for drug delivery, including US Food and Drug Administration (FDA)-approved Abraxane, a nanoparticle albumin-bound formulation of paclitaxel.^3,11^ Another example is the experimental drug Aldoxorubicin, a maleimide-functionalized derivative of doxorubicin that has advanced to phase 3 clinical trials (ClinicalTrials.gov number, NCT02049905). Following intravenous administration, it selectively forms a covalent bond with the Cys^34^ residue of endogenous albumin.^3,12,13^

Tyrosine kinase inhibitors (TKIs) are targeted therapeutics and represent one of the most prolific areas in the development of novel anticancer drugs. TKIs bind to the adenosine triphosphate (ATP)-binding pocket of receptor tyrosine kinases (RTKs), disrupting intracellular downstream signaling and inducing apoptosis.^14,15^ As of 2025, the FDA has approved 75 protein kinase inhibitors to treat neoplasms, including 45 receptor TKIs.^16^ As TKIs are directed toward molecular targets involved in cancer cell proliferation, an increased selectivity for cancerous over healthy cells is frequently observed.^17,18^ However, as RTK-induced signaling is upregulated but not confined to malignant cells,^19^ severe adverse effects remain an issue. Some of the most common ones are cardiovascular events (e.g., hypertension, heart failure, etc.), gastrointestinal symptoms (e.g., nausea, diarrhea, etc.) and skin rash.^20,21^

Many approved TKIs are well-known to electrostatically bind to albumin, often reaching >90% protein binding in serum.^22–25^ Yet, to the best of our knowledge, there is no direct evidence that this interaction contributes to tumor targeting. In contrast, several studies even suggest that due to this extensive protein binding, the low levels of unbound drug in serum can limit their clinical activity.^26–28^ This widely accepted principle, known as the Free Drug Hypothesis, states that only the unbound or free fraction of a drug is responsible for the activity at the therapeutic target site.^29,30^ Of note, also TKI toxicity is influenced by their interaction with albumin. Numerous studies demonstrated that patients with hypoalbuminemia show significantly more adverse events, leading to early treatment discontinuation.^31^ This implies that elevated levels of free drug can cause severe toxicity and that albumin binding acts like a protective reservoir at the expense of decreased drug activity.

Therefore, we envisioned that the activity of TKIs could be improved if the drug is bound to albumin covalently via a maleimide linker and can be selectively released upon activation in the tumor tissue (Figure 1).

**Figure 1:**
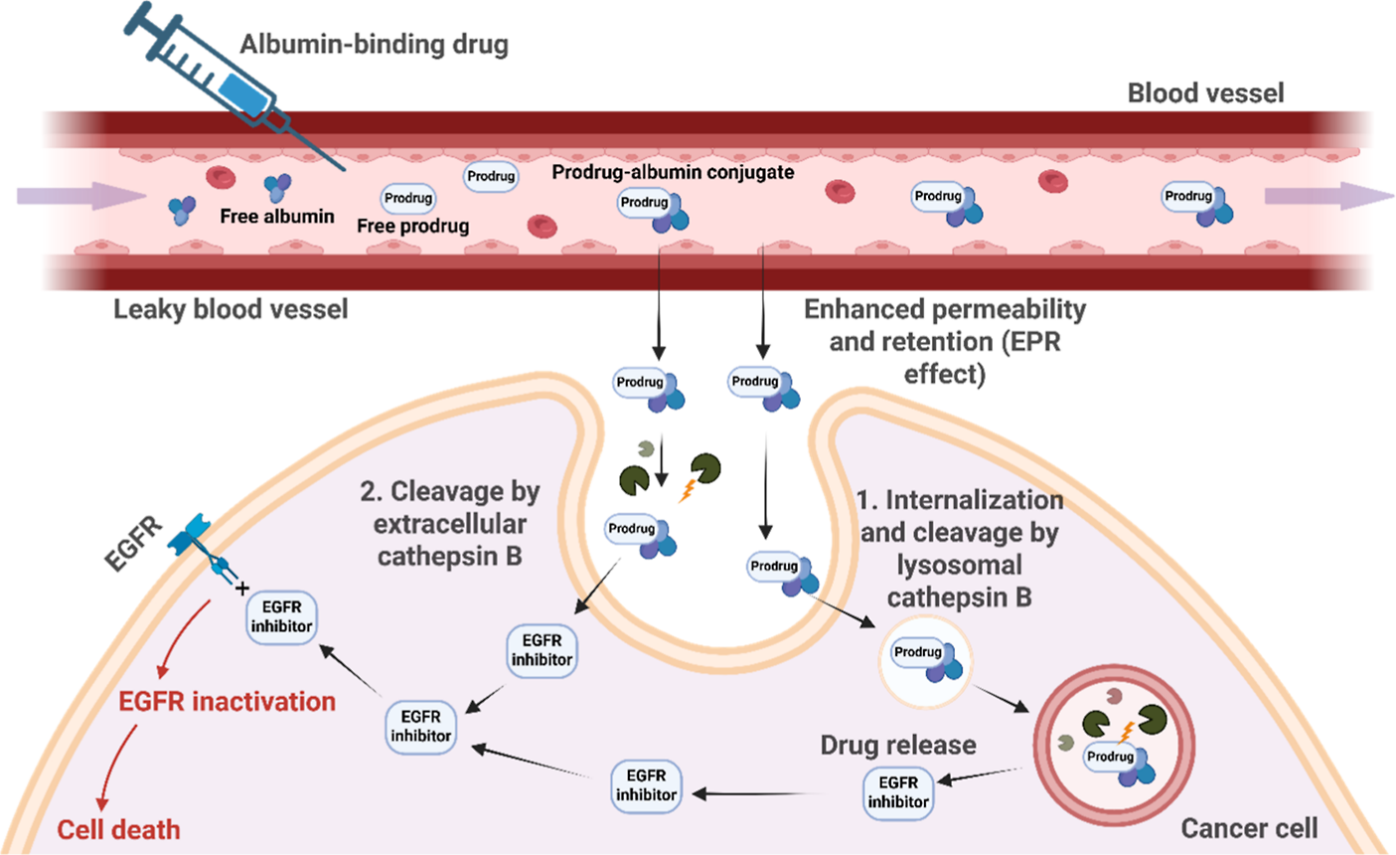
Mechanism of action of the albumin-binding prodrug. Following intravenous administration, the prodrug binds to circulating albumin and accumulates in the tumor via the EPR effect. Tumor-associated cathepsin B cleaves the linker extracellularly or intracellularly in lysosomes after endocytosis, releasing the active EGFR inhibitor. EGFR inactivation subsequently triggers cancer-cell death.

While the benefit of albumin binding is well established for small molecular chemotherapeutic agents, to the best of our knowledge, such approaches have not yet been reported for TKIs. To enable this tumor-specific drug release, we selected the well-established cathepsin B-cleavable dipeptide valine-citrulline (ValCit) with a *para*-aminobenzyl carbonyl (PABC) spacer,^32,33^ which is part of several approved antibody drug conjugates (ADCs) for anticancer therapy.^34^ Cathepsin B is a lysosomal cysteine protease that is overexpressed in a variety of cancers.^35^ Moreover, malignant cells frequently secrete the enzyme into the extracellular space, generating a proteolytic tumor microenvironment.^36,37^ As a model TKI, we selected a derivative of the third generation epidermal growth factor receptor (EGFR) inhibitor osimertinib, which is approved for the first-line therapy of EGFR mutation-positive non-small cell lung cancer (NSCLC) (Figure 2A).^38^

**Figure 2:**
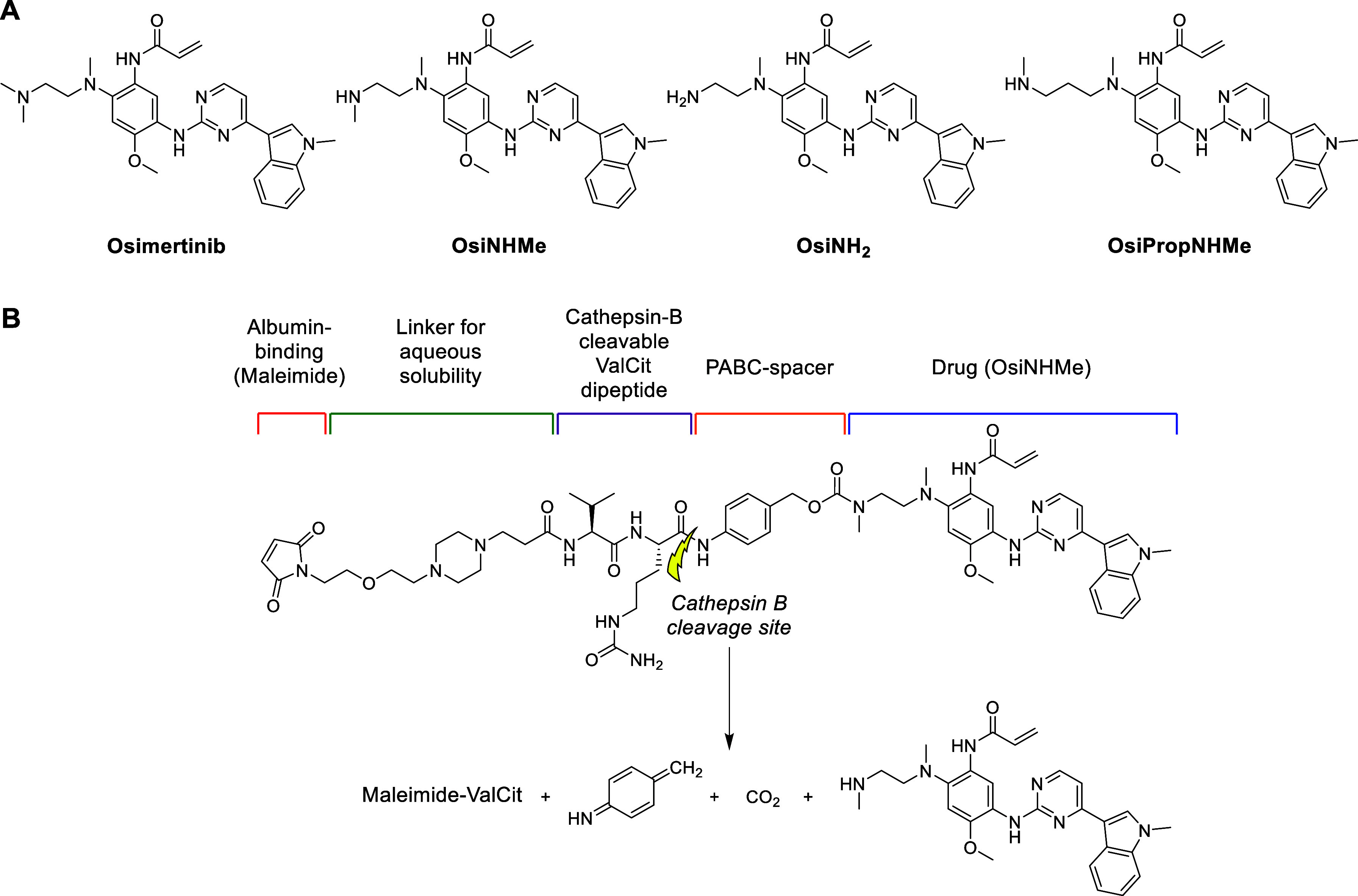
(A) Structure of the approved EGFR inhibitor osimertinib and the derivatives **OsiNHMe**, **OsiNH**_**2**_ and **OsiPropNHMe**. (B) Illustration of the herein presented drug-delivery system (**Mal-Pip-ValCit**). The cathepsin B cleavage site is indicated with a yellow arrow.

Altogether, the system comprises (1) a maleimide moiety (Mal) for binding to endogenous albumin after intravenous administration, (2) a piperazine (Pip) linker motif for enhanced aqueous solubility, (3) a ValCit dipeptide fragment as the cathepsin B-cleavable linker, (4) the PABC spacer to enable enzyme accessibility and (5) the EGFR inhibitor, connected via a carbamate (Figure 2B). Cleavage of the dipeptide C-terminus finally initiates the self-immolation of the PABC fragment, releasing CO_2_ and the free osimertinib derivative (Figure 2B).^32^ As a first step, we synthesized and evaluated several derivatives of osimertinib with a modified dimethylamino group moiety (NMe_2_) to enable conjugation of the EGFR inhibitor. Based on docking experiments, the desired target compound **Mal-Pip-ValCit** (Figure 2B) was synthesized together with a noncleavable reference containing a glycine-glycine (GlyGly) dipeptide motif. For both drugs, kinase screening and cell culture experiments confirmed the prodrug-nature of the new compounds. Subsequently, cathepsin B cleavage and albumin-binding studies were performed. Finally, we demonstrated promising *in vivo* anticancer activity in a cathepsin B-overexpressing, EGFR-dependent xenograft mouse model.

## Results and Discussion

### Synthesis of Osimertinib Derivatives

To enable conjugation of osimertinib to the maleimide-ValCit fragment, three derivatives harboring an NH amine group were synthesized according to an adapted literature procedure (Figure 2A and Scheme S1).^39^ In the first step, a nucleophilic aromatic substitution of *N*-(4-fluoro-2-methoxy-5-nitrophenyl)-4-(1-methyl-1*H*-indol-3-yl)­pyrimidin-2-amine with the respective amine substituents was performed. Next, the aromatic nitro group was reduced to an aniline using Fe and NH_4_Cl in EtOH/H_2_O, creating only a mild pH at which the Boc-groups were stable. The aniline was then acylated using acryloyl chloride in dichloromethane (DCM), whereby slow addition and low temperatures (−80 °C) were essential to avoid diacylation. Finally, acidic deprotection of the Boc group using trifluoroacetic acid (TFA) afforded **OsiNHMe**, **OsiNH**_**2**_ and **OsiPropNHMe**.

### EGFR Inhibition Profile and Anticancer Activity of Osimertinib Derivatives *in Vitro* and *in Vivo*

To investigate the impact of the modifications of osimertinib on the EGFR-inhibitory potential, cell-free kinase inhibition assays were performed on double-mutant EGFR (L858R/T790M) by a commercial provider (Figure S1). The data revealed that **OsiNHMe** (IC_50_ = 1.45 nM) and **OsiNH**_**2**_ (IC_50_ = 1.56 nM) had similar activity to osimertinib, which was the most active compound with an IC_50_ of 1.11 nM. Interestingly, **OsiPropNHMe** was distinctly less active at an IC_50_ of 10.8 nM. To assess whether these cell-free effects translate into biological systems, viability assays using a panel of EGFR-dependent cell lines with known sensitivity to osimertinib were performed (Table 1 and Figure S2). In more detail, the experiments were conducted in the NSCLC cell line H1975, a well-characterized osimertinib-sensitive model carrying the EGFR L858R/T790M double mutation; the NSCLC cell line H1650, which harbors an EGFR exon 19 deletion (E746-A750del); the NSCLC cell line HCC827, which carries the same EGFR exon 19 deletion and is known for high EGFR overexpression; and the epidermoid carcinoma cell line A431, which overexpresses wild-type EGFR. Cells were exposed to increasing concentrations of the drugs for 72 h and their viability was measured by MTT assay. In these experiments, all compounds showed efficacy similar or even slightly greater than that of osimertinib. Of note, our generated IC_50_ values of osimertinib in H1975 cells differed from literature values.^40^ However, when looking at the exact shape of the dose response curves, in both studies an extended plateau exactly in the IC_50_ range can be observed. Thus, we hypothesize that already small variations in the assay protocols (e.g., used cell number, assay incubation time or calculation method) could distinctly impact the IC_50_ values. Consequently, we repeated the viability experiments in this cell line with MTT measurements after different development times. Indeed, incubation with the MTT solution only for 30 min resulted in a ∼10-fold drop of the IC_50_ values: osimertinib (0.3 μM), **OsiNHMe** (0.2 μM) and **OsiNH**_**2**_ (0.2 μM) (Figure S3). Only in case of **OsiPropNHMe** the values remained largely unchanged. Overall, this effect can probably be associated with the rather cytostatic than cytotoxic activity of EGFR inhibition.

**Table 1:** Half-Maximal Inhibitory Concentration (IC_50_) Values ± Standard Deviation Determined after 72 h of Drug Treatment

cell line	H1975	H1650	HCC827	A431
osimertinib	3.92 ± 2.3	5.18 ± 1.1	<1	2.90 ± 0.98
OsiNHMe	2.48 ± 1.2	3.24 ± 0.6	<1	2.42 ± 1.0
OsiNH_2_	2.71 ± 1.6	3.21 ± 0.1	<1	1.73 ± 0.8
OsiPropNHMe	4.41 ± 1.2	3.16 ± 0.2	<1	2.29 ± 0.9

Consequently, we decided to check, how the *in vitro* antitumor activity translates into the *in vivo* situation. To this end, therapy experiments on H1975 xenografts in C.B.-17^SCID/SCID^ mice were performed (Figure 3). All drugs were administered via oral gavage once daily, five times a week, for 2 weeks at doses equimolar to 29.8 mg/kg osimertinib mesylate, which corresponds to 25 mg/kg osimertinib, the reported maximum tolerated dose (MTD).^41^

**Figure 3:**
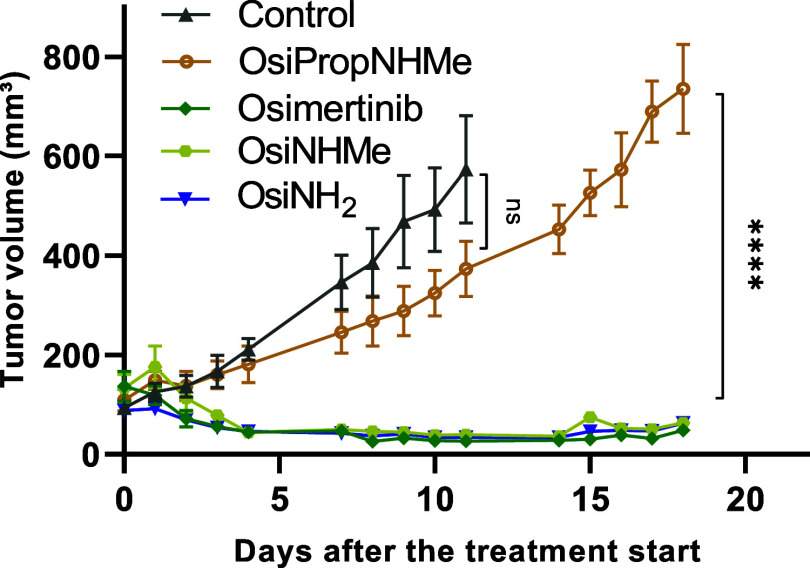
Anticancer activity against H1975 xenografts in C.B.-17^SCID/SCID^ mice. Impact on tumor growth. Data are presented as means ± SEM. Drugs were administered by oral gavage once daily, five times per week for 2 weeks (day 1–5 and day 7–11). **OsiNHMe**, **OsiNH**_**2**_, and **OsiPropNHMe** were dosed at levels equimolar to osimertinib mesylate, dosed at 29.8 mg/kg. Significance was calculated using one-way ANOVA and Dunnett’s multiple comparison test (ns-non significant, ****p < 0.0001).

Treatment revealed that the tumor growth-inhibiting activity of both **OsiNHMe** and **OsiNH**_**2**_ was similar to osimertinib, while **OsiPropNHMe** was distinctly less effective. These data of **OsiNHMe** are in good agreement with the literature, reporting that this metabolite of osimertinib retains a similar potency and selectivity profile.^39^ Moreover, these experiments revealed that both **OsiNHMe** and **OsiNH**_**2**_ could be potential candidates for prodrug strategies, while **OsiPropNHMe** does not have sufficient EGFR-targeting properties. This is very surprising, considering that only one additional CH_2_ unit was introduced in the region extending into the solvent front, while the moiety interacting with the binding site remained unchanged. Thus, we selected **OsiNHMe** as the structurally closest osimertinib derivative for the synthesis of the prodrugs (see below).

### Molecular Docking and Dynamics Studies

The intended prodrug system for **OsiNHMe** comprises the PABC spacer, the ValCit dipeptide as the trigger unit for cathepsin B and the maleimide moiety for albumin binding (Figure 2B) with a noncleavable GlyGly derivative as a reference. To evaluate the viability of the prodrug concept, in a first step in silico molecular docking studies of **Mal-Pip-ValCit** and **Mal-Pip-GlyGly** with the EGFR were performed. Building on our previous investigation of an EGFR-inhibiting oxaliplatin­(IV) complex,^42^ the double mutant L858R/T790M EGFR kinase domain (PDB ID: 5CAS)^43^ was used. As a reference, osimertinib was docked into the EGFR-binding site, where it adopted a pose consistent with that of the cocrystallized ligand (Figure S4A), and achieved a comparable docking score (Table 2). Also, **OsiNHMe** fitted very well into the EGFR-binding pocket, with its 1-methyl-3-pyrimidin-indole pharmacophore engaging the active site similarly to osimertinib. Notably, the third-best pose, as well as most of the top ten ranked poses, closely superimpose with that of osimertinib (Figure 4A). Although the top two do not perfectly overlap, they remain within the binding pocket and show comparable docking scores (Table 2) making them equally plausible binding modes. Conjugation of osimertinib to the peptide-PABC moiety significantly altered the binding behavior. The docking results clearly showed that both **Mal-Pip-ValCit** and **Mal-Pip-GlyGly** (Figure 4B,C) prodrugs failed to position the 1-methyl-3-pyrimidin-indole scaffold within the EGFR-binding pocket. Instead, these bulky conjugates adopted poses outside the active site, and their docking scores are markedly lower than those of osimertinib and **OsiNHMe**, indicating a substantial reduction in binding affinity (Table 2). Among the top ten poses, only one for **Mal-Pip-ValCit** (2nd-best pose) and two for **Mal-Pip-GlyGly** (7th and 8th-best poses) position the pharmacophore within the pocket, and even in these cases, the docking scores are low. Molecular dynamics (MD) simulations were performed to evaluate the stability of the docked conformations of the prodrugs relative to **OsiNHMe**. The root-mean-square deviation (RMSD, Å) profiles (Figure S4B–D) indicate that, over the 100 ns simulation, **OsiNHMe** retains its docking pose with minimal fluctuation (<0.8 Å), consistent with a stable ligand–receptor interaction. In contrast, **Mal-Pip-ValCit** and **Mal-Pip-GlyGly** exhibit larger positional deviations (approximately 4 Å), consistent with weaker binding affinity. These compounds remain with the pharmacophore largely displaced from the active site, instead sampling the outer protein surface. Overall, these findings suggest that the prodrug strategy could effectively mask their ability to bind to the EGFR.

**Table 2:** Docking Scores of the Interactions of Osimertinib, **OsiNHMe**, **Mal-Pip-ValCit** and **Mal-Pip-GlyGly** With Mutant EGFR (PDBid: 5CAS, Green Surface)

molecule	docking score (kcal/mol)
cocrystallized ligand (redocked)	–9.20
osimertinib	–9.01
OsiNHMe	–9.30
OsiNHMe^a^	–9.17
Mal-Pip-GlyGly	–3.80
Mal-Pip-ValCit	–2.41

a 3rd pose is the first in the same orientation as osimertinib.

**Figure 4:**
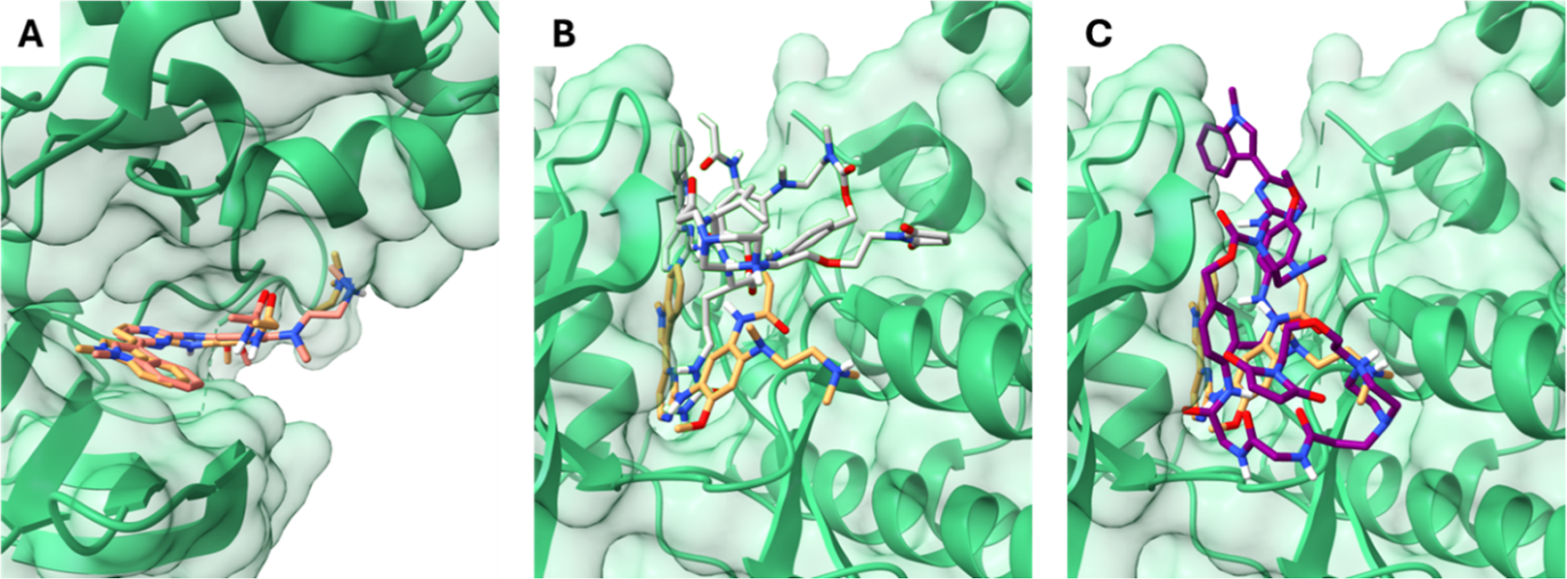
*In silico* docking studies of osimertinib, **OsiNHMe**, **Mal-Pip-ValCit** and **Mal-Pip-GlyGly** to the EGFR. 3D representation of mutant L858R/T790M EGFR (PDB ID: 5CAS, green surface) interacting with osimertinib (in gold) and (A) **OsiNHMe** (in salmon), (B) **Mal-Pip-ValCit** (in gray) and (C) **Mal-Pip-GlyGly** (in purple).

### Synthesis of the Albumin-Binding, Cathepsin B-Cleavable EGFR-Inhibitor Prodrugs

The target compounds **Mal-Pip-ValCit** and **Mal-Pip-GlyGly** were synthesized in nine steps via a universal precursor with a *para*-nitrophenol (PNP) active ester moiety, which enabled late-stage incorporation of amine-functionalized molecules (Scheme 1). To prevent Michael addition side reactions, the maleimide was protected through a Diels-Alder reaction with 2,5-dimethylfuran, which can conveniently be removed by heating at ∼90 °C.^44^ For enhanced aqueous solubility, a protonatable piperazine moiety was introduced as a linker between the maleimide and ValCit dipeptide, as recently reported by our group.^45^ Briefly, after 2,5-dimethylfuran protection of maleimide, alkylation with 2-bromoethyl ether to yield pMal-O-Br was performed. Next, piperazine was introduced to generate pMal-O-Pip. Subsequent coupling with *tert*-butyl 3-bromopropionate gave pMal-O-Pip-COO^*t*^Bu. Finally, ^*t*^Bu-deprotection yielded pMal-O-Pip-COOH in 28% yield over five steps (Scheme 1). Notably, heating at 50 °C during the second step reduced the endo isomer content from 16 % (observed immediately after the Diels-Alder reaction) to 2 %, due to maleimide deprotection. This highlights the importance of a high excess of the exoisomer (thermodynamic product) to prevent product loss from cycloreversion of the endoisomer (kinetic product). Next, a coupling reaction of pMal-Pip-COOH with H_2_N-GlyGly-PAB-OH (synthesized in 2 steps) or commercially available H_2_N-ValCit-PAB-OH was performed using the coupling agent 1-ethyl-3-(3-dimethylaminopropyl)­carbodiimide hydrochloride (EDC·HCl) with *N*-hydroxybenzotriazole (HOBt). The benzyl alcohols were then activated using a slight excess of bis­(4-nitrophenyl) carbonate (3 equiv) to generate the respective PNP-active esters, which were purified via column chromatography using a MeOH/DCM mixture as the eluent. Interestingly, during solvent removal after chromatographic purification, the initially colorless solutions turned bright yellow, indicating the release of PNP. Mass spectrometry confirmed the formation of the respective methyl carbonate esters, formed by nucleophilic substitution of the MeOH from the eluent upon DCM evaporation (Figure S5). The reaction was likely promoted by the piperazine amines which create basic conditions similar to those induced by other tertiary amines. However, adding high-boiling toluene to the mixture during solvent removal prevented the concentration of MeOH and thus suppressed the substitution reaction (Figure S6). Both PNP-derivatives were obtained in excellent yields (∼90%) and subsequently reacted with **OsiNHMe** (Figure 2A). Finally, maleimide deprotection was achieved by heating in DMSO at 90 °C for 3 h, followed by purification via preparative high-performance liquid chromatography (HPLC). The desired compounds were obtained in good yields over the last four steps (**Mal-Pip-GlyGly**: 40%, **Mal-Pip-ValCit**: 35%).

**Scheme 1:**
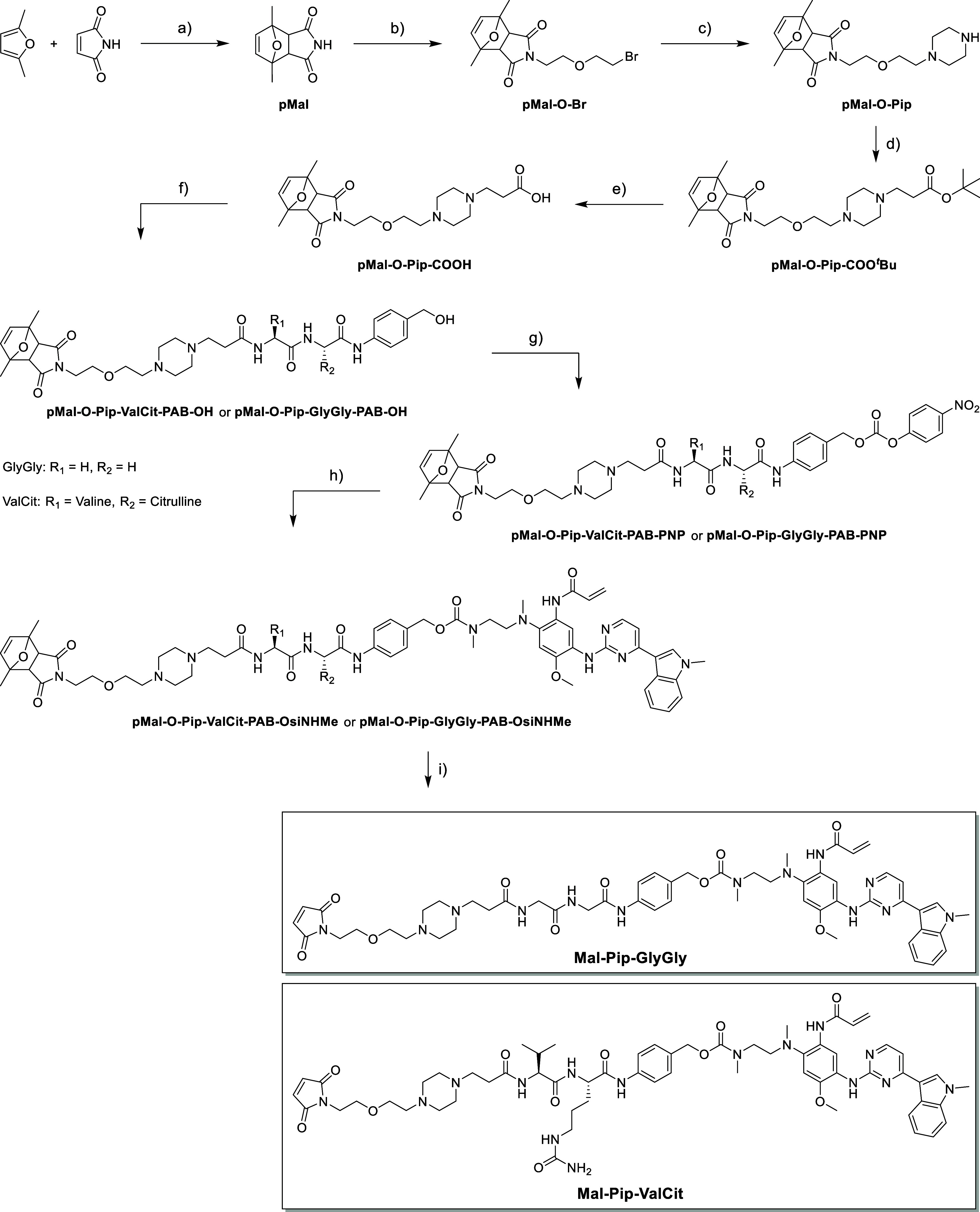
Synthesis of the Target Compounds **Mal-Pip-GlyGly** and **Mal-Pip-ValCit** ^a^

a Reaction conditions: (a) MeCN, 60 °C (yield: 95%); (b) 2-bromoethyl ether, K_2_CO_3_ in *N*,*N*-dimethylformamide (DMF), 50 °C (yield: 49%); (c) piperazine, K_2_CO_3_ in DMF, 50 °C (yield: 86%); (d) *tert*-butyl 3-bromopropionate, NEt_3_ in CHCl_3_, 0 °C → room temperature (yield: 77%); (e) HCl in dioxane, room temperature (yield: 92%); (f) H_2_N-GlyGly-PAB-OH (1) or H_2_N-ValCit-PAB-OH (2), EDC·HCl, HOBt, NEt_3_ in dry DMF, room temperature (yield 1: 76%, yield 2: 66%); (g) bis­(4-nitrophenyl) carbonate, DIPEA in dry DMF, room temperature (yield 1: 97%, yield 2: 88%); (h) **OsiNHMe**, DIPEA in dry DMF, room temperature (yield 1: 85%, yield 2: 92%); (i) DMSO, 90 °C (yield **Mal-Pip-GlyGly**: 64%, yield **Mal-Pip-ValCit**: 65%).

After successful synthesis, the solubility of the target compounds was tested in aqueous isotonic media (e.g., 0.9% NaCl or 5% glucose), as required for intravenous (i.v.) administration in later *in vivo* studies. As mentioned above, in mice, the MTD of osimertinib mesylate is 29.8 mg/kg. Therefore, the target compounds needed to be soluble at ∼10 mM to enable equimolar dosing of the prodrugs. Complete dissolution was easily achieved using 20 vol % propylene glycol in a 5% glucose solution, with no precipitation observed for >24 h.

Of note, the target compounds were also synthesized harboring a PEG_6_-linker (Scheme S2). Unfortunately, neither compound was soluble at the required concentration of 10 mM, even in the presence of various solubilizing additives at their maximum tolerable concentration (20% propylene glycol or 30% PEG400). Thus, **Mal-PEG-GlyGly** and **Mal-PEG-ValCit** were omitted from further investigations.

### Stability, Kinase Inhibition and Anticancer Activity of Mal-Pip-ValCit and Mal-Pip-GlyGly *in Vitro*

First the stability of the **Mal-Pip-ValCit** and **Mal-Pip-GlyGly** prodrugs (10 μM) was tested in 10 mM phosphate buffer (PB) at pH 7.4 and 37 °C using analytical HPLC. Apart from the expected maleimide hydrolysis (Figure S7), both compounds remained stable for >25 h (Figure S8). Next, we investigated whether the new compounds are indeed prodrugs, unable to inhibit the EGFR (without cathepsin B activation). To this end, the EGFR-inhibitory potential of **Mal-Pip-GlyGly** and **Mal-Pip-ValCit** was evaluated in a cell-free kinase inhibition assay (Figure S9). The data revealed a complete loss of EGFR-inhibitory activity with IC_50_ values >10 μM, in contrast to the potent **OsiNHMe** activity of 1.45 nM (Figure S1). In good agreement, also in the viability assays using EGFR mutant H1975 cells the observed IC_50_ values shifted from 3.92 μM and 2.48 μM for free osimertinib and **OsiNHMe**, respectively, to >10 μM for both prodrugs after 72 h incubation (Figure 5). To evaluate the effects of the new drugs in healthy cells, we performed viability assays with murine fibroblasts (Figure 5). Noteworthy, in contrast to osimertinib, both prodrugs had no relevant impact on the viability of these cells. Together these data confirm that the new compounds are indeed stable prodrugs, without any premature release of **OsiNHMe**, which might prevent off-target toxicity of osimertinib in healthy tissue.

**Figure 5:**
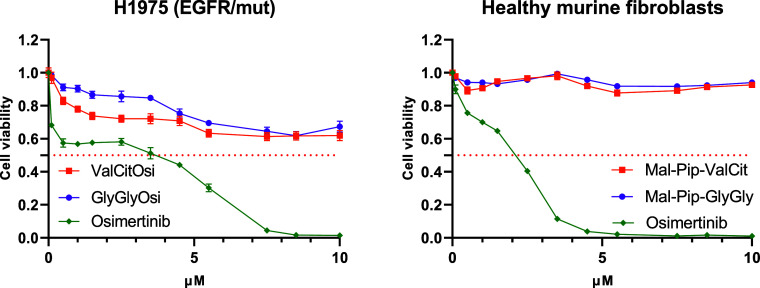
Activity of the new EGFR-inhibitor prodrugs in H1975 cells and healthy murine fibroblasts after 72 h drug treatment. Cell viability was assessed using MTT assays. Drug response curves were normalized to untreated control cells. Data were pooled from three independent experimental replicates, each yielding comparable results. Data are expressed as mean ± SEM.

### Cathepsin B Cleavage Studies

Cathepsin B cleavage studies with **Mal-Pip-ValCit** and **Mal-Pip-GlyGly** were performed to demonstrate enzyme accessibility and prodrug activation. Therefore, cathepsin B from human liver was activated in a solution of DL-dithiothreitol (DTT) and ethylenediaminetetraacetic acid (EDTA) and subsequently incubated with the compounds in sodium acetate buffer at pH 5 (Figure S10).^46^ Of note, the added DTT quickly reacted with the maleimide moiety of **Mal-Pip-ValCit** and **Mal-Pip-GlyGly** in a Michael addition reaction, bridging two molecules via the DTT-thiol groups (Figure S11). However, this process did not influence the catalytic reaction, as after only 30 min ca. half of **Mal-Pip-ValCit** was cleaved and **OsiNHMe** released (Figure S12). After 1.5 h the cleavage was complete. In contrast, **Mal-Pip-GlyGly** was completely stable in the presence of cathepsin B and no release of **OsiNHMe** was observed (Figure S13A). As additional control samples, **Mal-Pip-ValCit** and **Mal-Pip-GlyGly** were incubated with DTT and EDTA without cathepsin B, revealing no release of **OsiNHMe** (Figure S13B,C). In order to evaluate the stability of the new drugs in cell culture conditions, we tested their activity in the cell model Caki-1 (Figure S14), which is characterized by very high levels of lysosomal cathepsin B (Figure S15).^46^ In contrast to osimertinib, both prodrugs had no significant activity for up to 72 h indicating their distinct intracellular stability despite the high lysosomal cathepsin B expression of the cells. This can presumably be explained by the inability of the prodrugs to reach the lysosomes due to their albumin-mediated drug uptake via endocytosis.

### Albumin-Binding Studies

To investigate the albumin-binding properties of **Mal-Pip-ValCit** and **Mal-Pip-GlyGly**, HSA (∼300 μM) was incubated with the drugs in a 6:1 ratio in PB at pH 7.4 and 37 °C, and analyzed via HPLC. For both **Mal-Pip-ValCit** and **Mal-Pip-GlyGly** (Figure S16A,B), already after 10 min >95% were albumin-bound, accompanied by a significant increase in the albumin peak area. A very small additional peak appeared, likely corresponding to the hydrolyzed maleimide derivatives. The albumin conjugates remained stable for >24 h (data not shown). As TKIs like osimertinib are well-known to bind to albumin also through electrostatic interaction,^23,47^ an additional experiment was performed, blocking the maleimide of **Mal-Pip-ValCit** with *N*-acetylcysteine (NAC) prior to incubation with albumin (Figure S16C). No albumin binding was detected for >2 h. This confirmed that the rapid binding observed with the free maleimide (Figure S16A,B) is not electrostatic, since such adducts would dissociate under HPLC conditions and remain detectable as a separate peak.

Next, we explored the cathepsin B-mediated release of the EGFR inhibitor from the albumin conjugate (Figure S17). Therefore, we incubated **Mal-Pip-ValCit** with HSA (∼300 μM) and added cathepsin B (NaOAc buffer, pH 5) similarly to the cathepsin B cleavage assay above. To follow the reaction, we selected 375 nm as the detection wavelength, which is characteristic for **OsiNHMe**. At the 0 h time point, ∼85% of **Mal-Pip-ValCit** was bound to albumin and ∼10% of hydrolyzed **Mal-Pip-ValCit** were observed. After 4 h of incubation with cathepsin B, ∼75% of the EGFR inhibitor was released while ∼20% remained associated with the albumin peak. This experiment clearly confirmed efficient drug release from the albumin-drug conjugate.

The Cys^34^-conjugation of maleimide was investigated by incubation of **Mal-Pip-ValCit**, **Mal-Pip-GlyGly** and **OsiNHMe** with albumin at 37 °C and the remaining free thiol content was determined via UV–vis spectrophotometry using 2,2′-dithiodipyridine (DTDP).^48^ The initial levels of free Cys^34^ in the used HSA solution was 33 ± 3%, which was subsequently normalized to 100%. Coincubation of **Mal-Pip-GlyGly** or **Mal-Pip-ValCit** gradually reduced the free thiol content (Figure 6). In contrast, **OsiNHMe**, without a maleimide moiety, did not affect the quantity of free Cys.^34^ However, the binding at Cys^34^ was not quantitative with 61% **Mal-Pip-GlyGly** and 53% **Mal-Pip-ValCit** bound at a 1:1 ratio. In general, the maleimide-Cys^34^ interaction is considered rather fast.^48^ Therefore, the binding kinetics of **Mal-Pip-GlyGly** to Cys^34^ was separately investigated at a 1:1 ratio, revealing that the molecule was already bound to Cys^34^ after 20 min (60%) and did not increase considerably (62%) after a further 70 min of incubation (Figure S18).

**Figure 6:**
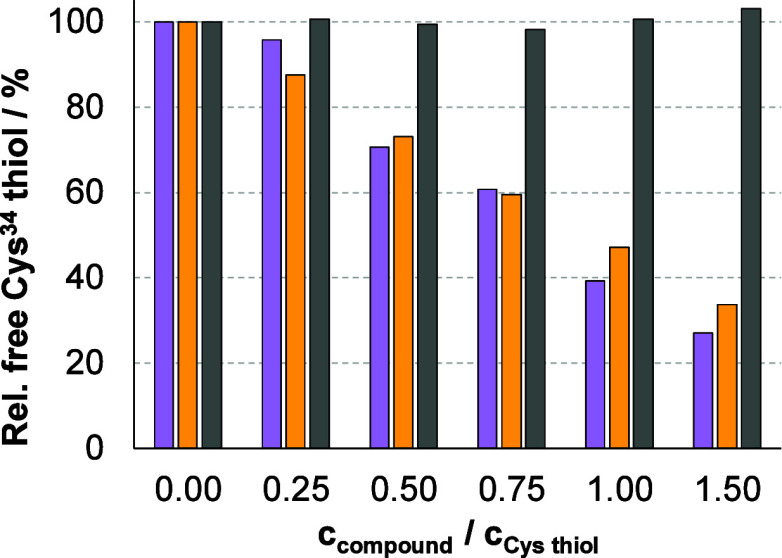
Relative free Cys^34^ thiol content in HSA at various equivalents of **Mal-Pip-GlyGly** (purple), **Mal-Pip-ValCit** (orange) and **OsiNHMe** (gray) based on the DTDP assay. [*c*_Cys^34^_ = 6.6 μM; (*c*_HSA_ = 20 μM); *t* (incubation HSA-compound) = 20 min; pH = 7.40 (100 mM PB) at 37 °C].

Given the discrepancy between the efficient and covalent albumin binding of the prodrug (Figure S16) and the high amount of free thiol (∼40%) of albumin after equimolar incubation with **Mal**-**Pip-ValCit** (Figure 6), most likely nucleophilic groups other than Cys^34^ are involved. We tested this theory by repeating the DTDP assay (Figure 6) with **Mal-Pip-ValCit** at pH 6 instead of pH 7.4, which further protonates lysine residues and prevents them from engaging in aza-Michael addition reactions (with maleimide). Indeed, ∼20% higher binding to the albumin-SH was observed, indicating an involvement of lysines in covalent maleimide binding to albumin at pH 7.4 (data not shown). Of note, Cys^34^ is situated in a cleft in subdomain IA of HSA, which could hinder its fast interaction with large molecules. However, HSA harbors several hydrophobic binding pockets.^49^ Thus, the compound may partially bind electrostatically to one of the other albumin pockets, where it can react with lysine residues rather than reaching the Cys^34^ pocket.

### Serum Extraction of Osimertinib and OsiNHMe

To determine the stability of the prodrug in circulation, the common strategy is the quantification of the free TKI in serum. Therefore, both osimertinib (in accordance with existing protocols^50^) and **OsiNHMe** were spiked into mouse serum, precipitated with an excess of acetonitrile and after centrifugation the supernatant was analyzed via HPLC. The first time point immediately after sample preparation revealed high recovery of the EGFR inhibitors (>95%). However, extended incubation times for up to 24 h at 37 °C resulted in a distinct progressive decrease of the extracted concentrations of both osimertinib and **OsiNHMe** (Figure 7A,B). Also, when using a pure HSA solution (∼600 μM in PB) a similar behavior was observed, indicating fast covalent albumin binding (Figure 7A,B). Notably, covalent conjugation of **OsiNHMe** to albumin was not detected under cathepsin B cleavage conditions at ∼pH 5 (data not shown). In mouse serum the recovery of both osimertinib and **OsiNHMe** was even less than 50% after 2 h of incubation and only ∼10% after 24 h. To confirm the nature of this interaction, 50 μM osimertinib was incubated with albumin (∼1.5 mM) at 37 °C for 24 h, purified using size-exclusion chromatography and subsequently analyzed by mass spectrometry (Figure 7C). The data clearly revealed covalent conjugation of osimertinib to HSA.

**Figure 7:**
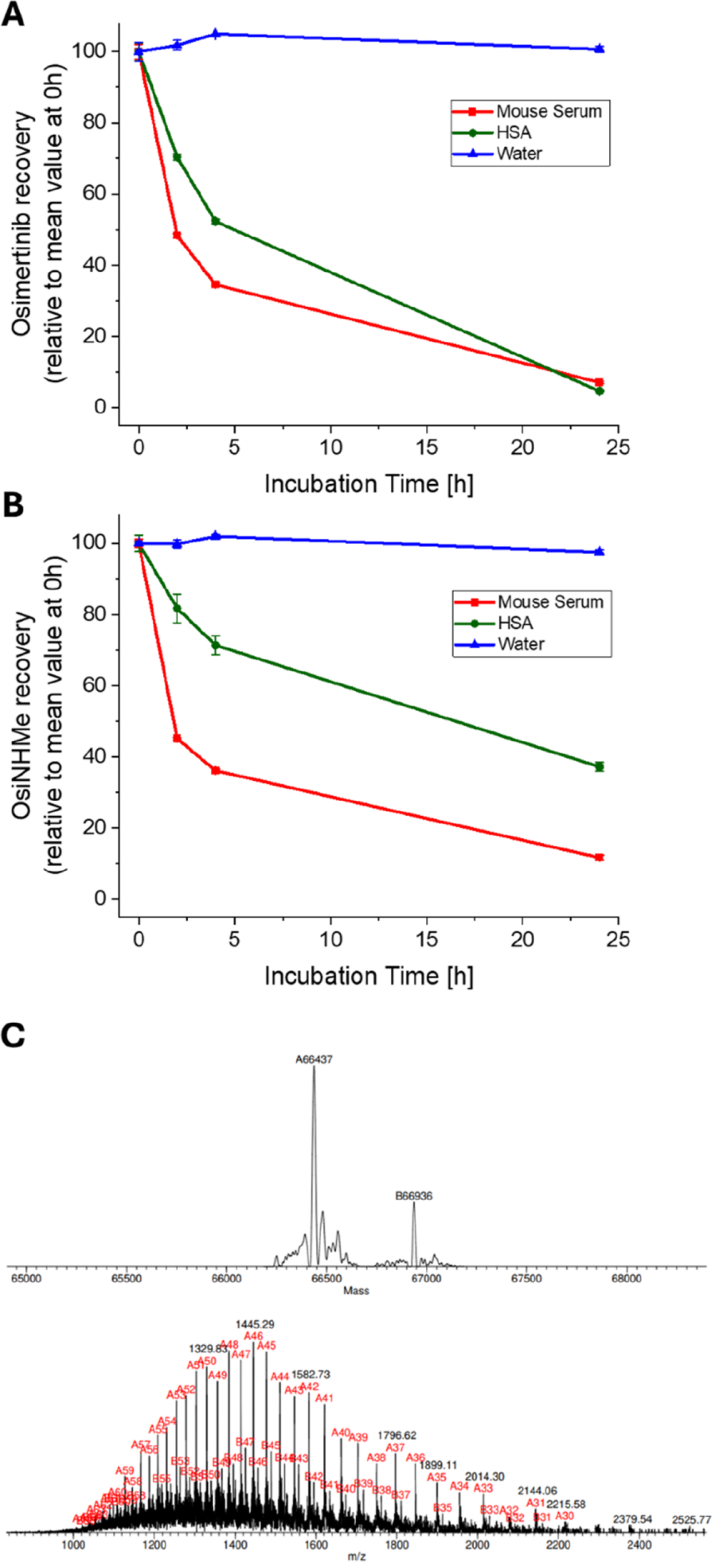
Recovery of (A) osimertinib and (B) **OsiNHMe** over 24 h of incubation at 37 °C in Milli-Q water (blue), ∼600 μM HSA (green; Albunorm in 150 mM PB, pH = 7.4) or mouse serum from Balb/c mice. Each time point was determined from the mean of triplicate measurements; error bars indicate the standard deviation (SD). (C) Deconvoluted mass spectrum of the HSA-osimertinib conjugate after LC–MS separation, acquired with in-source collision-induced dissociation (isCID). Signals at 66437 and 66936 Da correspond to native HSA and the HSA-osimertinib adduct, respectively.

These data are well in agreement with recent literature reports, showing that osimertinib covalently conjugates to different lysine residues of albumin^51^ and that this reaction is irreversible.^52^ Unfortunately, this prevented quantitative serum stability studies or detailed pharmacokinetics of the prodrugs **Mal-Pip-ValCit** and **Mal-Pip-GlyGly**, as the released inhibitor binds covalently to albumin.

### Tolerability and anticancer activity of Mal-Pip-ValCit and Mal-Pip-GlyGly *in Vivo*

To select an appropriate cancer model for *in vivo* activity studies, we first assessed the tissue cathepsin B expression of different xenograft tumors (H1975, H1650 and HCT116) by immunohistochemistry. Analysis of stained sections using QuPath 0.5.1 software revealed that H1650 xenografts exhibited the strongest cathepsin B levels (Figure 8A,C). In parallel to the cathepsin B stains, also albumin uptake into the tumor tissue was confirmed by immunohistochemistry (Figure 8B).

**Figure 8:**
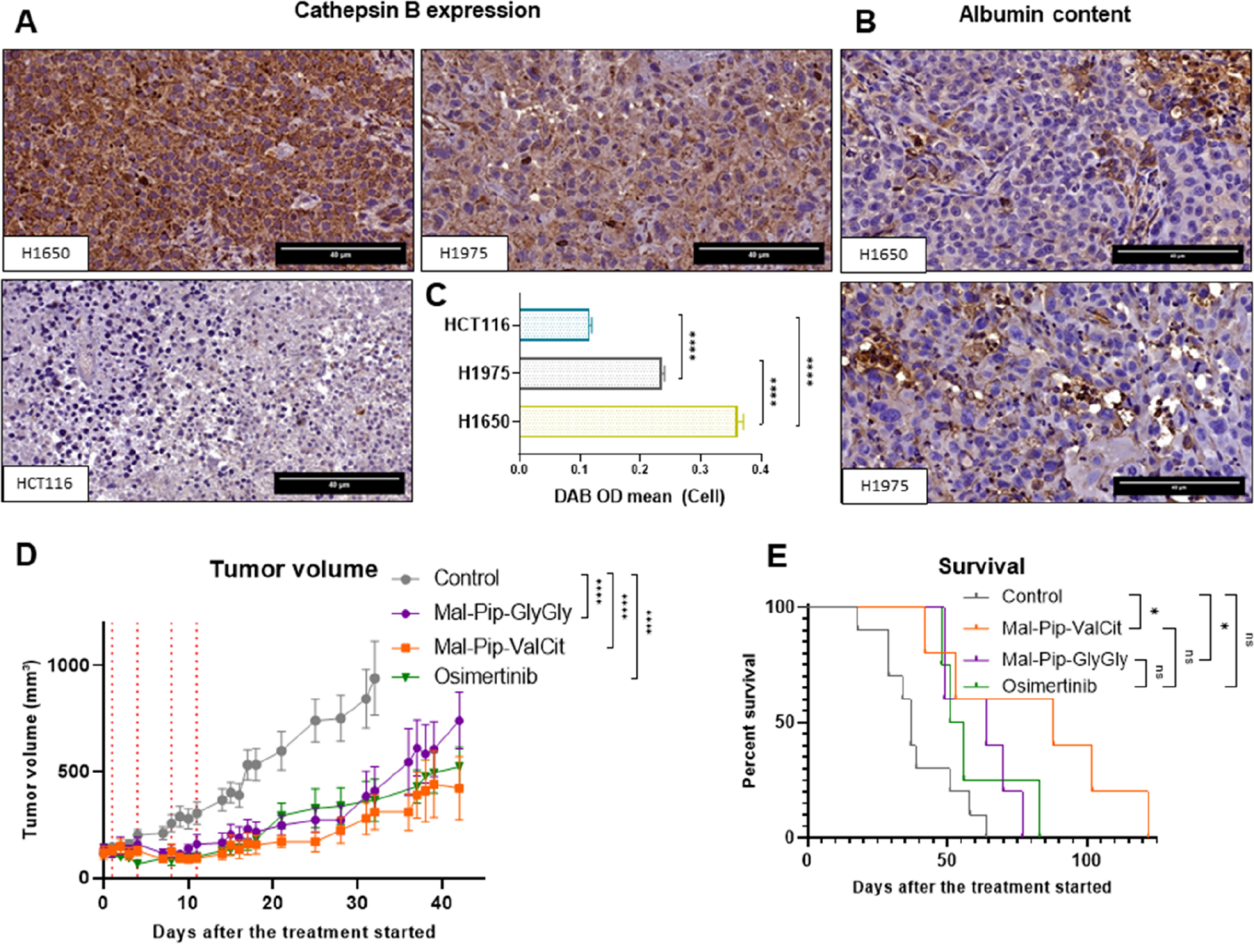
Cathepsin B expression and albumin content in tumor tissues as well as antitumor activity against H1650 xenografts *in vivo*. Representative images of tumor sections from each slide, corresponding to the indicated cell line, presented at 60× magnification. (A) Stained for cathepsin B expression and (B) stained for albumin content. (C) Quantification of cathepsin B expression and albumin content in tumor tissues derived from mouse xenografts. The analysis was based on histological evaluation, including H&E staining for general tissue morphology and immunohistochemical staining specifically targeting cathepsin B and albumin. Staining intensity levels were measured as the mean optical density (OD) of 3,3′-diaminobenzidine (DAB) in cellular regions (brown areas), using the image analysis software QuPath (0.5.1). Accurate quantification was achieved through the software’s stain vector estimation (color deconvolution), which allowed for precise separation of DAB signal from hematoxylin background. Statistical comparison was performed using an unpaired *t*-test. Error bars represent mean ± SEM ****p < 0.0001. (D) Impact on tumor growth. H1650 cells were grown s.c. in C.B.-17^SCID/SCID^ mice. When the tumors reached the size of ∼125 mm^3^, animals were treated i.v. twice per week with equimolar doses of **Mal-Pip-ValCit** (83.7 mg/kg), **Mal-Pip-GlyGly** (75.7 mg/kg) or p.o. with osimertinib mesylate (29.8 mg/kg) for 2 weeks (days indicated by dotted lines). Data are presented as mean ± SEM. Curves are always shown until the first of the mice in the respective test group had to be sacrificed, statistical significance was determined using Tukey’s multiple comparisons test. ****p < 0.0001 (E) Overall survival of the animals depicted via Kaplan–Meier survival curve. Statistical significance was determined using log-rank test and Mantel–Cox post-test. *p < 0.05.

Based on their high cathepsin B expression, H1650 cells were selected as model for the evaluation of the new prodrugs *in vivo* (Figure 8D,E). All drugs were administered at doses equimolar to osimertinib mesylate (29.8 mg/kg) twice per week. Indeed, treatment with all three TKIs resulted in sustained and significant tumor growth inhibition. **Mal-Pip-ValCit** was more effective than **Mal-Pip-GlyGly** and even osimertinib alone, which supports the expected albumin-mediated accumulation of the prodrug in the tumor. Accordingly, **Mal-Pip-ValCit** treatment resulted in a significant survival benefit (>100%) compared to the control. While animals in the control, **Mal-Pip-GlyGly**, and osimertinib groups reached average survival relatively fast (within 39 days for the control group, 70 days for **Mal-Pip-GlyGly**, and 55 days for osimertinib), those receiving **Mal-Pip-ValCit** survived considerably longer (average 90 days), remaining viable for up to 122 days. The clear survival gap between **Mal-Pip-ValCit** and **Mal-Pip-GlyGly** underscores the biological relevance of linker activation *in vivo*, where cathepsin B activity enables the selective release of the cytotoxic payload. In contrast, **Mal-Pip-GlyGly**, which cannot be enzymatically cleaved by cathepsin B, is significantly less active in the tumor environment despite its albumin-mediated delivery to the tumor. Notably, **Mal-Pip-GlyGly** still showed significant antitumor activity compared to the control, which may result from activation during physiological degradation of albumin.^53^ Importantly, no significant weight loss (Figure S19) or signs of systemic toxicity (Figure S20) were observed during the course of treatment, indicating good tolerability. Together, this indicates that incorporation of TKIs in albumin-targeted cathepsin B-cleavable prodrug systems, are a valuable strategy to improve their antitumor activity.

## Conclusion

The exceptional pharmacokinetic properties of albumin, including its high plasma concentration, long circulatory half-life and ability to accumulate in tumor tissue, highlight the protein as an important vehicle for the targeted delivery of anticancer therapeutics. Many endogenous ligands and drugs, including TKIs, are known to electrostatically bind to albumin. However, in contrast to covalently bound therapeutics, there is no direct evidence that electrostatic albumin association of TKIs also leads to enhanced tumor targeting, beyond the well-known effect of prolonged systemic circulation. Moreover, according to the free drug hypothesis, only the unbound drug fraction in the body is able to interact with its pharmacological target. Together, this suggests that the activity of TKIs *in vivo* may even be reduced because of their strong electrostatic binding to albumin. Notably, for osimertinib, both our data and recent literature reports additionally indicate extensive covalent binding to albumin lysines^51^ and an inability to reverse this reaction.^52^ This further reduces the availability of osimertinib for target inhibition. Consequently, this study introduces a drug delivery system for TKIs that allows their covalent attachment to the Cys^34^ residue of endogenous albumin via a maleimide moiety after intravenous administration. To enable tumor-specific release, a cathepsin B-cleavable ValCit dipeptide coupled to a PABC spacer was employed. For the selection of the active component, three derivatives of the EGFR inhibitor osimertinib were synthesized, harboring an NH group for attachment to the ValCit-PABC fragment, and the most promising candidate **OsiNHMe** was identified after evaluation *in vitro* and *in vivo*. The target compound **Mal-Pip-ValCit** efficiently released **OsiNHMe** in the presence of cathepsin B and demonstrated excellent stability in the absence of the enzyme. As expected, the reference compound **Mal-Pip-GlyGly** was unaffected in either condition. The prodrug nature of **Mal-Pip-ValCit** and **Mal-Pip-GlyGly** was demonstrated in a cell-free kinase inhibition assay against double mutant EGFR (L858R/T790M), as no IC_50_ value could be determined within the tested concentration range (up to 10 μM). This finding was further supported by a cell-based MTT assay. Binding studies revealed efficient covalent conjugation of both prodrugs to albumin. Finally, *in vivo* studies were performed in EGFR-mutant H1650-bearing mice, strongly overexpressing cathepsin B. Indeed, **Mal-Pip-ValCit** demonstrated the strongest tumor regression as well as the longest overall survival when directly compared to **Mal-Pip-GlyGly** and osimertinib. These data underscore the increased antitumor activity of a TKI when covalently conjugated to albumin over electrostatic binding in blood, guiding future strategies for improved therapeutics.

## Experimental Part

### Chemicals

pMal-O-Pip-COOH·2HCl was synthesized according to literature.^45^ The synthetic strategies of **OsiNHMe**, **OsiNH**_**2**_, **OsiPropNHMe** ^39^ and pMal-PEG_6_-COOH^54^ were adapted from literature. Milli-Q water (18.2 MΩ cm, Merck Milli-Q Advantage, Darmstadt, Germany) was used for synthesis and for analytical and preparative RP-HPLC. Anhydrous solvents (DCM and DMF) over molecular sieves were purchased from Thermo Scientific. All other chemicals and solvents were purchased from commercial suppliers (Aaron Chemicals, abcr, Acros Organics, Alfa Aesar, Ambeed, BLD Pharm, Fisher Scientific, Fluka, Sigma-Aldrich and TCI Europe, VWR) and used without purification. Silica gel (particle size 40–63 μm) for column chromatography was purchased from VWR. No unexpected or unusually high safety hazards were encountered. Electronspray ionization (ESI) mass spectra for intermediate compounds were recorded on a Bruker amaZon speed ETD mass spectrometer and high-resolution mass spectra of final compounds were measured on an Orbitrap Exploris 120 mass spectrometer in positive mode at the Mass Spectrometry Centre of the University of Vienna. ^1^H NMR spectra of intermediate compounds were recorded in deuterated dimethyl sulfoxide (DMSO-*d*_6_, 99.8% D, purchased from Eurisotop) at 25 °C on a Bruker FT-NMR spectrometer AV NEO 500 at 500.10 MHz. For the characterization of the final compounds **OsiNHMe**, **OsiNH**_**2**_, **OsiPropNHMe**, **Mal-PEG-ValCit**, **Mal-PEG-GlyGly**, **Mal-Pip-ValCit** and **Mal-Pip-GlyGly** dry DMSO-*d*_6_ from glass ampules (99.8% D, purchased from Eurisotop) was used. One- (^1^H and ^13^C) and two-dimensional (COSY, HSQC and HMBC) NMR spectra were recorded at 25 °C using a Bruker FT-NMR spectrometer AV NEO 500 MHz. ^1^H NMR spectra were measured at 500.32 MHz and ^13^C NMR spectra at 125.82 MHz. All NMR spectra were recorded at the NMR Centre of the University of Vienna. Chemical shifts (ppm) were referenced internally to the residual solvent peaks. For the description of the spin multiplicities the following abbreviations were used: s = singlet, d = doublet, t = triplet, q = quartet, bs = broad singlet, dd = doublet of doublets, dt = doublet of triplets, quint = quintet, sxt = sextet, m = multiplet. The ^1^H, ^13^C, COSY, HSQC and HMBC NMR spectra of the final compounds are depicted in Figures S21–S27 and the HPLC runs in Figures S28 and S29. All compounds are >95% pure by HPLC. Purification by preparative HPLC was performed on an XBridge BEH C18 OBD Prep Column (19 mm × 250 mm) on an Agilent 1260 Infinity II system. Flow rates of 17 mL/min were constant for each run at 20 °C. Elemental analysis measurements were performed on a Eurovector EA 3000 CHNS–O Elemental Analyzer at the Microanalytical Laboratory of the University of Vienna and are within ± 0.4%, confirming >95% purity.

### Synthesis

#### *Tert*-Butyl (2-((5-methoxy-4-((4-(1-methyl-1*H*-indol-3-yl)­pyrimidin-2-yl)­amino)-2-nitrophenyl)­(methyl)­amino)­ethyl)­(methyl)­carbamate (**1a**)

*N*-(4-Fluoro-2-methoxy-5-nitrophenyl)-4-(1-methyl-1*H*-indol-3-yl)­pyrimidin-2-amine (2.00 g, 5.08 mmol, 1.0 equiv), *tert*-butyl methyl­(2-(methylamino)­ethyl)­carbamate (1.47 g, 7.63 mmol, 1.5 equiv) and K_2_CO_3_ (1.42 g, 10.17 mmol, 2.0 equiv) were dissolved in 35 mL DMF and heated at 100 °C for 15 h. After cooling to room temperature, the mixture was poured into 80 mL H_2_O, the precipitate filtered off, washed 3× with H_2_O and dried in vacuo at 60 °C. **1a** was obtained as a bright orange solid in 95% yield (2.72 g, 4.84 mmol). ^1^H NMR (500 MHz, DMSO-*d*_6_): δ 8.68 (d, *J* = 21.7 Hz, 1H), 8.37 (d, *J* = 7.8 Hz, 1H), 8.33 (s, 1H), 8.32 (d, *J* = 5.4 Hz, 1H), 8.10 (s, 1H), 7.52 (d, *J* = 8.2 Hz, 1H), 7.25 (t, *J* = 8.0 Hz, 1H), 7.22 (d, *J* = 5.4 Hz, 1H), 7.12 (t, *J* = 7.5 Hz, 1H), 6.83 (d, *J* = 43.1 Hz, 1H), 3.97 (s, 3H), 3.88 (s, 3H), 3.41 (t, *J* = 6.0 Hz, 2H), 3.32–3.26 (m, 2H, overlap with water signal), 2.87 (d, *J* = 5.6 Hz, 3H), 2.77 (d, *J* = 5.6 Hz, 3H), 1.35 (d, *J* = 13.3 Hz, 9H) ppm. MS (*m*/*z*) calcd C_29_H_35_N_7_O_5_: (M + H)^+^, 562.28; found, 562.47.

#### *Tert*-Butyl (2-((2-amino-5-methoxy-4-((4-(1-methyl-1*H*-indol-3-yl)­pyrimidin-2-yl)­amino)­phenyl)­(methyl)­amino)­ethyl)­(methyl)­carbamate (**2a**)

1a (2.63 g, 4.68 mmol, 1.0 equiv) was suspended in 95 mL EtOH and 32 mL H_2_O, then Fe (1.59 g, 28.10 mmol, 6.0 equiv) and NH_4_Cl (0.19 g, 3.51 mmol, 0.75 equiv) were added. The mixture was heated at 100 °C for 18 h and afterward concentrated in vacuo. The residue was taken up in 40 mL DCM, filtered and the filter washed with another 40 mL DCM. The filtrate was concentrated in vacuo to give the crude product as a light-brown foam, which was purified by silica column chromatography using 2.5% MeOH in DCM as the eluent. **2a** was obtained as a light-yellow foam in 91% yield (2.30 g, 4.28 mmol). ^1^H NMR (500 MHz, DMSO-*d*_6_): δ 8.42 (d, *J* = 8.0 Hz, 1H), 8.30 (s, 1H), 8.27 (d, *J* = 5.3 Hz, 1H), 7.78 (s, 1H), 7.55–7.47 (m, 2H), 7.24 (t, *J* = 7.2 Hz, 1H), 7.19–7.13 (m, 2H), 6.77 (s, 1H), 4.41 (s, 2H), 3.88 (s, 3H), 3.75 (s, 3H), 2.94 (t, *J* = 6.6 Hz, 2H), 2.79 (d, *J* = 18.4 Hz, 3H), 2.63 (s, 3H), 1.40 (s, 9H) ppm. 2H below the water signal. MS (*m*/*z*) calcd C_29_H_37_N_7_O_3_: (M + H)^+^, 532.30; found, 532.42.

#### *Tert*-Butyl (2-((2-Acrylamido-5-methoxy-4-((4-(1-methyl-1*H*-indol-3-yl)­pyrimidin-2-yl)­amino)­phenyl)­(methyl)­amino)­ethyl)­(methyl)­carbamate (**3a**)

2a (2.30 g, 4.28 mmol, 1.0 equiv) and DIPEA (1.51 mL, 8.55 mmol, 2.0 equiv) were dissolved in 50 mL dry DCM and cooled to ca. −80 °C using liquid N_2_/acetone. A solution of acryloyl chloride (0.36 mL, 4.28 mmol, 1.0 equiv) in 13 mL dry DCM was added under Ar atmosphere with an automated syringe pump at a flow rate of 40 μL/min. After the addition, the mixture was warmed up very slowly by allowing the cold bath to reach room temperature overnight. The reaction mixture was diluted with DCM to a total volume of 100 mL and washed with 80 mL saturated NaHCO_3_ solution. The organic phase was dried over Na_2_SO_4_ and reduced in vacuo to give the crude product as a yellow foam, which was purified by silica column chromatography using 10% acetone in DCM as the eluent. **3a** was obtained as a pale-yellow foam in 74% yield (1.87 g, 3.16 mmol). ^1^H NMR (500 MHz, DMSO-*d*_6_): δ 9.23–9.04 (m, 1H), 8.96 (s, 1H), 8.61 (s, 1H), 8.32 (d, *J* = 5.3 Hz, 1H), 8.26 (d, *J* = 8.0 Hz, 1H), 7.89 (s, 1H), 7.52 (d, *J* = 8.2 Hz, 1H), 7.27–7.20 (m, 2H), 7.16 (t, *J* = 7.3 Hz, 1H), 6.98 (s, 1H), 6.67 (dd, *J* = 16.7, 10.1 Hz, 1H), 6.26 (d, *J* = 16.9 Hz, 1H), 5.75 (d, *J* = 11.5 Hz, 1H), 3.90 (s, 3H), 3.87 (s, 3H), 2.99 (t, *J* = 6.0 Hz, 2H), 2.82–2.73 (m, 3H), 2.70 (s, 3H), 1.37 (s, 9H) ppm. 2H below the water signal. MS (*m*/*z*) calcd C_32_H_39_N_7_O_4_: (M + H)^+^, 586.31; found, 586.41.

#### *N*-(4-Methoxy-2-(methyl­(2-(methylamino)­ethyl)­amino)-5-((4-(1-methyl-1*H*-indol-3-yl)­pyrimidin-2-yl)­amino)­phenyl)­acrylamide (**OsiNHMe**)

3a (1.87 g, 3.16 mmol, 1.0 equiv) was dissolved in 40 mL DCM and TFA (8.61 mL, 110.63 mmol, 35.0 equiv) was added. The mixture was stirred at room temperature for 1 h and the solvent removed in vacuo. The residue was dissolved in Milli-Q-H_2_O, lyophilized and additionally dried in vacuo at 40 °C, affording the crude product as a bright-yellow, solid TFA salt (2.43 g, including solvent residues). A total of 101 mg were purified by preparative HPLC on an XBridge BEH C18 OBD Prep Column (19 mm × 250 mm) using a mixture of 44% MeOH and 56% Milli-Q water with 0.1% TFA as the eluent. The product fractions were combined, MeOH removed in vacuo and the aqueous phase lyophilized. **OsiNHMe** was obtained in 69% yield (67 mg, 0.090 mmol). ^1^H NMR (500 MHz, DMSO-*d*_6_): δ 9.39 (s, 1H, H22), 9.03 (bs, 1H, H14), 8.70 (s, 1H, H1), 8.67–8.51 (m, 1H, H20 + 2H, H30 due to protonation), 8.39–8.10 (m, 2H, H7 + H12), 7.56 (d, *J* = 8.2 Hz, 1H, H4), 7.33 (d, *J* = 6.1 Hz, 1H, H11), 7.27 (t, *J* = 7.6 Hz, 1H, H5), 7.15 (t, *J* = 7.4 Hz, 1H, H6), 7.04 (s, 1H, H17), 6.72 (dd, *J* = 16.9, 10.2 Hz, 1H, H24), 6.28 (dd, *J* = 17.0, 1.7 Hz, 1H, H25), 5.78 (dd, *J* = 10.3, 1.5 Hz, 1H, H25), 3.91 (s, 3H, H2), 3.85 (s, 3H, H21), 3.25 (t, *J* = 5.7 Hz, 2H, H27), 3.16 (quint, *J* = 5.6 Hz, 2H, H28), 2.66–2.60 (m, 6H, H26 + H29) ppm. ^13^C NMR (126 MHz, DMSO-*d*_6_): δ 164.35 (only in 2D, C10), 163.39 (C23), 158.38 (q, *J* = 32.9 Hz, C_q_-TFA), 156.42 (only in 2D, C13), 151.36 (only in 2D, C12), 148.47 (only in 2D, C16), 140.64 (only in 2D, C18), 137.96 (C3), 136.22 (C1), 132.10 (C24), 126.79 (C25), 125.67 (C19), 125.41 (C8), 122.75 (C5), 122.54 (only in 2D, C15), 121.98 (C7), 121.74 (C6), 118.09 (only in 2D, C20), 116.70 (q, *J* = 296.9 Hz, CF_3_-TFA), 112.02 (C9), 110.90 (C4), 106.60 (C11), 105.23 (C17), 56.11 (C21), 50.73 (C27), 45.57 (C28), 42.53 (C26), 33.37 (C2), 32.46 (C29) ppm. HRMS (*m*/*z*) calcd C_27_H_31_N_7_O_2_: (M + H)^+^, 486.2612; found, 486.2602. Elemental analysis (%) Calcd for C_27_H_31_N_7_O_2_*2TFA*1.5H_2_O: C, 50.27; H, 4.90: N, 13.24. Found: C, 50.38; H, 4.57; N, 13.10.

#### *Tert*-Butyl (2-((5-Methoxy-4-((4-(1-methyl-1*H*-indol-3-yl)­pyrimidin-2-yl)­amino)-2-nitrophenyl)­(methyl)­amino)­ethyl)­carbamate (**1b**)

*N*-(4-Fluoro-2-methoxy-5-nitrophenyl)-4-(1-methyl-1*H*-indol-3-yl)­pyrimidin-2-amine (0.62 g, 1.58 mmol), *tert*-butyl (2-(methylamino)­ethyl)­carbamate (0.42 g, 2.37 mmol) and K_2_CO_3_ (0.44 g, 3.15 mmol) were dissolved in 18 mL DMF and heated at 100 °C for 9 h. After cooling to room temperature, the mixture was poured into 100 mL H_2_O, the precipitate filtered off and dried in vacuo at 40 °C. **1b** was obtained as a red solid in quantitative yield (0.88 g, 1.58 mmol). ^1^H NMR (500 MHz, DMSO-*d*_6_): δ 8.66 (s, 1H), 8.36 (d, *J* = 7.6 Hz, 1H), 8.33 (s, 1H), 8.31 (d, *J* = 5.4 Hz, 1H), 8.09 (s, 1H), 7.52 (d, *J* = 8.2 Hz, 1H), 7.24 (t, *J* = 7.7 Hz, 1H), 7.21 (d, *J* = 5.4 Hz, 1H), 7.12 (t, *J* = 7.4 Hz, 1H), 6.86 (s, 1H), 6.84 (t, *J* = 5.2 Hz, 1H), 3.97 (s, 3H), 3.88 (s, 3H), 3.25–3.15 (m, 4H), 2.84 (s, 3H), 1.33 (s, 9H) ppm. MS (*m*/*z*) calcd C_28_H_33_N_7_O_5_: (M + H)^+^, 548.26; found, 548.30.

#### *Tert*-Butyl (2-((2-Amino-5-methoxy-4-((4-(1-methyl-1*H*-indol-3-yl)­pyrimidin-2-yl)­amino)­phenyl)­(methyl)­amino)­ethyl)­carbamate (**2b**)

1b (0.87 g, 1.56 mmol) was suspended in 33 mL EtOH and 11 mL H_2_O, then Fe (0.53 g, 9.35 mmol) and NH_4_Cl (0.063 g, 1.17 mmol) were added. The mixture was heated at 100 °C for 3.5 h and afterward concentrated in vacuo. The residue was taken up in 60 mL DCM containing 10% MeOH, filtered and the filter washed with another 60 mL DCM containing 10% MeOH. The filtrate was washed 2× with 60 mL brine, dried over Na_2_SO_4_ and concentrated in vacuo to give the crude product as a light-brown foam, which was purified by silica column chromatography using 2% MeOH in DCM as the eluent. **2b** was obtained as a yellow foam in 92% yield (0.76 g, 1.43 mmol). ^1^H NMR (500 MHz, DMSO-*d*_6_): δ 8.42 (d, *J* = 8.0 Hz, 1H), 8.31 (s, 1H), 8.26 (d, *J* = 5.3 Hz, 1H), 7.79 (s, 1H), 7.51 (d, *J* = 8.2 Hz, 1H), 7.49 (s, 1H), 7.24 (t, *J* = 7.6 Hz, 1H), 7.19–7.13 (m, 2H), 6.89 (t, *J* = 5.7 Hz, 1H), 6.74 (s, 1H), 4.50 (s, 2H), 3.88 (s, 3H), 3.74 (s, 3H), 3.08 (q, *J* = 6.3 Hz, 2H), 2.85 (t, *J* = 6.6 Hz, 2H), 2.58 (s, 3H), 1.38 (s, 9H) ppm. MS (*m*/*z*) calcd C_28_H_35_N_7_O_3_: (M + H)^+^, 518.29; found, 518.32.

#### *Tert*-Butyl (2-((2-Acrylamido-5-methoxy-4-((4-(1-methyl-1*H*-indol-3-yl)­pyrimidin-2-yl)­amino)­phenyl)­(methyl)­amino)­ethyl)­carbamate (**3b**)

2b (0.69 g, 1.29 mmol) and DIPEA (0.46 mL, 2.59 mmol) were dissolved in 15 mL dry DCM and cooled to ca. −80 °C using liquid N_2_/acetone. A solution of acryloyl chloride (0.11 mL, 1.29 mmol) in 4.2 mL dry DCM was added under Ar atmosphere with an automated syringe pump at a flow rate of 40 μL/min. After the addition, the mixture was warmed up very slowly by allowing the cold bath to reach −15 °C. Stirring was then continued in an ice bath for 10 min, then at room temperature for 40 min. The reaction mixture was diluted with 70 mL DCM containing 10% MeOH and washed 3× with 70 mL saturated NaHCO_3_ solution. The organic phase was dried over Na_2_SO_4_ and reduced in vacuo to give the crude product as a yellow foam, which was purified by silica column chromatography using 10% acetone in DCM as the eluent. **3b** was obtained as a yellow foam in 87% yield (0.68 g, 1.13 mmol). ^1^H NMR (500 MHz, DMSO-*d*_6_): δ 9.23 (s, 1H), 9.02 (s, 1H), 8.63 (s, 1H), 8.32 (d, *J* = 5.3 Hz, 1H), 8.25 (d, *J* = 7.9 Hz, 1H), 7.91 (s, 1H), 7.52 (d, *J* = 8.2 Hz, 1H), 7.26–7.21 (m, 2H), 7.15 (t, *J* = 7.5 Hz, 1H), 7.04 (t, *J* = 5.3 Hz, 1H), 6.98 (s, 1H), 6.72 (dd, *J* = 16.9, 10.2 Hz, 1H), 6.27 (dd, *J* = 16.8, 1.1 Hz, 1H), 5.75 (dd, *J* = 10.5, 1.6 Hz, 1H), 3.91 (s, 3H), 3.86 (s, 3H), 3.10 (q, *J* = 6.0 Hz, 2H), 2.90 (t, *J* = 6.2 Hz, 2H), 2.64 (s, 3H), 1.37 (s, 9H) ppm. MS (*m*/*z*) calcd C_31_H_37_N_7_O_4_: (M + H)^+^, 572.30; found, 572.35.

#### *N*-(2-((2-Aminoethyl)­(methyl)­amino)-4-methoxy-5-((4-(1-methyl-1*H*-indol-3-yl)­pyrimidin-2-yl)­amino)­phenyl)­acrylamide (**OsiNH**_**2**_)

3b (0.67 g, 1.11 mmol) was dissolved in 20 mL DCM and TFA (4.55 mL, 58.42 mmol) was added. The mixture was stirred at room temperature for 40 min and the solvent removed in vacuo. The residue was dissolved in Milli-Q water, lyophilized and additionally dried in vacuo at 40 °C, affording the crude product as a bright-yellow, solid TFA salt (0.83 g, including solvent residues). A total of 100 mg were purified by preparative HPLC on an XBridge BEH C18 OBD Prep Column (19 mm × 250 mm) using a mixture of 44% MeOH and 56% Milli-Q water with 0.1% TFA as the eluent. The product fractions were combined, MeOH removed in vacuo and the aqueous phase lyophilized. **OsiNH**_**2**_ was obtained in 75% yield (73 mg, 0.10 mmol). ^1^H NMR (500 MHz, DMSO-*d*_6_): δ 9.34 (s, 1H, H22), 9.04 (bs, 1H, H14), 8.70 (s, 1H, H1), 8.62 (s, 1H, H20), 8.38–8.10 (m, 2H, H7 + H12), 8.01–7.80 (m, 3H, H29 due to protonation), 7.56 (d, *J* = 8.2 Hz, 1H, H4), 7.33 (d, *J* = 6.1 Hz, 1H, H11), 7.27 (t, *J* = 7.6 Hz, 1H, H5), 7.16 (t, *J* = 7.4 Hz, 1H, H6), 7.02 (s, 1H, H17), 6.72 (dd, *J* = 17.0, 10.2 Hz, 1H, H24), 6.26 (dd, *J* = 17.0, 1.7 Hz, 1H, H25), 5.78 (dd, *J* = 10.0, 1.5 Hz, 1H, H25), 3.91 (s, 3H, H2), 3.84 (s, 3H, H21), 3.21 (t, *J* = 5.7 Hz, 2H, H27), 3.10–3.01 (m, 2H, H28), 2.61 (s, 3H, H26) ppm. ^13^C NMR (126 MHz, DMSO-*d*_6_): δ 164.34 (only in 2D, C10), 163.27 (C23), 158.31 (q, *J* = 32.9 Hz, C_q_-TFA), 156.15 (only in 2D, C13), 151.65 (only in 2D, C12), 148.48 (only in 2D, C16), 140.84 (only in 2D, C18), 137.96 (C3), 136.31 (C1), 132.18 (C24), 126.67 (C25), 125.70 (C19), 125.41 (C8), 122.77 (C5), 122.68 (only in 2D, C15), 121.99 (C7), 121.79 (C6), 118.02 (only in 2D, C20), 116.72 (q, *J* = 297.0 Hz, CF_3_-TFA), 112.01 (C9), 110.90 (C4), 106.55 (C11), 105.10 (C17), 56.10 (C21), 52.13 (C27), 42.36 (C26), 36.41 (C28), 33.38 (C2) ppm. HRMS (*m*/*z*) calcd C_26_H_29_N_7_O_2_: (M + H)^+^, 472.2455; found, 472.2450. Elemental analysis (%) Calcd for C_26_H_29_N_7_O_2_*2TFA*1.5H_2_O: C, 49.59; H, 4.72; N, 13.49. Found: C, 49.66; H, 4.35; N, 13.30.

#### *Tert*-Butyl Methyl­(3-(methylamino)­propyl)­carbamate

*N*,*N*′-Dimethylpropane-1,3-diamine (1.80 mL, 14.33 mmol) was dissolved in 40 mL dry DCM and cooled to 0 °C. A solution of di-*tert*-butyl dicarbonate (1.07 g, 4.81 mmol) in 20 mL dry DCM was added under Ar atmosphere with an automated syringe pump at a flow rate of 50 μL/min. The mixture was warmed to room temperature and stirred for 45 min. Then, the mixture was washed with 70 mL Milli-Q water and the aqueous phase extracted 3× with EtOAc. The combined organic phases were washed 2× with brine, dried over MgSO_4_ and concentrated in vacuo. *Tert*-butyl methyl­(3-(methylamino)­propyl)­carbamate was obtained as a clear oil in 89% yield (0.94 g containing 7% EtOAc residue, 4.30 mmol). ^1^H NMR (500 MHz, DMSO-*d*_6_): δ 3.17 (t, *J* = 7.1 Hz, 2H), 3.15–3.07 (m, 1H), 2.75 (bs, 3H), 2.40 (t, *J* = 6.7 Hz, 2H), 2.25 (s, 3H), 1.57 (quint, *J* = 6.8 Hz, 2H), 1.38 (s, 9H) ppm. MS (*m*/*z*) calcd C_10_H_22_N_2_O_2_: (M + H)^+^, 203.18; found, 203.12.

#### *Tert*-Butyl (3-((5-Methoxy-4-((4-(1-methyl-1*H*-indol-3-yl)­pyrimidin-2-yl)­amino)-2-nitrophenyl)­(methyl)­amino)­propyl)­(methyl)­carbamate (**1c**)

*N*-(4-Fluoro-2-methoxy-5-nitrophenyl)-4-(1-methyl-1*H*-indol-3-yl)­pyrimidin-2-amine (0.50 g, 1.27 mmol), OsiPropNHMe-1 (0.42 mg, 1.91 mmol) and K_2_CO_3_ (0.36 g, 2.54 mmol) were dissolved in 8 mL DMF and heated at 100 °C for 9 h. After cooling to room temperature, the mixture was poured into 80 mL H_2_O, the precipitate filtered off and dried in vacuo at 40 °C. **1c** was obtained as a bright orange solid in 95% yield (0.69 g, 1.21 mmol). ^1^H NMR (500 MHz, DMSO-*d*_6_): δ 8.66 (s, 1H), 8.42–8.28 (m, 3H), 8.10 (s, 1H), 7.52 (d, *J* = 8.2 Hz, 1H), 7.24 (t, *J* = 7.8 Hz, 1H), 7.22 (d, *J* = 5.4 Hz, 1H), 7.11 (t, *J* = 7.6 Hz, 1H), 6.80 (s, 1H), 3.96 (s, 3H), 3.88 (s, 3H), 3.22–3.09 (m, 4H), 2.82 (s, 3H), 2.75 (s, 3H), 1.78 (quint, *J* = 6.9 Hz, 2H), 1.37 (s, 9H) ppm. MS (*m*/*z*) calcd C_30_H_37_N_7_O_5_: (M + Na)^+^, 598.27; found, 598.33.

#### *Tert*-Butyl (3-((2-Amino-5-methoxy-4-((4-(1-methyl-1*H*-indol-3-yl)­pyrimidin-2-yl)­amino)­phenyl)­(methyl)­amino)­propyl)­(methyl)­carbamate (**2c**)

1c (0.69 g, 1.20 mmol) was suspended in 25 mL EtOH and 8.4 mL H_2_O, then Fe (0.41 g, 7.21 mmol) and NH_4_Cl (0.049 g, 0.90 mmol) were added. The mixture was heated at 100 °C for 5 h and afterward concentrated in vacuo. The residue was taken up in 60 mL DCM containing 10% MeOH, filtered and the filter washed with another 60 mL DCM containing 10% MeOH. The filtrate was washed with 60 mL brine, dried over MgSO_4_ and concentrated in vacuo to give the crude product as a light-brown foam, which was purified by silica column chromatography using a 1–2% MeOH gradient in DCM as the eluent. **2c** was obtained as a yellow foam in 89% yield (0.59 g, 1.07 mmol). ^1^H NMR (500 MHz, DMSO-*d*_6_): δ 8.42 (d, *J* = 8.0 Hz, 1H), 8.30 (s, 1H), 8.27 (d, *J* = 5.3 Hz, 1H), 7.78 (s, 1H), 7.52 (s, 1H), 7.51 (d, *J* = 8.6 Hz, 1H), 7.27–7.22 (m, 1H), 7.18–7.13 (m, 2H), 6.74 (s, 1H), 4.45 (s, 2H), 3.88 (s, 3H), 3.74 (s, 3H), 3.22 (t, *J* = 7.1 Hz, 2H), 2.82 (t, *J* = 5.9 Hz, 2H), 2.75 (bs, 3H), 2.57 (s, 3H), 1.65 (bs, 2H), 1.38 (s, 9H) ppm. MS (*m*/*z*) calcd C_30_H_39_N_7_O_3_: (M + H)^+^, 546.32; found, 546.36.

#### *Tert*-Butyl (3-((2-Acrylamido-5-methoxy-4-((4-(1-methyl-1*H*-indol-3-yl)­pyrimidin-2-yl)­amino)­phenyl)­(methyl)­amino)­propyl)­(methyl)­carbamate (**3c**)

2c (0.30 g, 0.54 mmol) and DIPEA (0.19 mL, 1.070 mmol) were dissolved in 6 mL dry DCM and cooled to ca. −80 °C using liquid N_2_/acetone. A solution of acryloyl chloride (0.047 mL, 0.56 mmol) in 1.8 mL dry DCM was added under Ar atmosphere with an automated syringe pump at a flow rate of 40 μL/min. After the addition, the mixture was stirred at −70 °C for 10 min, then stirring was then continued at room temperature for 45 min. The reaction mixture was diluted with 25 mL of DCM containing 10% MeOH and washed 3× with 25 mL saturated NaHCO_3_ solution. The organic phase was dried over Na_2_SO_4_ and reduced in vacuo to give the crude product as a yellow foam, which was purified by silica column chromatography using a 6–10% acetone gradient in DCM as the eluent. **3c** was obtained as a yellow foam in 84% yield (0.27 g, 0.45 mmol). ^1^H NMR (500 MHz, DMSO-*d*_6_): δ 9.51–9.11 (m, 1H), 9.03–8.85 (m, 1H), 8.65–8.55 (m, 1H), 8.32 (d, *J* = 5.3 Hz, 1H), 8.25 (d, *J* = 7.9 Hz, 1H), 7.89 (s, 1H), 7.52 (d, *J* = 8.2, 1H), 7.26–7.21 (m, 2H), 7.15 (t, *J* = 7.3 Hz, 1H), 6.93 (s, 1H), 6.87–6.64 (m, 1H), 6.26 (d, *J* = 17.0 Hz, 1H), 5.73 (d, *J* = 10.6 Hz, 1H), 3.90 (s, 3H), 3.86 (s, 3H), 3.21 (bs, 2H), 2.85 (t, *J* = 6.5 Hz, 2H), 2.78–2.70 (m, 3H), 2.67–2.55 (m, 3H), 1.64 (bs, 2H), 1.43–1.34 (m, 9H) ppm. MS (*m*/*z*) calcd C_33_H_41_N_7_O_4_: (M + H)^+^, 600.33; found, 600.37.

#### *N*-(4-Methoxy-2-(methyl­(3-(methylamino)­propyl)­amino)-5-((4-(1-methyl-1*H*-indol-3-yl)­pyrimidin-2-yl)­amino)­phenyl)­acrylamide (**OsiPropNHMe**)

3c (0.27 g, 0.45 mmol) was dissolved in 8 mL DCM and TFA (1.22 mL, 15.70 mmol) was added. The mixture was stirred at room temperature for 35 min and the solvent removed in vacuo. The residue was dissolved in Milli-Q water, lyophilized and additionally dried in vacuo at 40 °C, affording the crude product as a bright-yellow, solid TFA salt (0.36 g, including solvent residues). A total of 170 mg were purified by preparative HPLC on an XBridge BEH C18 OBD Prep Column (19 mm × 250 mm) using a mixture of 45% MeOH and 55% Milli-Q water with 0.1% TFA as the eluent. The product fractions were combined, MeOH removed in vacuo and the aqueous phase lyophilized. **OsiPropNHMe** was obtained in 64% yield (116 mg, 0.14 mmol). ^1^H NMR (500 MHz, DMSO-*d*_6_): δ 10.06–8.83 (m, 2H, H14 + H22), 8.74 (s, 1H, H1), 8.66–8.35 (m, 1H, H20 + 2H, H30 due to protonation), 8.35–8.06 (m, 2H, H7 + H12), 7.57 (d, *J* = 8.2 Hz, 1H, H4), 7.35 (d, *J* = 6.3 Hz, 1H, H11), 7.29 (t, *J* = 7.6 Hz, 1H, H5), 7.17 (t, *J* = 7.3 Hz, 1H, H6), 7.01 (s, 1H, H17), 6.72 (dd, *J* = 16.9, 10.2 Hz, 1H, H24), 6.23 (dd, *J* = 17.0, 1.6 Hz, 1H, H25), 5.74 (dd, *J* = 10.3, 1.4 Hz, 1H, H25), 3.92 (s, 3H, H2), 3.84 (s, 3H, H21), 3.00 (t, *J* = 6.2 Hz, 2H, H27), 2.97–2.89 (m, 2H, H29), 2.70 (s, 3H, H26), 2.54 (t, *J* = 5.4 Hz, 3H, H31), 1.79 (quint, *J* = 7.3 Hz, 2H, H28) ppm. ^13^C NMR (126 MHz, DMSO-*d*_6_): δ 165.11 (only in 2D, C10), 163.23 (C23), 158.36 (q, *J* = 33.4 Hz, C_q_-TFA), 155.16 (only in 2D, C13), 149.20 (only in 2D, C12), 148.85 (only in 2D, C16), 141.40 (only in 2D, C18), 138.04 (C3), 136.91 (C1), 132.24 (C24), 126.53 (C25), 125.60 (C19), 125.41 (C8), 122.94 (C5), 121.99 (C6 + C7 and only in 2D, C15), 118.70 (only in 2D, C20), 116.53 (q, *J* = 296.1 Hz, CF_3_-TFA), 111.91 (C9), 111.00 (C4), 106.38 (C11), 104.95 (C17), 56.04 (C21), 52.53 (C27), 46.43 (C29), 42.03 (C26), 33.45 (C2), 32.59 (C31), 23.34 (C28) ppm. HRMS (*m*/*z*) calcd C_28_H_33_N_7_O_2_: (M + H)^+^, 500.2768; found, 500.2765. Elemental analysis (%) Calcd for C_28_H_33_N_7_O_2_*3TFA*H_2_O: C, 47.50; H, 4.46; N, 11.40. Found: C, 47.61; H, 4.42; N, 11.47.

#### (9*H*-Fluoren-9-yl)­methyl (2-((2-((4-(hydroxymethyl)­phenyl)­amino)-2-oxoethyl)­amino)-2-oxoethyl)­carbamate (**Fmoc-GlyGly-PAB-OH**)

(((9*H*-Fluoren-9-yl)­methoxy)­carbonyl)­glycylglycine (1.00 g, 2.77 mmol, 1.0 equiv) was suspended in 30 mL DCM and 4-aminobenzyl alcohol (0.70 g, 5.53 mmol, 2.0 equiv) was added. 10 mL MeOH were added and the mixture sonicated until a clear light-brown solution was obtained. EEDQ (1.38 g, 5.53 mmol, 2.0 equiv) was added and the mixture stirred for 19 h, whereby a white solid precipitated. The solid was filtered off, washed with a mixture of 25% MeOH in DCM and dried in vacuo. Fmoc-GlyGly-PAB-OH was obtained as a white solid in 87% yield (1.20 g containing 8% DCM residue, 2.41 mmol). ^1^H NMR (500 MHz, DMSO-*d*_6_): δ 9.81 (s, 1H), 8.22 (t, *J* = 5.6 Hz, 1H), 7.89 (d, *J* = 7.5 Hz, 2H), 7.72 (d, *J* = 7.5 Hz, 2H), 7.64 (t, *J* = 6.0 Hz, 1H), 7.54 (d, *J* = 8.4 Hz, 2H), 7.42 (t, *J* = 7.4 Hz, 2H), 7.33 (t, *J* = 7.2 Hz, 2H), 7.24 (d, *J* = 8.4 Hz, 2H), 5.09 (t, *J* = 5.7 Hz, 1H), 4.43 (d, *J* = 5.7 Hz, 2H), 4.30 (d, *J* = 7.0 Hz, 2H), 4.24 (t, *J* = 6.9 Hz, 1H), 3.89 (d, *J* = 5.7 Hz, 2H), 3.68 (d, *J* = 6.0 Hz, 2H) ppm. MS (*m*/*z*) calcd C_26_H_25_N_3_O_5_: (M + Na)^+^, 482.17; found, 482.17.

#### 2-Amino-*N*-(2-((4-(hydroxymethyl)­phenyl)­amino)-2-oxoethyl)­acetamide (**H**_**2**_ **N-GlyGly-PAB-OH**)

Fmoc-GlyGly-PAB-OH (1.19 g, 2.38 mmol, 1.0 equiv) was dissolved in 20 mL DMF and piperidine (8 mL, 79.99 mmol, 33.6 equiv) was added. The mixture was stirred for 1 h at room temperature, then all volatiles were removed in vacuo. The residue was triturated in 40 mL DCM and the product filtered off and washed with DCM. H_2_N-GlyGly-PAB-OH was obtained as a white solid in 80% yield (0.45 g, 1.91 mmol). ^1^H NMR (500 MHz, DMSO-*d*_6_): δ 9.92 (s, 1H), 8.20 (s, 1H), 7.53 (d, *J* = 8.2 Hz, 2H), 7.25 (d, *J* = 8.2 Hz, 2H), 5.10 (s, 1H), 4.44 (s, 2H), 3.93 (s, 2H), 3.16 (s, 2H), 2.04 (s, 2H) ppm. MS (*m*/*z*) calcd C_11_H_15_N_3_O_3_: (M + H)^+^, 260.10; found, 260.10.

#### 3-(4-(2-(2-(4,7-Dimethyl-1,3-dioxo-1,3,3*a*,4,7,7*a*-hexahydro-2*H*-4,7-epoxyisoindol-2-yl)­ethoxy)­ethyl)­piperazin-1-yl)-*N*-(2-((2-((4-(hydroxymethyl)­phenyl)­amino)-2-oxoethyl)­amino)-2-oxoethyl)­propenamide (**pMal-O-Pip-GlyGly-PAB-OH**)

pMal-O-Pip-COOH·2HCl (333 mg, 0.67 mmol, 1.0 equiv) in a dry flask was dissolved in 13 mL dry DMF at room temperature under Ar atmosphere and NEt_3_ (0.94 mL, 6.74 mmol, 10.0 equiv) and EDC·HCl (198 mg, 1.01 mmol, 1.5 equiv) were added. The mixture was stirred for 10 min, then HOBt·H_2_O (158 mg, 1.01 mmol, 1.5 equiv) was added and stirring was continued for another 10 min. Finally, H_2_N-GlyGly-PAB-OH (240 mg, 1.01 mmol, 1.5 equiv) was added and the mixture was stirred for 17 h. All volatiles were removed in vacuo and the residue purified by preparative HPLC on an XBridge BEH C18 OBD Prep Column (19 mm × 250 mm) using a mixture of 13% CH_3_CN and 87% Milli-Q water with 0.1% TFA as the eluent. The product fractions were combined, CH_3_CN removed in vacuo and the aqueous phase lyophilized twice. pMal-O-Pip-GlyGly-PAB-OH was obtained as a white solid TFA salt in 76% yield (444 mg, 0.51 mmol). ^1^H NMR (500 MHz, DMSO-*d*_6_): δ 9.84 (s, 1H), 8.47–8.36 (m, 1H), 8.26 (t, *J* = 5.0 Hz, 1H), 7.54 (d, *J* = 8.4 Hz, 2H), 7.24 (d, *J* = 8.5 Hz, 2H), 6.38 (s, 2H), 4.43 (s, 2H), 3.90 (d, *J* = 5.8 Hz, 2H), 3.79 (d, *J* = 5.5 Hz, 2H), 2.90 (s, 2H), 1.54 (s, 6H) ppm. The remaining 20 aliphatic H-signals of the pMal-O-Pip linker and the alcohol signal overlap with the broad water peak (∼3.70–2.70 ppm). MS (*m*/*z*) calcd C_32_H_44_N_6_O_8_: (M + H)^+^, 641.33; found, 641.30.

#### 4-(2-(2-(3-(4-(2-(2-(4,7-Dimethyl-1,3-dioxo-1,3,3*a*,4,7,7*a*-hexahydro-2*H*-4,7-epoxyisoindol-2-yl)­ethoxy)­ethyl)­piperazin-1-yl)­propanamido)­acetamido)­acetamido)­benzyl (4-nitrophenyl) carbonate (**pMal-O-Pip-GlyGly-PAB-PNP**)

pMal-O-Pip-GlyGly-PAB-OH (250 mg, 0.29 mmol, 1.0 equiv) in a dry flask was dissolved in 10 mL dry DMF at room temperature under Ar atmosphere and bis­(4-nitrophenyl) carbonate (268 mg, 0.86 mmol, 3.0 equiv) and DIPEA (0.76 mL, 4.32 mmol, 15.0 equiv) were added. The mixture was stirred for 7 h, then all volatiles were removed in vacuo. The residue was purified by silica column chromatography using a gradient of 10–30% MeOH in DCM as the eluent. The product-containing fractions were combined and 30 mL toluene were added before concentrating them in vacuo. pMal-O-Pip-GlyGly-PAB-PNP was obtained as an off-white solid in 97% yield (262 mg containing 9% toluene and 5% DIPEA·TFA salt, 0.28 mmol). ^1^H NMR (500 MHz, DMSO-*d*_6_): δ 9.94 (bs, 1H), 8.51–8.34 (m, 1H), 8.34–8.28 (m, 2H), 8.22 (bs, 1H), 7.66 (d, *J* = 8.2 Hz, 2H), 7.60–7.53 (m, 2H), 7.42 (d, *J* = 8.6 Hz, 2H), 6.37 (s, 2H), 5.25 (s, 2H), 3.91 (d, *J* = 5.8 Hz, 2H), 3.76 (s, 2H), 3.57–3.43 (m, 6H), 3.07–2.81 (m, 2H, overlap with C*H*_exo_ signal), 2.89 (s, 2H), 1.53 (s, 6H) ppm. The remaining 12 aliphatic H-signals of the pMal-O-Pip linker overlap with the DMSO peak (∼2.70–2.20 ppm). MS (*m*/*z*) calcd C_39_H_47_N_7_O_12_: (M + H)^+^, 806.34; found, 806.31.

#### 4-(2-(2-(3-(4-(2-(2-(4,7-Dimethyl-1,3-dioxo-1,3,3*a*,4,7,7*a*-hexahydro-2*H*-4,7-epoxyisoindol-2-yl)­ethoxy)­ethyl)­piperazin-1-yl)­propanamido)­acetamido)­acetamido)­benzyl (2-((2-acrylamido-5-methoxy-4-((4-(1-methyl-1*H*-indol-3-yl)­pyrimidin-2-yl)­amino)­phenyl)­(methyl)­amino)­ethyl)­(methyl)­carbamate (**pMal-O-Pip-GlyGly-PAB-OsiNHMe**)

pMal-O-Pip-GlyGly-PAB-PNP (260 mg, 0.28 mmol, 1.0 equiv) and OsiNHMe (293 mg, 0.38 mmol, 1.4 equiv) in a dry flask were dissolved in 10 mL dry DMF at room temperature under Ar atmosphere and DIPEA (0.26 mL, 1.47 mmol, 5.3 equiv) was added. The mixture was stirred for 4 h, then all volatiles were removed in vacuo. The residue was purified by silica column chromatography using a gradient of 10–25% MeOH in DCM as the eluent. pMal-O-Pip-GlyGly-PAB-OsiNHMe was obtained as a pale-yellow solid in 85% yield (307 mg containing 6% MeOH, 0.25 mmol). ^1^H NMR (500 MHz, DMSO-*d*_6_): δ 9.81 (s, 1H), 9.11 (d, *J* = 24.5 Hz, 1H), 8.95 (s, 1H), 8.61 (s, 1H), 8.48–8.36 (m, 1H), 8.32 (d, *J* = 5.3 Hz, 1H), 8.26 (d, *J* = 7.8 Hz, 1H), 8.20–8.12 (m, 1H), 7.87 (s, 1H), 7.65–7.55 (m, 2H), 7.52 (d, *J* = 8.2 Hz, 1H), 7.33–7.26 (m, 2H), 7.26–7.20 (m, 2H), 7.16 (t, *J* = 7.3 Hz, 1H), 6.94 (d, *J* = 22.9 Hz, 1H), 6.64 (dd, *J* = 16.8, 10.3 Hz, 1H), 6.35 (s, 2H), 6.24 (d, *J* = 17.7 Hz, 1H), 5.76–5.66 (m, 1H), 4.99 (s, 2H), 3.90 (s, 3H), 3.90–3.88 (m, 2H), 3.85 (d, *J* = 17.0 Hz, 3H), 3.74 (d, *J* = 5.5 Hz, 2H), 3.49 (t, *J* = 5.6 Hz, 2H), 3.46–3.37 (m, 6H), 3.08–2.98 (m, 2H), 2.87 (s, 2H), 2.84–2.78 (m, 3H), 2.71–2.60 (m, 3H, overlap with DMSO ^13^C satellite signal), 1.52 (s, 6H) ppm. The remaining 14 aliphatic H-signals of the pMal-O-Pip linker overlap with the DMSO peak (∼2.60–2.10 ppm). MS (*m*/*z*) calcd C_60_H_73_N_13_O_11_: (M + H)^+^, 1152.56; found, 1152.53.

#### 4-(2-(2-(3-(4-(2-(2-(2,5-Dioxo-2,5-dihydro-1*H*-pyrrol-1-yl)­ethoxy)­ethyl)­piperazin-1-yl)­propanamido)­acetamido)­acetamido)­benzyl (2-((2-Acrylamido-5-methoxy-4-((4-(1-methyl-1*H*-indol-3-yl)­pyrimidin-2-yl)­amino)­phenyl)­(methyl)­amino)­ethyl)­(methyl)­carbamate (**Mal-Pip-GlyGly**)

pMal-O-Pip-GlyGly-PAB-OsiNHMe (293 mg, 0.24 mmol) was dissolved in 6 mL DMSO and stirred at 90 °C for 3 h. All volatiles were removed in vacuo and the residue was purified by preparative HPLC on an XBridge BEH C18 OBD Prep Column (19 mm × 250 mm) using a mixture of 30% CH_3_CN and 70% Milli-Q water with 0.1% TFA as the eluent. The product fractions were combined, CH_3_CN removed in vacuo and the aqueous phase lyophilized twice. Mal-Pip-GlyGly was obtained as a yellow solid in 64% yield (221 mg, 0.15 mmol). ^1^H NMR (500 MHz, DMSO-*d*_6_): δ 9.98 (s, 1H, H36), 9.55 (bs, 1H, H14 or H22), 9.22 (d, *J* = 17.7 Hz, 1H, H14 or H22), 8.76 (s, 1H, H1), 8.48 (t, *J* = 5.5 Hz, 1H, H42), 8.44 (bs, 1H, H20), 8.31 (t, *J* = 5.6 Hz, 1H, H39), 8.29–8.03 (m, 2H, H7 + H12), 7.67–7.49 (m, 3H, H4 + H34), 7.37 (d, *J* = 6.4 Hz, 1H, H11), 7.33–7.23 (m, 3H, H5 + H33), 7.17 (t, *J* = 6.9 Hz, 1H, H6), 7.04 (s, 2H, H49), 7.03–6.94 (m, 1H, H17), 6.65 (dd, *J* = 16.8, 10.3 Hz, 1H, H24), 6.21 (d, *J* = 17.1 Hz, 1H, H25), 5.80–5.60 (m, 1H, H25), 4.99 (s, 2H, H31), 3.92 (s, 3H, H2), 3.90 (d, *J* = 5.8 Hz, 2H, H38), 3.86–3.76 (m, 5H, H21 + H41), 3.66 (t, *J* = 4.5 Hz, 2H, H45), 3.59 (t, *J* = 5.3 Hz, 2H, H47), 3.53 (t, *J* = 5.2 Hz, 2H, H46), 3.50–2.86 (m, 16H, H_Pip‑linker_ + H27 + H28), 2.81 (d, *J* = 16.0 Hz, 3H, H29), 2.74 (d, *J* = 26.8 Hz, 3H, H26), 2.59 (t, *J* = 6.3 Hz, 2H, H44) ppm. ^13^C NMR (126 MHz, DMSO-*d*_6_): δ 171.05 (C48), 169.94 (C43), 169.26 (C40), 167.66 (C37), 165.59 (only in 2D, C10), 163.05 (C23), 158.45 (q, *J* = 33.8 Hz, C_q_-TFA), 155.69 + 155.45 (C30 + C30′), 149.15 (only in 2D, C16), 147.70 (only in 2D, C12), 141.93 (only in 2D, C18), 138.51 (C3 or C35), 138.08 (C3 or C35), 137.46 (C1), 134.63 (C49), 132.10 (C24), 131.71 (C32), 128.42 (C33), 126.55 (C25), 125.42 (C8), 125.12 + 125.03 (C19 + C19′), 123.06 (C5), 122.14 (C6 + C7), 119.05 (C20 + C34), 116.47 (q, *J* = 295.6 Hz, CF_3_-TFA), 111.84 (C9), 111.04 (C4), 106.17 (C11), 104.90 + 104.67 (C17 + C17′), 67.34 (C46), 66.06 (C31), 64.99 (C45), 55.93 (C21), 55.07 (C_Pip‑linker_), 53.28 + 52.78 (C27 + C27′), 51.95 (C_Pip‑linker_), 46.55 + 45.89 (C28 + C28′), 42.59 (C38), 42.09 (C41), 41.83 + 41.50 (C26 + C26′), 36.59 (C47), 34.46 + 34.00 (C29 + C29′), 33.52 (C2), 30.29 (C44) ppm. C13, C15 and four C_Pip‑linker_ signals could not be observed. HRMS (*m*/*z*) calcd C_54_H_65_N_13_O_10_: (M + H)^+^, 1056.5050; found, 1056.5031. Elemental analysis (%) Calcd for C_54_H_65_N_13_O_10_*3.5TFA: C, 50.35; H, 4.74; N, 12.51. Found: C, 50.25; H, 4.71; N, 12.30.

#### (2*S*)-2-((2*S*)-2-(3-(4-(2-(2-(4,7-Dimethyl-1,3-dioxo-1,3,3*a*,4,7,7*a*-hexahydro-2*H*-4,7-epoxyisoindol-2-yl)­ethoxy)­ethyl)­piperazin-1-yl)­propanamido)-3-methylbutanamido)-*N*-(4-(hydroxymethyl)­phenyl)-5-ureidopentanamide (**pMal-O-Pip-ValCit-PAB-OH**)

pMal-O-Pip-COOH·2HCl (333 mg, 0.67 mmol, 1.0 equiv) in a dry flask was dissolved in 13 mL dry DMF at room temperature under Ar atmosphere and NEt_3_ (0.94 mL, 6.74 mmol, 10.0 equiv) and EDC·HCl (198 mg, 1.01 mmol, 1.5 equiv) were added. The mixture was stirred for 10 min, then HOBt·H_2_O (158 mg, 1.01 mmol, 1.5 equiv) was added and stirring was continued for another 10 min. Finally, H_2_N-ValCit-PAB-OH (388 mg, 1.01 mmol, 1.5 equiv) was added and the mixture was stirred for 17 h. All volatiles were removed in vacuo and the residue purified by preparative HPLC on an XBridge BEH C18 OBD Prep Column (19 mm × 250 mm) using a mixture of 16% CH_3_CN and 84% Milli-Q water with 0.1% TFA as the eluent. The product fractions were combined, CH_3_CN removed in vacuo and the aqueous phase lyophilized twice. pMal-O-Pip-ValCit-PAB-OH was obtained as a white solid TFA salt in 66% yield (452 mg, 0.45 mmol). ^1^H NMR (500 MHz, DMSO-*d*_6_): δ 9.93 (s, 1H), 8.26–7.99 (m, 2H), 7.53 (d, *J* = 7.8 Hz, 2H), 7.23 (d, *J* = 7.4 Hz, 2H), 6.38 (s, 2H), 6.01 (s, 1H), 5.43 (bs, 2H), 4.48–4.35 (m, 3H), 4.26 (t, *J* = 7.0 Hz, 1H), 2.91 (s, 2H), 2.04–1.94 (m, 1H), 1.74–1.49 (m, 8H), 1.48–1.30 (m, 2H), 0.87 (d, *J* = 6.4 Hz, 3H), 0.84 (d, *J* = 6.3 Hz, 3H) ppm. The remaining 20 aliphatic H-signals of the pMal-O-Pip linker and the alcohol signal overlap with the broad water peak (∼3.70–2.70 ppm). Two H-signals of the citrulline amino acid residue overlap with the DMSO signal (∼2.60–2.40 ppm). MS (*m*/*z*) calcd C_39_H_58_N_8_O_9_: (M + H)^+^, 783.44; found, 783.44.

#### 4-((2*S*)-2-((2*S*)-2-(3-(4-(2-(2-(4,7-Dimethyl-1,3-dioxo-1,3,3*a*,4,7,7*a*-hexahydro-2*H*-4,7-epoxyisoindol-2-yl)­ethoxy)­ethyl)­piperazin-1-yl)­propanamido)-3-methylbutanamido)-5-ureidopentanamido)­benzyl (4-nitrophenyl) Carbonate (**pMal-O-Pip-ValCit-PAB-PNP**)

pMal-O-Pip-ValCit-PAB-OH (290 mg, 0.29 mmol, 1.0 equiv) in a dry flask was dissolved in 10 mL dry DMF at room temperature under Ar atmosphere and bis­(4-nitrophenyl) carbonate (267 mg, 0.86 mmol, 3.0 equiv) and DIPEA (0.76 mL, 4.30 mmol, 15.0 equiv) were added. The mixture was stirred for 7 h, then all volatiles were removed in vacuo. The residue was purified by silica column chromatography using a gradient of 10–30% MeOH in DCM as the eluent. The product-containing fractions were combined and 30 mL toluene were added before concentrating them in vacuo. pMal-O-Pip-ValCit-PAB-PNP was obtained as an off-white solid in 88% yield (252 mg containing 5% toluene, 0.25 mmol). ^1^H NMR (500 MHz, DMSO-*d*_6_): δ 10.07 (s, 1H), 8.35–8.29 (m, 2H), 8.22 (bs, 1H), 8.17–8.08 (m, 1H), 7.65 (d, *J* = 8.6 Hz, 2H), 7.59–7.54 (m, 2H), 7.41 (d, *J* = 8.6 Hz, 2H), 6.36 (s, 2H), 5.98 (t, *J* = 5.5 Hz, 1H), 5.42 (s, 2H), 5.24 (s, 2H), 4.39 (dt, *J* = 8.0, 5.2 Hz, 1H), 4.25 (dd, *J* = 8.4, 6.5 Hz, 1H), 3.54–3.38 (m, 6H), 3.07–2.91 (m, 2H), 2.88 (s, 2H), 1.98 (sxt, *J* = 6.8 Hz, 1H), 1.75–1.55 (m, 2H), 1.53 (s, 6H), 1.50–1.32 (m, 2H), 0.87 (d, *J* = 6.8 Hz, 3H), 0.84 (d, *J* = 6.8 Hz, 3H) ppm. The remaining 14 aliphatic H-signals of the pMal-O-Pip linker overlap with the DMSO peak (∼2.70–2.10 ppm). MS (*m*/*z*) calcd C_46_H_61_N_9_O_13_: (M + H)^+^, 948.45; found, 948.42.

#### 4-((2*S*)-2-((2*S*)-2-(3-(4-(2-(2-(4,7-Dimethyl-1,3-dioxo-1,3,3*a*,4,7,7*a*-hexahydro-2*H*-4,7-epoxyisoindol-2-yl)­ethoxy)­ethyl)­piperazin-1-yl)­propanamido)-3-methylbutanamido)-5-ureidopentanamido)­benzyl (2-((2-acrylamido-5-methoxy-4-((4-(1-methyl-1*H*-indol-3-yl)­pyrimidin-2-yl)­amino)­phenyl)­(methyl)­amino)­ethyl)­(methyl)­carbamate (**pMal-O-Pip-ValCit-PAB-OsiNHMe**)

pMal-O-Pip-ValCit-PAB-PNP (250 mg, 0.25 mmol, 1.0 equiv) and OsiNHMe (250 mg, 0.33 mmol, 1.3 equiv) in a dry flask were dissolved in 8.5 mL dry DMF at room temperature under Ar atmosphere and DIPEA (0.22 mL, 1.25 mmol, 5.0 equiv) was added. The mixture was stirred for 4 h, then all volatiles were removed in vacuo. The residue was purified by silica column chromatography using a gradient of 10–30% MeOH in DCM as the eluent. pMal-O-Pip-ValCit-PAB-OsiNHMe was obtained as a pale-yellow solid in 92% yield (327 mg containing 9% MeOH, 0.23 mmol). ^1^H NMR (500 MHz, DMSO-*d*_6_): δ 9.98 (s, 1H), 9.11 (d, *J* = 20.8 Hz, 1H), 8.94 (s, 1H), 8.60 (s, 1H), 8.32 (d, *J* = 5.3 Hz, 1H), 8.26 (d, *J* = 8.0 Hz, 1H), 8.25–8.18 (m, 1H), 8.11 (d, *J* = 7.0 Hz, 1H), 7.87 (s, 1H), 7.62–7.54 (m, 2H), 7.52 (d, *J* = 8.2 Hz, 1H), 7.33–7.26 (m, 2H), 7.26–7.20 (m, 2H), 7.16 (t, *J* = 7.2 Hz, 1H), 6.94 (d, *J* = 22.3 Hz, 1H), 6.64 (dd, *J* = 16.8, 10.2 Hz, 1H), 6.35 (s, 2H), 6.24 (d, *J* = 18.3 Hz, 1H), 5.97 (t, *J* = 5.6 Hz, 1H), 5.75–5.68 (m, 1H), 5.40 (s, 2H), 4.99 (s, 2H), 4.43–4.33 (m, 1H), 4.23 (dd, *J* = 8.4, 6.5 Hz, 1H), 3.90 (s, 3H), 3.85 (d, *J* = 16.3 Hz, 3H), 3.49 (t, *J* = 5.5 Hz, 2H), 3.47–3.37 (m, 6H), 3.08–2.90 (m, 4H), 2.87 (s, 2H), 2.84–2.76 (m, 3H), 2.72–2.61 (m, 3H, overlap with DMSO ^13^C satellite signal), 1.97 (sxt, *J* = 6.7 Hz, 1H), 1.74–1.55 (m, 2H), 1.52 (s, 6H), 1.48–1.31 (m, 2H), 0.86 (d, *J* = 6.8 Hz, 3H), 0.82 (d, *J* = 6.8 Hz, 3H) ppm. The remaining 14 aliphatic H-signals of the pMal-O-Pip linker overlap with the DMSO peak (∼2.60–2.10 ppm). MS (*m*/*z*) calcd C_67_H_87_N_15_O_12_: (M + H)^+^, 1294.67; found, 1294.61.

#### 4-((*S*)-2-((*S*)-2-(3-(4-(2-(2-(2,5-Dioxo-2,5-dihydro-1*H*-pyrrol-1-yl)­ethoxy)­ethyl)­piperazin-1-yl)­propanamido)-3-methylbutanamido)-5-ureidopentanamido)­benzyl (2-((2-acrylamido-5-methoxy-4-((4-(1-methyl-1*H*-indol-3-yl)­pyrimidin-2-yl)­amino)­phenyl)­(methyl)­amino)­ethyl)­(methyl)­carbamate (**Mal-Pip-ValCit**)

pMal-O-Pip-ValCit-PAB-OsiNHMe (311 mg, 0.22 mmol) was dissolved in 5.5 mL DMSO and stirred at 90 °C for 3 h. All volatiles were removed in vacuo and the residue was purified by preparative HPLC on an XBridge BEH C18 OBD Prep Column (19 mm × 250 mm) using a mixture of 32% CH_3_CN and 68% Milli-Q water with 0.1% TFA as the eluent. The product fractions were combined, CH_3_CN removed in vacuo and the aqueous phase lyophilized twice. Mal-Pip-ValCit was obtained as a yellow solid in 65% yield (219 mg, 0.14 mmol). ^1^H NMR (500 MHz, DMSO-*d*_6_): δ 10.06 (s, 1H, H36), 9.91–8.97 (m, 2H, H14 + H22), 8.75 (s, 1H, H1), 8.46 (s, 1H, H20), 8.38–7.95 (m, 4H, H7 + H12 + H39 + H42), 7.66–7.48 (m, 3H, H4 + H34), 7.36 (d, *J* = 6.2 Hz, 1H, H11), 7.28 (t, *J* = 7.2 Hz, 3H, H5 + H33), 7.21–7.11 (m, 1H, H6), 7.04 (s, 2H, H49), 7.03–6.95 (m, 1H, H17), 6.65 (dd, *J* = 16.7, 10.4 Hz, 1H, H24), 6.21 (d, *J* = 17.0 Hz, 1H, H25), 6.08 (s, 1H, H53), 5.93–5.08 (m, 3H, H25 + H55), 4.99 (s, 2H, H31), 4.45–4.34 (m, 1H, H38), 4.26 (t, *J* = 7.5 Hz, 1H, H41), 3.92 (s, 3H, H2), 3.82 (d, *J* = 16.1 Hz, 3H, H21), 3.70–3.62 (m, 2H, H45), 3.59 (t, *J* = 5.1 Hz, 2H, H47), 3.53 (t, *J* = 5.1 Hz, 2H, H46), 3.50–2.84 (m, 18H, H_Pip‑linker_ + H27 + H28 + H52), 2.80 (d, *J* = 14.5 Hz, 3H, H29), 2.74 (d, *J* = 22.2 Hz, 3H, H26), 2.67–2.55 (m, 2H, H44), 1.98 (sxt, *J* = 6.5 Hz, 1H, H56), 1.74–1.52 (m, 2H, H50), 1.50–1.29 (m, 2H, H51), 0.87 (d, *J* = 6.5 Hz, 3H, H57), 0.84 (d, *J* = 6.6 Hz, 3H, H57) ppm. ^13^C NMR (126 MHz, DMSO-*d*_6_): δ 171.05 (C40 + C48), 170.65 (C37), 169.46 (C43), 165.38 (only in 2D, C10), 163.04 (C23), 159.02 (C54), 158.43 (q, *J* = 33.4 Hz, C_q_-TFA), 155.69 + 155.43 (C30 + C30′), 148.80 (only in 2D, C12), 148.96 (only in 2D, C16), 142.10 (only in 2D, C18), 138.66 (C3 or C35), 138.06 (C3 or C35), 137.22 (C1), 134.64 (C49), 132.12 (C24), 131.67 (C32), 128.48 (C33), 126.54 (C25), 125.42 (C8), 125.14 + 125.00 (C19 + C19′), 123.00 (C5), 122.07 (C6 + C7), 119.02 (C20 + C34), 116.58 (q, *J* = 296.3 Hz, CF_3_-TFA), 111.88 (C9), 111.01 (C4), 106.24 (C11), 104.84 + 104.62 (C17 + C17′), 67.32 (C46), 66.09 (C31), 65.12 (C45), 57.54 (C41), 55.94 (C21), 55.14 (C_Pip‑linker_), 53.29 + 52.78 (C27 + C27′), 53.13 (C38), 52.14 (C_Pip‑linker_), 46.55 + 45.91 (C28 + C28′), 41.86 + 41.53 (C26 + C26′), 38.56 (C52), 36.61 (C47), 34.47 + 33.99 (C29 + C29′), 33.48 (C2), 30.72 (C56), 30.36 (C44), 29.35 (C50), 26.91 (C51), 19.24 (C57), 18.19 (C57) ppm. C13, C15 and four C_Pip‑linker_ signals could not be observed. HRMS (*m*/*z*) calcd C_61_H_79_N_15_O_11_: (M + H)^+^, 1198.6156; found, 1198.6159. Elemental analysis (%) Calcd for C_61_H_79_N_15_O_11_*3TFA: C, 52.24; H, 5.37; N, 13.64. Found: C, 51.93; H, 5.33; N, 13.34.

#### *Tert*-Butyl 1-(2,5-Dioxo-2,5-dihydro-1*H*-pyrrol-1-yl)-3,6,9,12,15,18-hexaoxahenicosan-21-oate (**Mal-PEG**_**6**_ **-COO** ^*t*^ **Bu**)

*Tert*-butyl 1-amino-3,6,9,12,15,18-hexaoxahenicosan-21-oate (1.00 g, 2.39 mmol, 1.0 equiv) was dissolved in 15 mL CHCl_3_ and stirred in an ice bath for 10 min. *N*-Methoxycarbonylmaleimide (0.76 g, 4.79 mmol, 2.0 equiv), tetrabutylammonium hydrogen sulfate (0.73 g, 2.15 mmol, 0.9 equiv) and NEt_3_ (0.44 mL. 3.11 mmol, 1.3 equiv) were added in this order and stirred in the cold for 15 min. Thirty mL of saturated NaHCO_3_ solution were added and the mixture was stirred vigorously for 10 min in the cold, before allowing the mixture to warm up to room temperature. Stirring was continued for 20 h, then the phases were separated and the aqueous layer extracted 2× with 20 mL CHCl_3_. The combined organic phases were concentrated in vacuo to give the crude product as a red oil, which was purified by silica column chromatography using 10% MeOH in EtOAc as the eluent. Mal-PEG_6_-COO^*t*^Bu was obtained as a clear oil in 89% yield (1.04 g, 2.13 mmol). ^1^H NMR (500 MHz, DMSO-*d*_6_): δ 7.03 (s, 2H), 3.60–3.54 (m, 4H), 3.53–3.44 (m, 22H), 2.41 (t, *J* = 6.2 Hz, 2H), 1.39 (s, 9H) ppm. MS (*m*/*z*) calcd C_23_H_39_NO_10_: (M + Na)^+^, 512.25; found, 512.28.

#### *Tert*-Butyl 1-(4,7-Dimethyl-1,3-dioxo-1,3,3*a*,4,7,7*a*-hexahydro-2*H*-4,7-epoxyisoindol-2-yl)-3,6,9,12,15,18-hexaoxahenicosan-21-oate (**pMal-PEG**_**6**_ **-COO** ^*t*^ **Bu**)

Mal-PEG_6_-COO^*t*^Bu (1.03 g, 2.10 mmol, 1.0 equiv) was dissolved in 35 mL CH_3_CN and 2,5-dimethylfuran (2.3 mL, 21.04 mmol, 10.0 equiv) was added. The mixture was heated at 60 °C for 24 h, then all volatiles were removed in vacuo. pMal-PEG_6_-COO^*t*^Bu was obtained as a yellow oil in 93% yield (1.19 g, 1.96 mmol). The molar ratio of exo/endo was 1.00:0.28, according to NMR. ^1^H NMR (500 MHz, DMSO-*d*_6_): δ 6.37 (s, 1.47H, C*H*_mal‑exo_), 6.22 (s, 0.40H, C*H*_mal‑endo_), 3.58 (t, *J* = 6.2 Hz, 2H), 3.53–3.42 (m, 24H), 3.27 (s, 0.43H, C*H*_endo_), 2.89 (s, 1.44H, C*H*_exo_), 2.41 (t, *J* = 6.2 Hz, 2H), 1.63 (s, 1.22H, C*H*_3,endo_), 1.53 (s, 4.50H, C*H*_3,exo_), 1.39 (s, 9H) ppm. Ca. 4% of deprotected maleimide species could be observed at 7.03 ppm. MS (*m*/*z*) calcd C_29_H_47_NO_11_: (M + Na)^+^, 608.30; found, 608.32.

#### 1-(4,7-Dimethyl-1,3-dioxo-1,3,3*a*,4,7,7*a*-hexahydro-2*H*-4,7-epoxyisoindol-2-yl)-3,6,9,12,15,18-hexaoxahenicosan-21-oic Acid (**pMal-PEG**_**6**_ **-COOH**)

pMal-PEG_6_-COO^*t*^Bu (1.18 g, 1.94 mmol, 1.0 equiv) was dissolved in 10 mL DCM and TFA (5 mL, 64.25 mmol, 33.2 equiv) was added. The mixture was stirred at room temperature for 1.5 h, then all volatiles were removed and the residue purified by silica column chromatography using a gradient of 2–10% MeOH in DCM as the eluent. The product-containing fractions were combined and concentrated. The resulting brown oil was dissolved in Milli-Q water, causing a phase separation of the product in water and a dark brown liquid. The aqueous phase was separated and lyophilized. pMal-PEG_6_-COOH was obtained as a light-orange oil in 83% yield (0.92 g, 1.61 mmol). The molar ratio of exo/endo was 1.00:0.22, according to NMR. ^1^H NMR (500 MHz, DMSO-*d*_6_): δ 12.14 (bs, 1H), 6.37 (s, 1.52H, C*H*_mal‑exo_), 6.22 (s, 0.34H, C*H*_mal‑endo_), 3.59 (t, *J* = 6.4 Hz, 2H), 3.52–3.43 (m, 24H), 3.27 (s, 0.36H, C*H*_endo_), 2.89 (s, 1.53H, C*H*_exo_), 2.43 (t, *J* = 6.4 Hz, 2H), 1.63 (s, 1.05H, C*H*_3,endo_), 1.53 (s, 4.72H, C*H*_3,exo_) ppm. Ca. 7% of deprotected maleimide species could be observed at 7.03 ppm. MS (*m*/*z*) calcd C_25_H_39_NO_11_: (M + Na)^+^, 552.24; found, 552.27.

#### 1-(4,7-Dimethyl-1,3-dioxo-1,3,3*a*,4,7,7*a*-hexahydro-2*H*-4,7-epoxyisoindol-2-yl)-*N*-(2-((2-((4-(hydroxymethyl)­phenyl)­amino)-2-oxoethyl)­amino)-2-oxoethyl)-3,6,9,12,15,18-hexaoxahenicosan-21-amide (**pMal-PEG**_**6**_ **-GlyGly-PAB-OH**)

pMal-PEG_6_-COOH (100 mg, 0.18 mmol, 1.0 equiv) in a dry flask was dissolved in 3.5 mL dry DMF at room temperature under Ar atmosphere and NEt_3_ (0.25 mL, 1.76 mmol, 10.0 equiv) and EDC·HCl (52 mg, 0.26 mmol, 1.5 equiv) were added. The mixture was stirred for 10 min, then HOBt·H_2_O (41 mg, 0.26 mmol, 1.5 equiv) was added and stirring was continued for another 10 min. Finally, H_2_N-GlyGly-PAB-OH (63 mg, 0.26 mmol, 1.5 equiv, see above) was added and the mixture was stirred for 20 h. All volatiles were removed in vacuo and the residue purified by preparative HPLC on an XBridge BEH C18 OBD Prep Column (19 mm × 250 mm) using a mixture of 22% CH_3_CN and 78% Milli-Q water with 0.1% TFA as the eluent. The product fractions were combined, CH_3_CN removed in vacuo and the aqueous phase lyophilized twice. pMal-PEG_6_-GlyGly-PAB-OH was obtained as a white solid in 81% yield (106 mg, 0.14 mmol). The molar ratio of exo/endo was 1.00:0.16, according to NMR. ^1^H NMR (500 MHz, DMSO-*d*_6_): δ 9.73 (s, 1H), 8.26 (t, *J* = 5.6 Hz, 1H), 8.17 (t, *J* = 5.7 Hz, 1H), 7.56 (d, *J* = 8.5 Hz, 2H), 7.24 (d, *J* = 8.5 Hz, 2H), 6.36 (s, 1.69H, C*H*_mal‑exo_), 6.22 (s, 0.26H, C*H*_mal‑endo_), 5.06 (bs, 1H), 4.43 (s, 2H), 3.88 (d, *J* = 5.9 Hz, 2H), 3.74 (d, *J* = 5.7 Hz, 2H), 3.62 (t, *J* = 6.5 Hz, 2H), 3.53–3.43 (m, 24H), 3.27 (s, 0.32H, C*H*_endo_), 2.89 (s, 1.69H, C*H*_exo_), 2.42 (t, *J* = 6.5 Hz, 2H), 1.63 (s, 0.80H, C*H*_3,endo_), 1.53 (s, 5.19H, C*H*_3,exo_) ppm. Ca. 2% of deprotected maleimide species could be observed at 7.02 ppm. MS (*m*/*z*) calcd C_36_H_52_N_4_O_13_: (M + Na)^+^, 771.34; found, 771.36.

#### 4-(1-(4,7-Dimethyl-1,3-dioxo-1,3,3*a*,4,7,7*a*-hexahydro-2*H*-4,7-epoxyisoindol-2-yl)-21,24-dioxo-3,6,9,12,15,18-hexaoxa-22,25-diazaheptacosan-27-amido)­benzyl (4-nitrophenyl) Carbonate (**pMal-PEG**_**6**_ **-GlyGly-PAB-PNP**)

pMal-PEG_6_-GlyGly-PAB-OH (97 mg, 0.13 mmol, 1.0 equiv) in a dry flask was dissolved in 4.5 mL dry DMF at room temperature under Ar atmosphere and bis­(4-nitrophenyl) carbonate (121 mg, 0.39 mmol, 3.0 equiv) and DIPEA (0.34 mL, 1.94 mmol, 15.0 equiv) were added. The mixture was stirred for 3 h, then all volatiles were removed in vacuo. The residue was purified by silica column chromatography using 8% MeOH in DCM as the eluent. The product-containing fractions were combined and 10 mL toluene were added before concentrating them in vacuo. pMal-PEG_6_-GlyGly-PAB-PNP was obtained as a white waxy solid in 89% yield (105 mg, 0.11 mmol). The molar ratio of exo/endo was 1.00:0.13, according to NMR. ^1^H NMR (500 MHz, DMSO-*d*_6_): δ 9.89 (s, 1H), 8.34–8.29 (m, 2H), 8.27 (t, *J* = 5.6 Hz, 1H), 8.19 (t, *J* = 5.8 Hz, 1H), 7.67 (d, *J* = 8.5 Hz, 2H), 7.59–7.54 (m, 2H), 7.42 (d, *J* = 8.5 Hz, 2H), 6.36 (s, 1.69H, C*H*_mal‑exo_), 6.22 (s, 0.22H, C*H*_mal‑endo_), 5.25 (s, 2H), 3.90 (d, *J* = 5.8 Hz, 2H), 3.75 (d, *J* = 5.7 Hz, 2H), 3.62 (t, *J* = 6.5 Hz, 2H), 3.52–3.43 (m, 24H), 3.27 (s, 0.24H, C*H*_endo_), 2.88 (s, 1.70H, C*H*_exo_), 2.42 (t, *J* = 6.5 Hz, 2H), 1.62 (s, 0.67H, C*H*_3,endo_), 1.53 (s, 5.17H, C*H*_3,exo_). Ca. 4% of deprotected maleimide species could be observed at 7.02 ppm. MS (*m*/*z*) calcd C_43_H_55_N_5_O_17_: (M + Na)^+^, 936.35; found, 936.35.

#### 4-(1-(4,7-Dimethyl-1,3-dioxo-1,3,3*a*,4,7,7*a*-hexahydro-2*H*-4,7-epoxyisoindol-2-yl)-21,24-dioxo-3,6,9,12,15,18-hexaoxa-22,25-diazaheptacosan-27-amido)­benzyl (2-((2-acrylamido-5-methoxy-4-((4-(1-methyl-1*H*-indol-3-yl)­pyrimidin-2-yl)­amino)­phenyl)­(methyl)­amino)­ethyl)­(methyl)­carbamate (**pMal-PEG**_**6**_ **-GlyGly-PAB-OsiNHMe**)

pMal-PEG_6_-GlyGly-PAB-PNP (97 mg, 0.11 mmol, 1.0 equiv) and OsiNHMe (106 mg, 0.14 mmol, 1.3 equiv) in a dry flask were dissolved in 5 mL dry DMF at room temperature under Ar atmosphere and DIPEA (94 μL, 0.53 mmol, 5.0 equiv) was added. The mixture was stirred for 2 h, then all volatiles were removed in vacuo. The residue was purified by silica column chromatography using 7% MeOH in DCM as the eluent. pMal-PEG_6_-GlyGly-PAB-OsiNHMe was obtained as a yellow sticky foam in 95% yield (152 mg containing 16% DIPEA·TFA salt, 0.10 mmol). The molar ratio of exo/endo was 1.00:0.11, according to NMR. ^1^H NMR (500 MHz, DMSO-*d*_6_): δ 9.81 (s, 1H), 9.11 (d, *J* = 23.7 Hz, 1H), 8.94 (s, 1H), 8.61 (s, 1H), 8.32 (d, *J* = 5.3 Hz, 1H), 8.30–8.21 (m, 2H, overlap with DIPEA·TFA signal), 8.17 (t, *J* = 5.7 Hz, 1H), 7.88 (s, 1H), 7.59 (s, 2H), 7.52 (d, *J* = 8.2 Hz, 1H), 7.33–7.26 (m, 2H), 7.26–7.21 (m, 2H), 7.16 (t, *J* = 7.5 Hz, 1H), 6.94 (d, *J* = 22.9 Hz, 1H), 6.64 (dd, *J* = 16.7, 10.3 Hz, 1H), 6.35 (s, 1.73H, C*H*_mal‑exo_), 6.24 (d, *J* = 17.5 Hz, 1H), 6.21 (s, 0.20H, C*H*_mal‑endo_), 5.75–5.66 (m, 1H), 4.99 (s, 2H), 3.90 (s, 3H), 3.88 (d, *J* = 5.9 Hz, 2H), 3.87–3.80 (m, 3H), 3.74 (d, *J* = 5.6 Hz, 2H), 3.61 (t, *J* = 6.3 Hz, 2H, overlap with DIPEA·TFA signal), 3.51–3.43 (m, 24H), 3.40 (t, *J* = 6.5 Hz, 2H), 3.26 (s, 0.29H, C*H*_endo_), 3.09–2.97 (m, 2H), 2.88 (s, 1.77H, C*H*_exo_), 2.85–2.77 (m, 3H), 2.66 (d, *J* = 26.1 Hz, 3H), 2.41 (t, *J* = 6.4 Hz, 2H), 1.62 (s, 0.62H, C*H*_3,endo_), 1.52 (s, 5.32H, C*H*_3,exo_) ppm. Ca. 6% of deprotected maleimide species could be observed at 7.01 ppm. MS (*m*/*z*) calcd C_64_H_81_N_11_O_16_: (M + Na)^+^, 1282.58; found, 1282.58.

#### 4-(1-(2,5-Dioxo-2,5-dihydro-1*H*-pyrrol-1-yl)-21,24-dioxo-3,6,9,12,15,18-hexaoxa-22,25-diazaheptacosan-27-amido)­benzyl (2-((2-acrylamido-5-methoxy-4-((4-(1-methyl-1*H*-indol-3-yl)­pyrimidin-2-yl)­amino)­phenyl)­(methyl)­amino)­ethyl)­(methyl)­carbamate (**Mal-PEG-GlyGly**)

pMal-PEG_6_-GlyGly-PAB-OsiNHMe (135 mg, 0.090 mmol) was dissolved in 2.3 mL DMSO and stirred at 90 °C for 3 h. All volatiles were removed in vacuo and the residue was purified by preparative HPLC on an XBridge BEH C18 OBD Prep Column (19 mm × 250 mm) using a mixture of 35% CH_3_CN and 65% Milli-Q water with 0.1% TFA as the eluent. The product fractions were combined, CH_3_CN removed in vacuo and the aqueous phase lyophilized twice. Mal-PEG-GlyGly was obtained as a yellow solid in 66% yield (77 mg, 0.059 mmol). ^1^H NMR (500 MHz, DMSO-*d*_6_): δ 9.84 (s, 1H, H36), 9.74–8.85 (m, 2H, H14 + H22), 8.75 (s, 1H, H1), 8.48 (bs, 1H, H20), 8.35–8.10 (m, 4H, H7 + H12 + H39 + H42), 7.65–7.50 (m, 3H, H4 + H34), 7.35 (d, *J* = 6.3 Hz, 1H, H11), 7.32–7.23 (m, 3H, H5 + H33), 7.22–7.12 (m, 1H, H6), 7.07–6.90 (m, 3H, H17 + H48), 6.65 (dd, *J* = 16.8, 10.3 Hz, 1H, H24), 6.21 (d, *J* = 17.0 Hz, 1H, H25), 5.78–5.62 (m, 1H, H25), 4.99 (s, 2H, H31), 3.92 (s, 3H, H2), 3.88 (d, *J* = 5.6 Hz, 2H, H38), 3.82 (d, *J* = 18.4 Hz, 3H, H21), 3.74 (d, *J* = 5.6 Hz, 2H, H41), 3.61 (t, *J* = 6.5 Hz, 2H, H45), 3.57–3.53 (m, 2H, H46), 3.52–3.41 (m, 24H, H28 + H_PEG‑linker_), 3.18–3.00 (m, 2H, H27), 2.81 (d, *J* = 15.4 Hz, 3H, H29), 2.73 (d, *J* = 27.4 Hz, 3H, H26), 2.41 (t, *J* = 6.4 Hz, 2H, H44) ppm. ^13^C NMR (126 MHz, DMSO-*d*_6_): δ 171.02 (C43 or C47), 170.96 (C43 or C47), 169.46 (C40), 167.70 (C37), 165.52 (only in 2D, C10), 163.03 (C23), 158.05 (q, *J* = 33.5 Hz, C_q_-TFA), 155.69 + 155.44 (C30 + C30′), 148.95 (only in 2D, C16), 138.49 (C3 or C35), 138.07 (C3 or C35), 137.22 (C1), 134.58 (C48), 132.11 (C24), 131.70 (C32), 128.39 (C33), 126.55 (C25), 125.41 (C8), 125.15 + 125.04 (C19 + C19′), 123.00 (C5), 122.08 (C6 + C7), 119.06 (C20 + C34), 111.87 (C9), 111.02 (C4), 106.24 (C11), 104.85 + 104.65 (C17 + C17′), 69.80 + 69.77 + 69.65 + 69.56 + 69.41 (s) + 66.95 (C_PEG‑linker_), 66.70 (C45), 66.07 (C31), 55.95 (C21), 53.28 + 52.77 (C27 + C27′), 46.57 + 45.89 (C28 + C28′), 42.65 (C38), 42.29 (C41), 41.85 + 41.54 (C26 + C26′), 36.80 (C46), 35.91 (C44), 34.45 + 34.00 (C29 + C29′), 33.48 (C2) ppm. The CF_3_-TFA, C12, C13, C15 and C18 signals could not be observed. HRMS (*m*/*z*) calcd C_58_H_73_N_11_O_15_: (M + H)^+^, 1164.5360; found, 1164.5353. Elemental analysis (%) Calcd for C_58_H_73_N_11_O_15_*TFA*H_2_O: C, 55.59; H, 5.91; N, 11.89. Found: C, 55.59; H, 5.82; N, 11.62.

#### 1-(4,7-Dimethyl-1,3-dioxo-1,3,3*a*,4,7,7*a*-hexahydro-2*H*-4,7-epoxyisoindol-2-yl)-*N*-((*S*)-1-(((*S*)-1-((4-(hydroxymethyl)­phenyl)­amino)-1-oxo-5-ureidopentan-2-yl)­amino)-3-methyl-1-oxobutan-2-yl)-3,6,9,12,15,18-hexaoxahenicosan-21-amide (**pMal-PEG**_**6**_ **-ValCit-PAB-OH**)

pMal-PEG_6_-COOH (100 mg, 0.18 mmol, 1.0 equiv) in a dry flask was dissolved in 3.5 mL dry DMF at room temperature under Ar atmosphere and NEt_3_ (0.25 mL, 1.76 mmol, 10.0 equiv) and EDC·HCl (52 mg, 0.26 mmol, 1.5 equiv) were added. The mixture was stirred for 10 min, then HOBt·H_2_O (41 mg, 0.26 mmol, 1.5 equiv) was added and stirring was continued for another 10 min. Finally, H_2_N-ValCit-PAB-OH (101 mg, 0.26 mmol, 1.5 equiv) was added and the mixture was stirred for 20 h. All volatiles were removed in vacuo and the residue purified by preparative HPLC on an XBridge BEH C18 OBD Prep Column (19 mm × 250 mm) using a mixture of 25% CH_3_CN and 75% Milli-Q water with 0.1% TFA as the eluent. The product fractions were combined, CH_3_CN removed in vacuo and the aqueous phase lyophilized twice. pMal-PEG_6_-ValCit-PAB-OH was obtained as a white solid in 87% yield (136 mg, 0.15 mmol). The molar ratio of exo/endo was 1.00:0.16, according to NMR. ^1^H NMR (500 MHz, DMSO-*d*_6_): δ 9.88 (s, 1H), 8.08 (d, *J* = 7.6 Hz, 1H), 7.87 (d, *J* = 8.6 Hz, 1H), 7.54 (d, *J* = 8.5 Hz, 2H), 7.23 (d, *J* = 8.5 Hz, 2H), 6.36 (s, 1.69H, C*H*_mal‑exo_), 6.22 (s, 0.27H, C*H*_mal‑endo_), 5.98 (s, 1H), 5.40 (bs, 2H), 4.42 (s, 2H), 4.38 (dt, *J* = 7.9, 5.1 Hz, 1H), 4.22 (dd, *J* = 8.4, 6.8 Hz, 1H), 3.64–3.55 (m, 2H, overlap with water signal), 3.53–3.43 (m, 24H, overlap with water signal), 3.27 (s, 0.30H, C*H*_endo_), 3.06–2.90 (m, 2H), 2.89 (s, 1.75H, C*H*_exo_), 2.46 (t, *J* = 7.2 Hz, 1H, overlap with DMSO signal), 2.39 (t, *J* = 6.3 Hz, 1H), 1.96 (sxt, *J* = 6.8 Hz, 1H), 1.74–1.55 (m, 2H), 1.63 (s, 0.91H, C*H*_3,exo_), 1.53 (s, 5.17H, C*H*_3,exo_), 1.49–1.31 (m, 2H), 0.86 (d, *J* = 6.8 Hz, 3H), 0.83 (d, *J* = 6.8 Hz, 3H) ppm. The OH proton could not be observed. Ca. 3% of deprotected maleimide species could be observed at 7.02 ppm. MS (*m*/*z*) calcd C_43_H_66_N_6_O_14_: (M + Na)^+^, 913.45; found, 913.47.

#### 4-((23*S*,26*S*)-1-(4,7-Dimethyl-1,3-dioxo-1,3,3*a*,4,7,7*a*-hexahydro-2*H*-4,7-epoxyisoindol-2-yl)-23-isopropyl-21,24-dioxo-26-(3-ureidopropyl)-3,6,9,12,15,18-hexaoxa-22,25-diazaheptacosan-27-amido)­benzyl (4-Nitrophenyl) carbonate (**pMal-PEG**_**6**_ **-ValCit-PAB-PNP**)

pMal-PEG_6_-ValCit-PAB-OH (125 mg, 0.14 mmol, 1.0 equiv) in a dry flask was dissolved in 4.9 mL dry DMF at room temperature under Ar atmosphere and bis­(4-nitrophenyl) carbonate (131 mg, 0.42 mmol, 3.0 equiv) and DIPEA (0.37 mL, 2.10 mmol, 15.0 equiv) were added. The mixture was stirred for 3 h, then all volatiles were removed in vacuo. The residue was purified by silica column chromatography using 10% MeOH in DCM as the eluent. The product-containing fractions were combined and 15 mL toluene were added before concentrating them in vacuo. pMal-PEG_6_-ValCit-PAB-PNP was obtained as a white waxy solid in 79% yield (123 mg containing 5% toluene residue, 0.11 mmol). The molar ratio of exo/endo was 1.00:0.14, according to NMR. ^1^H NMR (500 MHz, DMSO-*d*_6_): δ 10.05 (s, 1H), 8.34–8.29 (m, 2H), 8.13 (d, *J* = 7.4 Hz, 1H), 7.87 (d, *J* = 8.6 Hz, 1H), 7.65 (d, *J* = 8.5 Hz, 2H), 7.59–7.54 (m, 2H), 7.41 (d, *J* = 8.6 Hz, 2H), 6.36 (s, 1.61H, C*H*_mal‑exo_), 6.22 (s, 0.23H, C*H*_mal‑endo_), 5.98 (t, *J* = 5.7 Hz, 1H), 5.41 (s, 2H), 5.24 (s, 2H), 4.38 (dt, *J* = 7.8, 5.6 Hz, 1H), 4.23 (dd, *J* = 8.4, 6.9 Hz, 1H), 3.64–3.56 (m, 2H), 3.53–3.42 (m, 24H), 3.27 (s, 0.27H, C*H*_endo_), 3.08–2.90 (m, 2H), 2.88 (s, 1.62H, C*H*_exo_), 2.47 (t, *J* = 7.3 Hz, 1H, overlap with DMSO signal), 2.39 (t, *J* = 6.3 Hz, 1H), 1.96 (sxt, *J* = 6.8 Hz, 1H), 1.76–1.55 (m, 2H), 1.63 (s, 0.75H, C*H*_3,endo_), 1.53 (s, 4.98H, C*H*_3,exo_), 1.50–1.32 (m, 2H), 0.86 (d, *J* = 6.8 Hz, 3H), 0.83 (d, *J* = 6.8 Hz, 3H) ppm. Ca. 5% of deprotected maleimide species could be observed at 7.02 ppm. MS (*m*/*z*) calcd C_50_H_69_N_7_O_18_: (M + Na)^+^, 1078.46; found, 1078.45.

#### 4-((23*S*,26*S*)-1-(4,7-Dimethyl-1,3-dioxo-1,3,3*a*,4,7,7*a*-hexahydro-2*H*-4,7-epoxyisoindol-2-yl)-23-isopropyl-21,24-dioxo-26-(3-ureidopropyl)-3,6,9,12,15,18-hexaoxa-22,25-diazaheptacosan-27-amido)­benzyl (2-((2-Acrylamido-5-methoxy-4-((4-(1-methyl-1*H*-indol-3-yl)­pyrimidin-2-yl)­amino)­phenyl)­(methyl)­amino)­ethyl)­(methyl)­carbamate (**pMal-PEG**_**6**_ **-ValCit-PAB-OsiNHMe**)

pMal-PEG_6_-ValCit-PAB-PNP (115 mg, 0.10 mmol, 1.0 equiv) and OsiNHMe (103 mg, 0.13 mmol, 1.3 equiv) in a dry flask were dissolved in 5 mL dry DMF at room temperature under Ar atmosphere and DIPEA (91 μL, 0.52 mmol, 5.0 equiv) was added. The mixture was stirred for 2 h, then all volatiles were removed in vacuo. The residue was purified by silica column chromatography using 10% MeOH in DCM as the eluent. The product-containing fractions were combined, concentrated in vacuo and the residue triturated in a mixture of Et_2_O and EtOAc (1:3). The supernatant was decanted and the residue dried in vacuo. pMal-PEG_6_-ValCit-PAB-OsiNHMe was obtained as a yellow sticky solid in 71% yield (109 mg containing 5% solvent residue from DCM and EtOAc, 0.074 mmol). The molar ratio of exo/endo was 1.00:0.12, according to NMR. ^1^H NMR (500 MHz, DMSO-*d*_6_): δ 9.98 (s, 1H), 9.12 (d, *J* = 19.7 Hz, 1H), 8.93 (s, 1H), 8.61 (s, 1H), 8.31 (d, *J* = 5.3 Hz, 1H), 8.26 (d, *J* = 7.6 Hz, 1H), 8.11 (d, *J* = 7.4 Hz, 1H), 8.00–7.87 (m, 1H), 7.86 (d, *J* = 8.6 Hz, 1H), 7.62–7.54 (m, 2H), 7.52 (d, *J* = 8.2 Hz, 1H), 7.32–7.25 (m, 2H), 7.26–7.21 (m, 2H), 7.16 (t, *J* = 7.5 Hz, 1H), 6.95 (d, *J* = 22.4 Hz, 1H), 6.64 (dd, *J* = 16.8, 10.3 Hz, 1H), 6.35 (s, 1.64H, C*H*_mal‑exo_), 6.24 (d, *J* = 18.0 Hz, 1H), 6.21 (s, 0.19H, C*H*_mal‑endo_), 5.97 (t, *J* = 5.6 Hz, 1H), 5.75–5.68 (m, 1H), 5.40 (s, 2H), 4.99 (s, 2H), 4.41–4.34 (m, 1H), 4.22 (dd, *J* = 8.4, 6.9 Hz, 1H), 3.90 (s, 3H), 3.85 (d, *J* = 16.4 Hz, 3H), 3.62–3.56 (m, 2H), 3.52–3.43 (m, 24H), 3.40 (t, *J* = 6.4 Hz, 2H), 3.27 (s, 0.27H, C*H*_endo_), 3.08–2.90 (m, 4H), 2.88 (s, 1.65H, C*H*_exo_), 2.85–2.76 (m, 3H), 2.67 (d, *J* = 24.8 Hz, 3H), 2.46 (t, *J* = 7.2 Hz, 1H, overlap with DMSO signal), 2.38 (t, *J* = 6.5 Hz, 1H), 1.99–1.92 (m, 1H, overlap with EtOAc signal), 1.74–1.55 (m, 2H), 1.62 (s, 0.65H, C*H*_3,endo_), 1.53 (s, 5.11H, C*H*_3,exo_), 1.48–1.30 (m, 2H), 0.85 (d, *J* = 6.7 Hz, 3H), 0.82 (d, *J* = 6.8 Hz, 3H) ppm. Ca. 7% of deprotected maleimide species could be observed at 7.02 ppm. MS (*m*/*z*) calcd C_71_H_95_N_13_O_17_: (M + Na)^+^, 1424.69; found, 1424.69.

#### 4-((23*S*,26*S*)-1-(2,5-Dioxo-2,5-dihydro-1*H*-pyrrol-1-yl)-23-isopropyl-21,24-dioxo-26-(3-ureidopropyl)-3,6,9,12,15,18-hexaoxa-22,25-diazaheptacosan-27-amido)­benzyl (2-((2-Acrylamido-5-methoxy-4-((4-(1-methyl-1*H*-indol-3-yl)­pyrimidin-2-yl)­amino)­phenyl)­(methyl)­amino)­ethyl)­(methyl)­carbamate (**Mal-PEG-ValCit**)

pMal-PEG_6_-ValCit-PAB-OsiNHMe (97 mg, 0.066 mmol) was dissolved in 1.7 mL DMSO and stirred at 90 °C for 3 h. All volatiles were removed in vacuo and the residue was purified by preparative HPLC on an XBridge BEH C18 OBD Prep Column (19 mm × 250 mm) using a mixture of 37% CH_3_CN and 63% Milli-Q water with 0.1% TFA as the eluent. The product fractions were combined, CH_3_CN removed in vacuo and the aqueous phase lyophilized twice. Mal-PEG-ValCit was obtained as a yellow solid in 64% yield (62 mg, 0.042 mmol). ^1^H NMR (500 MHz, DMSO-*d*_6_): δ 10.00 (s, 1H, H36), 9.91–8.89 (m, 2H, H14 + H22), 8.76 (s, 1H, H1), 8.45 (s, 1H, H20), 8.36–8.16 (m, 2H, H7 + H12), 8.13 (d, *J* = 7.4 Hz, 1H, H39), 7.89 (d, *J* = 8.6 Hz, 1H, H42), 7.65–7.49 (m, 3H, H4 + H34), 7.36 (d, *J* = 6.4 Hz, 1H, H11), 7.32–7.24 (m, 3H, H5 + H33), 7.22–7.14 (m, 1H, H6), 7.02 (s, 2H, H48), 7.07–6.92 (m, 1H, H17), 6.66 (dd, *J* = 16.9, 10.3 Hz, 1H, H24), 6.21 (d, *J* = 17.1 Hz, 1H, H25), 6.02 (s, 1H, H52), 5.71 (d, *J* = 9.7 Hz, 1H, H25), 5.44 (bs, 2H, H54), 4.98 (s, 2H, H31), 4.41–4.34 (m, 1H, H38), 4.25–4.20 (m, 1H, H41), 3.92 (s, 3H, H2), 3.82 (d, *J* = 18.2 Hz, 3H, H21), 3.63–3.53 (m, 4H, H45 + H46), 3.52–3.40 (m, 24H, H28 + H_PEG‑linker_), 3.18–3.05 (m, 2H, H27), 3.05–2.90 (m, 2H, H51), 2.80 (d, *J* = 14.7 Hz, 3H, H29), 2.74 (d, *J* = 22.9 Hz, 3H, H26), 2.48–2.34 (m, 2H, H44), 1.96 (sxt, *J* = 6.7 Hz, 1H, H55), 1.74–1.53 (m, 2H, H49), 1.49–1.30 (m, 2H, H50), 0.85 (d, *J* = 6.7 Hz, 3H, H56), 0.82 (d, *J* = 6.7 Hz, 3H, H56) ppm. ^13^C NMR (126 MHz, DMSO-*d*_6_): δ 171.21 (C40), 170.96 (C47), 170.64 (C37), 170.35 (C43), 165.50 (only in 2D, C10), 163.07 (C23), 158.95 (C53), 158.22 (q, *J* = 33.8 Hz, C_q_-TFA), 155.69 + 155.42 (C30 + C30′), 148.98 (only in 2D, C16), 147.17 (only in 2D, C12), 138.68 (C35), 138.09 (C3), 137.46 (C1), 134.59 (C48), 132.09 (C24), 131.63 (C32), 128.46 (C33), 126.58 (C25), 125.42 (C8), 125.13 + 124.99 (C19 + C19′), 123.07 (C5), 122.16 (C6 + C7), 119.01 (C20 + C34), 116.44 (q, *J* = 295.8 Hz, CF_3_-TFA), 111.84 (C9), 111.05 (C4), 106.20 (C11), 104.87 + 104.65 (C17 + C17′), 69.79 + 69.71 + 69.65 + 69.50 + 69.42 (C_PEG‑linker_), 66.95 (C45 + C_PEG‑linker_), 66,10 (C31), 57.51 (C41), 55.95 (C21), 53.25 + 52.73 (C27 + C27′), 53.15 (C38), 46.55 + 45.90 (C28 + C28′), 41.84 + 41.51 (C26 + C26′), 38.59 (C51), 36.81 (C46), 35.93 (C44), 34.47 + 34.00 (C29 + C29′), 33.52 (C2), 30.63 (C55), 29.27 (C49), 26.88 (C50), 19.21 (C56), 18.15 (C56) ppm. The CF_3_-TFA, C13, C15 and C18 signals could not be observed. HRMS (*m*/*z*) calcd C_65_H_87_N_13_O_16_: (M + H)^+^, 1306.6466; found, 1306.6464. Elemental analysis (%) Calcd for C_65_H_87_N_13_O_16_*1.5TFA: C, 55.28; H, 6.04; N, 12.32. Found: C, 55.15; H, 6.07; N, 12.12.

### HPLC/LC–MS Stability Measurements

200 μM stock solutions of **Mal-Pip-ValCit** and **Mal-Pip-GlyGly** in DMSO were diluted 1:20 (v/v) with 10 mM PB at pH 7.4, yielding a final compound concentration of 10 μM and a total content of 5% (v/v) DMSO. The samples were incubated at 37 °C and measured over 25 h. HPLC runs were performed using a Waters Acquity UPLC BEH C18 column (130Å, 1.7 μm, 3 mm × 50 mm) on a Dionex Thermo Scientific UltiMate 3000 HPLC system, equipped with a SRD-3400 degasser, an HPG-3400RS binary pump, a WPS-3000TBRS autosampler, a TCC-3000SD column oven and a DAD-3000 UV–vis detector. Chromatograms were evaluated at a wavelength of 250 nm. Milli-Q water (mobile phase A) and acetonitrile (mobile phase B), both containing 0.1% TFA, were used as eluents. The flow rate was consistent at 0.6 mL/min for all measurements using the following gradient: 0–0.5 min A 75:25 B, 0.5–5.5 min linear gradient to A 45:55 B, 5.5–6.5 min A 5:95 B, 6.5–9.2 min A 75:25 B. LC–MS analyses of the same samples at the 4 h time points were conducted on a Waters Acquity UPLC BEH C18 column (130 Å, 1.7 μm, 3 mm × 50 mm) on an Agilent 1260 Infinity II system equipped with a Flexible pump, a 1260 VWD UV–vis detector and the LC-MSD system. Chromatograms were evaluated at a wavelength of 250 nm. Milli-Q water (mobile phase A) and acetonitrile (mobile phase B), both containing 0.1% formic acid, were used as eluents. The flow rate was consistent at 0.6 mL/min for all measurements using the following gradient: 0–0.5 min A 75:25 B, 0.5–5.5 min linear gradient to A 45:55 B, 5.5–5.6 min linear gradient to A 5:95 B, 5.6–7.5 min A 5:95 B, 7.5–8.0 min linear gradient to A 75:25 B.

### Cathepsin B Cleavage Assay

Cathepsin B form human liver was purchased from Calbiochem (cat: 219362). The cleavage of **OsiNHMe** was monitored via HPLC on the same instrument and column used for HPLC stability measurements (see above). Chromatograms were evaluated at a wavelength of 250 nm. Milli-Q water (mobile phase A) and acetonitrile (mobile phase B), both containing 0.1% TFA, were used as eluents. The flow rate was consistent at 0.6 mL/min for all measurements using the following gradient: 0–0.5 min A 95:5 B, 0.5–5.5 min linear gradient to A 5:95 B, 5.5–6.5 min A 5:95 B, 6.5–9.2 min A 95:5 B. LC–MS analyses of enzyme-free samples were conducted on the same instrument and column used for HPLC stability measurements (see above). Chromatograms were evaluated at a wavelength of 220 nm. Milli-Q water (mobile phase A) and acetonitrile (mobile phase B), both containing 0.1% formic acid, were used as eluents. The flow rate was consistent at 0.6 mL/min for all measurements using the following gradient: 0–0.5 min A 95:5 B, 0.5–6–0 min linear gradient to A 5:95 B, 6.0–7.0 min A 5:95 B, 7.0–7.1 min linear gradient to A 95:5 B. In the case of preincubation of **Mal-Pip-ValCit** with HSA, the same instrument, column, eluent and gradient as for “HPLC albumin binding studies” was used (see below).

The following solutions were freshly prepared. Activation buffer: 30 mM DTT and 15 mM EDTA-Na_2_ in Milli-Q water. Procedure without albumin preincubation: 5 μL of the cathepsin B solution were mixed with 10 μL of activation buffer and incubated at room temperature for 15 min. 2 min before the first HPLC-injection, the enzyme mixture was diluted with 1.5 mL 25 mM NaOAc buffer containing 1 mM EDTA. Then 7 μL of 10 mM **Mal-Pip-ValCit** or **Mal-Pip-GlyGly** in Milli-Q water were added, yielding a final compound concentration of 46 μM, and the samples incubated in the autosampler at 37 °C. Enzyme-free controls were prepared using 5 μL H_2_O instead of cathepsin B. Procedure with albumin preincubation: 5 μL of 20 mM **Mal-Pip-ValCit** in DMSO was mixed with 15 μL H_2_O, then 980 μL of ∼300 μM HSA (200 g/L Albunorm diluted 1:10 with 10 mM PB, pH 7.4) were added and the sample incubated at 37 °C for 3.5 h. Albumin binding was monitored via HPLC. 5 μL of the cathepsin B solution were mixed with 10 μL of activation buffer and incubated at room temperature for 15 min. 2 min before the first HPLC-injection, the enzyme mixture was diluted with 807 μL 100 mM NaOAc buffer containing 2 mM EDTA. Then 700 μL of the HSA-Mal-Pip-ValCit solution were added, yielding a final compound concentration of 46 μM, and the samples incubated in the autosampler at 37 °C.

### HPLC Albumin-Binding Studies

Incubation with albumin: 2.5 mM stock solutions of **Mal-Pip-ValCit** and **Mal-Pip-GlyGly** in Milli-Q water with 25% DMSO were added (1:50 *v*/*v*) to ∼300 μM HSA (200 g/L albunorm diluted 1:10 with 150 mM PB, pH 7.4) and incubated at 37 °C. Preincubation with NAC: 10 μL of 5 mM *N*-acetylcysteine (NAC) in 150 mM PB (pH 7.4) were combined with 100 μL of 0.5 mM **Mal-Pip-ValCit** in Milli-Q water containing 5% DMSO and incubated at room temperature for ∼10 min. Then, 11 μL of this solution were combined with 89 μL of ∼300 μM HSA (200 g/L Albunorm diluted 1:10 with 150 mM PB, pH 7.4) and incubated at 37 °C. All samples had a final compound concentration of 50 μM with 0.5% v/v DMSO. The samples were analyzed via HPLC. RP-HPLC runs were performed using a Waters Acquity UPLC Peptide BEH C18 column (300 Å, 1.7 μm, 2.1 mm × 100 mm) on a Dionex Thermo Scientific UltiMate 3000 HPLC system, equipped with a SRD-3400 degasser, an HPG-3400RS binary pump, a WPS-3000TRS autosampler, a TCC-3000RS column oven and a DAD-3000RS UV–vis detector. Chromatograms were evaluated at a wavelength of 300 nm. Milli-Q water (mobile phase A) and acetonitrile (mobile phase B), both containing 0.1% TFA, were used as eluents. The flow rate was consistent at 0.4 mL/min for all measurements using the following gradient: 0–0.5 min A 68:32 B, 0.5–6.5 min linear gradient to A 5:95 B, 6.5–7.6 min A 5:95 B, 7.6–7.7 min linear gradient to A 68:32 B, 7.7–9.2 min A 68:32 B.

### UV–Vis Albumin-Binding Studies

To remove *N*-acetyl-dl-tryptophan, caprylic acid and NaCl from Albunorm, 0.5 mL of a 200 g/L albunorm solution were purified via size-exclusion chromatography (Sephadex G25, purchased from Cytiva) using Milli-Q water as the eluent and finally lyophilized. The interaction of the compounds **Mal-Pip-GlyGly**, **Mal-Pip-ValCit** and **OsiNHMe** with the Cys^34^ residue of HSA was investigated via the DTDP method described in our former work.^48^ The available Cys thiol content in HSA was determined to be 33 ± 3%. Compound binding was tested according to the following setup: ca. 6.6 μM Cys^34^ thiol (20 μM HSA) and various equivalents of compound (0, 0.25, 0.5, 0.75, 1.0, 1.5 equiv) were incubated for 20 min at pH 7.40 (100 mM PB) and 37 °C and the UV–vis spectra were recorded (a), then 33 μM DTDP (from 4 mM acidic stock solution) was added and the UV–vis spectra were measured after another 2 min waiting time (b). Spectrum (a) was subtracted from spectrum (b) and the obtained difference spectrum was deconvoluted to the sum of the colored reaction product 2-thiopyridone (2-TP) and the excess DTDP with the solver add-in of MS Excel in the wavelength range 310–400 nm. The calculated concentration of 2-TP (ε_342nm_ = 7870 M^–1^×cm^–1^, own data obtained from reaction with reduced glutathione) corresponds to the concentration of free Cys^34^ thiols in the sample. Time dependence of the maleimide–Cys^34^ interaction was investigated for **Mal-Pip-GlyGly**: HSA and the compound were mixed in a 1:1 ratio (ca. 6 μM) and DTDP was added after 5, 10, 20, 40, 60, and 90 min waiting time. Afterward, the experiment proceeded as described above. UV–Vis spectra were recorded between 200 and 600 nm on an Agilent Cary 3500 spectrophotometer equipped with an eight-position multicell holder unit.

### Osimertinib and OsiNHMe Recovery

2 mM DMSO stock solutions of osimertinib or **OsiNHMe** in were added (1:10 v/v) to either mouse serum, a solution of ∼600 μM HSA (200 g/L albunorm diluted 1:5 with 150 mM PB, pH 7.4) or Milli-Q water and incubated at 37 °C. At 0, 2, 4 and 24h, 20 μL aliquots were sampled and 60 μL acetonitrile were added. The samples were vortexed for 5 s, kept at 4 °C for 10 min and then centrifuged at 4 °C for 10 min at 10,000 rpm. 40 μL of the supernatant were diluted 1:1 *v*/*v* with 0.1% TFA and the samples analyzed by HPLC on the same instrument and column used for HPLC stability measurements (see above). Chromatograms were evaluated at a wavelength of 250 nm. Milli-Q water (mobile phase A) and acetonitrile (mobile phase B), both containing 0.1% TFA, were used as eluents. The flow rate was consistent at 0.6 mL/min for all measurements using the following gradient: 0–0.5 min A 95:5 B, 0.5–5.5 min linear gradient to A 5:95 B, 5.5–6.5 min A 5:95 B, 6.5–8.0 min A 95:5 B.

### Analysis of Covalent HSA–Osimertinib Conjugate

A 0.5 mM DMSO stock solution of osimertinib was added (1:10 *v*/*v*) to a solution of ∼1.5 mM HSA (200 g/L Albunorm diluted 1:1 with 100 mM PB, pH 7.4) and incubated at 37 °C for 24 h. 0.5 mL of the albumin solution were purified using Sephadex G25 Fine, according to the same protocol used for Albunorm purification (see above). A small aliquote of the solid albumin sample was dissolved in 10 μL of 2% acetonitrile in H_2_O and 1 μL injected onto a Waters BioResolve Reversed-Phase Monoclonal Antibody Polyphenyl analytical column (2.1 × 150 mm, 2.7 μm, 450 Å) using a Vanquish Horizon UHPLC system coupled to an Orbitrap QExactive mass spectrometer (Thermo Fisher Scientific) at the Mass Spectrometry Centre of the University of Vienna. The column oven temperature was set to 40 °C. H_2_O (mobile phase A) and acetonitrile/H_2_O 9:1 (mobile phase B), both containing 0.1% FA, were used as eluents. The flow rate was consistent at 0.4 mL/min using the following gradient: 0–20.0 min linear gradient from A 85:15 B to A 40:60 B, 20.0–20.3 min linear gradient to A 10:90 B, 20.3–21.3 min A 10:90 B, 21.3–21.6 min linear gradient to A 85:15 B, 21.6–26.0 min A 85:15 B. The high-resolution MS spectra were recorded in positive ion mode in the range *m*/*z* 700–3000 at a resolution of 17,500 (fwhm at 200*m*/*z*). The following HESI ion source settings were applied: electrospray voltage: 3.5 kV, ion transfer capillary temperature: 275 °C. In-source collision induced dissociation (isCID 40 eV) was used to remove noncovalently bound solvents and salt adducts. Charge state determination and deconvolution of ESI mass-to-charge ratio spectra and determination of the molecular mass of the sample was performed using the MagTran software program.^55^

### Cell-Free Kinase Screening

The EGFR kinase-inhibitory potential of **OsiNHMe**, **OsiNH**_**2**_, **OsiPropNHMe** and osimertinib was evaluated against the double mutant EGFR T790M/L858R kinase using the SelectScreen Biochemical Kinase Profiling Service at Life Technologies (ThermoFisher Scientific, Madison, WI). The compounds were screened in duplicate using the Z′-LYTE Assay with a final DMSO concentration of 1%, in the presence of ATP levels at the apparent ATP-K_m_ of the respective kinase. The EGFR kinase-inhibitory potential of **Mal-Pip-ValCit** and **Mal-Pip-GlyGly** was evaluated against the double-mutant EGFR T790M/L858R kinase using the Kinase Screening Assay Services at Reaction Biology (Freiburg, Germany). The compounds were screened in singlicate using the ^33^PanQinase Activity Assay with a final DMSO concentration of 1%, in the presence of ATP levels at the apparent ATP-K_m_ of the respective kinase.

### Molecular Docking

Molecular docking studies were performed using AutoDock 4.2.^56^ The crystal structure of the EGFR kinase domain mutant “TMLR” (PDB ID: 5CAS) was retrieved from the Protein Data Bank and used as the receptor model. Preparation of both the receptor and ligands was carried out using Molecular Graphics Laboratory (MGL) of The Scripps Research Institute (version 1.5.7). Ligand structures were energy-minimized and optimized using LigPrep (Schrödinger Release 2025–2: LigPrep, Schrödinger, LLC, New York, NY, 2025). A grid box was defined to fully encompass the binding pocket of the protein and to allow for exploration of potential ligand-receptor interactions. The grid dimensions were set to 100 × 100 × 100 points with a spacing of 0.375 Å. Grid centers were based on the center of mass of the cocrystallized ligand, with coordinates: *x* = −51.907, *y* = −0.508, and *z* = −23.173 (PDB ID: 5CAS). Docking calculations were carried out using the Lamarckian Genetic Algorithm. The resulting binding affinities were expressed as estimated free energies of binding in kcal/mol. To validate the docking protocol, the original cocrystallized ligand in 5CAS was removed and redocked into the binding site. The resulting pose showed excellent overlap with the experimental structure, confirming the reliability of the docking setup. Molecular graphics were performed with UCSF ChimeraX, developed by the Resource for Biocomputing, Visualization, and Informatics at the University of California, San Francisco, with support from National Institutes of Health R01-GM129325 and the Office of Cyber Infrastructure and Computational Biology, National Institute of Allergy and Infectious Diseases.^57^

Molecular dynamics (MD) simulations were performed using Desmond,^58^ as implemented in the Schrödinger suite.^59^ All the investigated systems were solvated in a cubic water box with dimensions of 15 Å × 10 Å × 10 Å using the TIP3P water model. First, the total charge of the system was neutralized, and then additional Na^+^ Cl^–^ ions were added to achieve a physiological salt concentration of 0.150 M. Simulations were carried out under *NPT* ensemble conditions at a temperature of 300 K and a pressure of 1 atm. Prior to the production run, the system was relaxed using Desmond’s default relaxation protocol to remove unfavorable contacts and equilibrate the system. The production MD simulation was run for a total of 100 ns, with trajectory frames recorded every 100 ps, resulting in approximately 1000 frames. The simulations were performed using the OPLS4 force field,^60^ as implemented in Desmond. Structural stability and conformational changes of the protein-ligand complexes were evaluated through trajectory analysis, including root-mean-square deviation (RMSD) calculations.

### Cell Culture

Cell-based experiments were conducted using the human NSCLC cell lines H1975 (CRL-5908), H1650 (CRL-5883), HCC827 (CRL-2868), human epidermoid carcinoma cell line A431 (CRL-1555), human colorectal carcinoma cell line HCT116 (CCL-247), human clear cell renal carcinoma cell line Caki-1 (HTB-46), and healthy murine fibroblasts (derived from C57BL/6 mouse in-house). H1975, H1650 and HCC827 cells were cultured in RPMI-1640 media, A431 cells were cultured in Dulbecco’s modified eagle’s medium (DMEM), all supplemented with 10% fetal bovine serum (FBS). HCT116 and Caki-1 cells were cultured in McCoy’s 5A media supplemented with 10% FBS and 1% l-glutamine. Healthy murine fibroblasts were cultured in DMEM supplemented with 10% FBS and 1% l-glutamine. All cells apart from murine fibroblasts were obtained from the American Type Culture Collection (ATCC, Manassas, VA, USA) and maintained at 37 °C in a humidified incubator with 5% CO_2_. *Mycoplasma* contamination was routinely tested for, and all cell lines were used within ten passages from thawing to ensure consistency and reliability of experimental outcomes.

### MTT Viability Assay

Cells were seeded in 96-well plates at a density of 3–4 × 10^4^ cells per well in 100 μL of medium and allowed to adhere overnight at 37 °C in a humidified atmosphere containing 5% CO_2_. Test compounds were initially dissolved in DMSO to prepare 10 mM stock solutions and subsequently diluted in growth medium, ensuring a final DMSO concentration below 1%. After 24 h, cells were exposed to 100 μL of compound dilutions in triplicate, at final concentrations ranging from 0 to 10 μM. Following a 72 h incubation at 37 °C and 5% CO_2_, cell viability was assessed using the MTT assay (EZ4U, Biomedica, Vienna, Austria), according to the manufacturer’s instructions.

### Western Blotting

Western blot analysis was used to evaluate the expression levels of Cathepsin B in H1975, H1650 and Caki-1 cell lines as described previously.^46^

### Immunohistochemistry

Formalin-fixed paraffin-embedded tissue sections were incubated at 65 °C for 10 min, deparaffinized, and rehydrated. For cathepsin B immunohistochemistry, antigen retrieval was performed by boiling the sections in 10 mM citrate buffer (pH 6.0) for 30 min. Sections were then incubated overnight at 4 °C in a humidified chamber with the primary antibody (Cathepsin B (D1C7Y) XP Rabbit mAb or Albumin antibody PA5–85166, Thermo Fisher). Detection of antibody binding was carried out using the UltraVision LP Large Volume Detection System HRP Polymer (Lab Vision), following the manufacturer’s protocol (Thermo Fisher Scientific Inc.). Signal development was performed using liquid DAB and substrate chromogen system (DAB; Dako). For hematoxylin and eosin (H/E) staining, rehydrated sections were subjected to nuclear staining with Harris’ hematoxylin (Merck) for 2 min. Slides were then blued in Scott’s solution (Morphisto) for 45 s. Cytoplasmic counterstaining was performed with Eosin Y (Sigma-Aldrich) for 1 min. Slides were subsequently dehydrated through graded ethanol, cleared in *n*-butyl acetate, and mounted with Entellan mounting medium (Merck). All stained slides were imaged using standard laboratory imaging systems. Images were prepared for presentation using SlideViewer (version 2.9.0.229983), and analyses were performed in QuPath (version 0.6.0).

### Animals

A total of 57 C.B.17^SCID/SCID^ mice (8–16 weeks old purchased from Javier) were utilized for the *in vivo* experiments. Animals were housed under specific pathogen-free (SPF) conditions, and all procedures were conducted within a laminar airflow cabinet to maintain sterility. All animal experiments were carried out in accordance with the guidelines and approvals of the Ethics Committee for the Care and Use of Laboratory Animals at the Medical University of Vienna (Ethic number: 2022–0.770.291). Animal well-being was carefully monitored throughout the study, with daily assessments of body weight and clinical signs of distress.

### Therapy Experiments

For the xenograft experiments, the cancer cells were injected subcutaneously into the right flank of mice at the concentration of 1 × 10^6^ per mouse. Therapy started at a mean tumor volume of 100–150 mm^3^. For the H1975 experiment, osimertinib mesylate (29.8 mg/kg), **OsiNHMe** (37.0 mg/kg), **OsiNH**_**2**_ (36.4 mg/kg) and **OsiPropNHMe** (43.0 mg/kg) were administered per os, all formulated in 0.5% hydroxypropyl methyl cellulose. Control group received 0.5% hydroxypropyl methyl cellulose alone. All groups received drugs five times a week for 2 weeks.

In the H1650 experiment, **Mal-Pip-ValCit** and **Mal-Pip-GlyGly** were administered intravenously at a dose of 83.7 mg/kg and 75.7 mg/kg respectively, while osimertinib mesylate was administered per os at equimolar doses of 29.8 mg/kg. Both **Mal-Pip-ValCit** and **Mal-Pip-GlyGly** were formulated in a vehicle consisting of 20% propylene glycol (PG), 45% of a 5% glucose and 35% murine blood serum, while osimertinib was formulated in 0.5% hydroxypropyl methyl cellulose. Control animals received the 20% PG dissolved in 5% glucose vehicle solution (i.v.). Drugs and control solution were administered two times per week for 2 weeks. Tumor dimensions were measured daily using calipers. Subsequently, the tumor volume was estimated using the established modified ellipsoidal formula: *V* = (1/2) × *L* × *W* ^2^, where *L* is the tumor length and *W* the width. At the conclusion of the experiment, animals were sacrificed by cervical dislocation, and tumors and organs were collected, fixed in 4% formaldehyde (Carl Roth) for 24 h, and embedded in paraffin using a KOS tissue processor (Milestone).

## Supplementary Material









## Notes

Synthesis schemes, NMR spectra, HPLC runs and mass spectra, cell-free and cell-based IC_50_ data, in silico docking data, HPLC and LC–MS runs of stability and cathepsin B cleavage assays, additional albumin-binding data, body weights of *in vivo* therapy experiment (PDF)
Molecular formular strings (smiles) as (PDB)
Molecular formular strings (PDB)
Molecular formular strings (PDB)

## List of Abbreviations

ADC: antibody-drug conjugate
APS: ammonium persulfate
BSA: bovine serum albumin
DAB: 3,3′-diaminobenzidine
DIPEA: *N*,*N*′-diisopropylethylamine
DMEM: Dulbecco’s modified Eagle’s medium
DTDP: 2,2′-dithiodipyridine
EDC·HCl: 1-ethyl-3-(3-(dimethylamino)­propyl)­carbodiimide hydrochloride
EPR effect: enhanced permeability and retention effect
EtOH: ethanol
FA: formic acid
FBS: fetal bovine serum
Fc receptor: neonatal fragment crystallizable receptor
fwhm: full width at half-maximum
H&E: hematoxylin and eosin (stain)
HOBt: *N*-hydroxybenzotriazole
isCID: in-source collision-induced dissociation
MeCN: acetonitrile
MeOH: methanol
MTT: 3-(4,5-dimethylthiazol-2-yl)-2,5-diphenyltetrazolium bromide
NAC: *N*-acetylcysteine
PABC: *para*-aminobenzyl carbonyl
PB: phosphate buffer
PNP: *para*-nitrophenol
PVDF: polyvinylidene difluoride
RMSD: root-mean-square deviation
RPMI: Roswell Park Memorial Institute (medium)
RTK: receptor tyrosine kinase
SCID: severe combined immunodeficiencies
SDS–PAGE: sodium dodecyl sulfate–polyacrylamide gel electrophoresis
SEM: standard error of mean
SPF: specific pathogen-free
TEMED: *N*,*N*,*N*′,*N*′-tetramethylethylenediamine
TBST: tris-buffered saline with 0.1% Tween-20
TKI: tyrosine kinase inhibitor
2-TP: 2-thiopyridone

## References

[1] Zafar A., Khatoon S., Khan M. J., Abu J., Naeem A. (2025). Advancements
and limitations in traditional anti-cancer therapies: a comprehensive
review of surgery, chemotherapy, radiation therapy, and hormonal therapy. Discovery Oncol..

[2] Larsen M. T., Kuhlmann M., Hvam M. L., Howard K. A. (2016). Albumin-based drug
delivery: harnessing nature to cure disease. Mol. Cell. Ther..

[3] Kratz F. (2008). Albumin as
a drug carrier: design of prodrugs, drug conjugates and nanoparticles. J. Controlled Release.

[4] Quinlan G. J., Martin G. S., Evans T. W. (2005). Albumin: biochemical
properties and
therapeutic potential. Hepatology.

[5] Chaudhury C., Mehnaz S., Robinson J. M., Hayton W. L., Pearl D. K., Roopenian D. C., Anderson C. L. (2003). The major histocompatibility complex–related
Fc receptor for IgG (FcRn) binds albumin and prolongs its lifespan. J. Exp. Med..

[6] Finicle B. T., Jayashankar V., Edinger A. L. (2018). Nutrient scavenging in cancer. Nat. Rev. Cancer.

[7] Spada A., Emami J., Tuszynski J. A., Lavasanifar A. (2021). The uniqueness
of albumin as a carrier in nanodrug delivery. Mol. Pharmaceutics.

[8] Hoogenboezem E. N., Duvall C. L. (2018). Harnessing albumin
as a carrier for cancer therapies. Adv. Drug
Delivery Rev..

[9] Elsadek B., Kratz F. (2012). Impact of albumin on
drug delivery—New applications on the
horizon. J. Controlled Release.

[10] Kronberger J., Balber T., Schueffl H., Wahrmann R., Federa A., Gradl M., Brandt M. R., Wanek T., Mitterhauser M., Kowol C. R. (2025). Site-Selectively
Functionalized Albumin with
DFO* Maleimide for 89Zr-Radiolabeling Yields a Metabolically Stable
PET Probe that Enables Late Time-Point Tumor Imaging in Mice. J. Med. Chem..

[11] Green M., Manikhas G., Orlov S., Afanasyev B., Makhson A., Bhar P., Hawkins M. (2006). Abraxane®, a novel
Cremophor®-free, albumin-bound particle form of paclitaxel for
the treatment of advanced non-small-cell lung cancer. Ann. Oncol..

[12] Kratz F., Müller-Driver R., Hofmann I., Drevs J., Unger C. (2000). A novel macromolecular
prodrug concept exploiting endogenous serum albumin as a drug carrier
for cancer chemotherapy. J. Med. Chem..

[13] Di
Stefano G., Lanza M., Kratz F., Merina L., Fiume L. (2004). A novel method for coupling doxorubicin to lactosaminated human albumin
by an acid sensitive hydrazone bond: synthesis, characterization and
preliminary biological properties of the conjugate. Eur. J. Pharm. Sci..

[14] Lemmon M. A., Schlessinger J. (2010). Cell signaling by receptor tyrosine
kinases. Cell.

[15] Thomson, R. J. ; Moshirfar, M. ; Ronquillo, Y. Tyrosine Kinase Inhibitors. In StatPearls [Internet]; StatPearls Publishing, 2023. 33090752

[16] Roskoski R. (2025). Properties of
FDA-approved small molecule protein kinase
inhibitors: A 2025 update. Pharmacol. Res..

[17] Padma V. V. (2015). An overview
of targeted cancer therapy. Biomedicine.

[18] Hussain S., Mursal M., Verma G., Hasan S. M., Khan M. F. (2024). Targeting
oncogenic kinases: Insights on FDA approved tyrosine kinase inhibitors. Eur. J. Pharmacol..

[19] Zhang N., Li Y. (2023). Receptor tyrosine kinases:
Biological functions and anticancer targeted
therapy. MedComm.

[20] Shyam
Sunder S., Sharma U. C., Pokharel S. (2023). Adverse effects of
tyrosine kinase inhibitors in cancer therapy: pathophysiology, mechanisms
and clinical management. Signal Transduct. Target.
Ther..

[21] Hartmann J. T., Haap M., Kopp H.-G., Lipp H.-P. (2009). Tyrosine kinase
inhibitors-a review on pharmacology, metabolism and side effects. Curr. Drug Metab..

[22] Di
Gion P., Kanefendt F., Lindauer A., Scheffler M., Doroshyenko O., Fuhr U., Wolf J., Jaehde U. (2011). Clinical pharmacokinetics
of tyrosine kinase inhibitors: focus on pyrimidines, pyridines and
pyrroles. Clin. Pharmacokinet..

[23] Dömötör O., Pelivan K., Borics A., Keppler B. K., Kowol C. R., Enyedy E. ´. A. (2018). Comparative studies on the human serum albumin binding
of the clinically approved EGFR inhibitors gefitinib, erlotinib, afatinib,
osimertinib and the investigational inhibitor KP2187. J. Pharm. Biomed. Anal..

[24] van
Erp N. P., Gelderblom H., Guchelaar H.-J. (2009). Clinical
pharmacokinetics of tyrosine kinase inhibitors. Cancer Treat. Rev..

[25] Ghinea N. (2021). Anti-angiogenic
therapy: albumin-binding proteins could mediate mechanisms underlying
the accumulation of small molecule receptor tyrosine kinase inhibitors
in normal tissues with potential harmful effects on health. Diseases.

[26] Villarroel M. C., Pratz K. W., Xu L., Wright J. J., Smith B. D., Rudek M. A. (2012). Plasma protein binding
of sorafenib, a multi kinase
inhibitor: in vitro and in cancer patients. Invest. New Drugs.

[27] Li J., Brahmer J., Messersmith W., Hidalgo M., Baker S. D. (2006). Binding
of gefitinib, an inhibitor of epidermal growth factor receptor-tyrosine
kinase, to plasma proteins and blood cells: in vitro and in cancer
patients. Invest. New Drugs.

[28] Qi T., Cao Y. (2023). Dissecting sources
of variability in patient response to targeted
therapy: anti-HER2 therapies as a case study. Eur. J. Pharm. Sci..

[29] Summerfield S. G., Yates J. W., Fairman D. A. (2022). Free drug
theory–no longer
just a hypothesis?. Pharm. Res..

[30] Webborn P. J., Beaumont K., Martin I. J., Smith D. A. (2025). Free drug concepts:
a lingering problem in drug discovery. J. Med.
Chem..

[31] Idasiak-Piechocka I., Lewandowski D., Świgut W., Kalinowski J., Mikosza K., Suchowiejski P., Szałek E., Karbownik A., Miedziaszczyk M. (2025). Effect of hypoalbuminemia on drug
pharmacokinetics. Front. Pharmacol..

[32] Dubowchik G. M., Firestone R. A. (1998). Cathepsin B-sensitive dipeptide prodrugs.
1. A model
study of structural requirements for efficient release of doxorubicin. Bioorg. Med. Chem. Lett..

[33] Dubowchik G. M., Mosure K., Knipe J. O., Firestone R. A. (1998). Cathepsin
B-sensitive dipeptide prodrugs. 2. Models of anticancer drugs paclitaxel
(Taxol®), mitomycin C and doxorubicin. Bioorg. Med. Chem. Lett..

[34] Shen L., Sun X., Chen Z., Guo Y., Shen Z., Song Y., Xin W., Ding H., Ma X., Xu W. (2024). ADCdb:
the database of antibody–drug conjugates. Nucleic Acids Res..

[35] Gondi C. S., Rao J. S. (2013). Cathepsin B as a cancer target. Expert Opin. Ther. Targets.

[36] Vasiljeva O., Papazoglou A., Krüger A., Brodoefel H., Korovin M., Deussing J., Augustin N., Nielsen B. S., Almholt K., Bogyo M. (2006). Tumor
cell–derived
and macrophage-derived cathepsin B promotes progression and lung metastasis
of mammary cancer. Cancer Res..

[37] Aggarwal N., Sloane B. F. (2014). Cathepsin B: multiple
roles in cancer. Proteomics Clin. Appl..

[38] 2018. https://www.fda.gov/drugs/resources-information-approved-drugs/fda-approves-osimertinib-first-line-treatment-metastatic-nsclc-most-common-egfr-mutations (accessed July 16, 2025).

[39] Finlay M.
R. V., Anderton M., Ashton S., Ballard P., Bethel P. A., Box M. R., Bradbury R. H., Brown S. J., Butterworth S., Campbell A. (2014). Discovery of a potent and selective EGFR inhibitor
(AZD9291) of both sensitizing and T790M resistance mutations that
spares the wild type form of the receptor. J.
Med. Chem..

[40] Tang Z.-H., Jiang X.-M., Guo X., Fong C. M. V., Chen X., Lu J.-J. (2016). Characterization
of osimertinib (AZD9291)-resistant non-small cell
lung cancer NCI-H1975/OSIR cell line. Oncotarget.

[41] Cross D. A. E., Ashton S. E., Ghiorghiu S., Eberlein C., Nebhan C. A., Spitzler P. J., Orme J. P., Finlay M. R. V., Ward R. A., Mellor M. J. (2014). AZD9291,
an irreversible EGFR TKI, overcomes
T790M-mediated resistance to EGFR inhibitors in lung cancer. Cancer Discov..

[42] Caban M., Fronik P., Terenzi A., Federa A., Bormio Nunes J. H., Pitek R., Kirchhofer D., Schueffl H. H., Berger W., Keppler B. K. (2025). A new
fluorescent oxaliplatin (iv) complex
with EGFR-inhibiting properties for the treatment of drug-resistant
cancer cells. Inorg. Chem. Front..

[43] Heald R., Bowman K. K., Bryan M. C., Burdick D., Chan B., Chan E., Chen Y., Clausen S., Dominguez-Fernandez B., Eigenbrot C. (2015). Noncovalent mutant selective epidermal growth
factor receptor inhibitors: a lead optimization case study. J. Med. Chem..

[44] Elduque X., Sanchez A., Sharma K., Pedroso E., Grandas A. (2013). Protected
maleimide building blocks for the decoration of peptides, peptoids,
and peptide nucleic acids. Bioconjugate Chem..

[45] Dijkstra M., Schueffl H., Federa A., Kast C., Unterlercher A., Keppler B. K., Heffeter P., Kowol C. R. (2025). Novel Maleimide
Linkers Based on a Piperazine Motif for Strongly Increased Aqueous
Solubility. ACS Omega.

[46] Karnthaler-Benbakka C., Koblmüller B., Mathuber M., Holste K., Berger W., Heffeter P., Kowol C. R., Keppler B. K. (2019). Synthesis,
characterization
and in vitro studies of a cathepsin B-cleavable prodrug of the VEGFR
inhibitor sunitinib. Chem. Biodivers..

[47] Gulikers J. L., Veerman G. D. M., Jebbink M., Kruithof P. D., Steendam C. M., Boosman R. J., Mathijssen R. H., Tjan-Heijnen V. C., Driessen J. H., Dursun S. (2024). Osimertinib
plasma trough
concentration in relation to brain metastases development in patients
with advanced EGFR-mutated NSCLC. JTO Clin.
Res. Rep..

[48] Pichler V., Mayr J., Heffeter P., Dömötör O., Enyedy É. A., Hermann G., Groza D., Köllensperger G., Galanksi M., Berger W. (2013). Maleimide-functionalised
platinum (IV) complexes as a synthetic platform for targeted drug
delivery. Chem. Commun..

[49] Ashraf S., Qaiser H., Tariq S., Khalid A., Makeen H. A., Alhazmi H. A., Ul-Haq Z. (2023). Unraveling the versatility of human
serum albumin – A comprehensive review of its biological significance
and therapeutic potential. Curr. Res. Struct.
Biol..

[50] van
Veelen A., van Geel R., de Beer Y., Dingemans A. M., Stolk L., Ter Heine R., de Vries F., Croes S. (2020). Validation
of an analytical method using HPLC–MS/MS to quantify osimertinib
in human plasma and supplementary stability results. Biomed. Chromatogr..

[51] Wu Y., Chen L., Chen J., Xue H., He Q., Zhong D., Diao X. (2023). Covalent binding mechanism of furmonertinib
and osimertinib with human serum albumin. Drug
Metab. Dispos..

[52] Liu X., Feng D., Zheng M., Cui Y., Zhong D. (2020). Characterization
of covalent binding of tyrosine kinase inhibitors to plasma proteins. Drug Metab. Pharmacokinet..

[53] Levitt D. G., Levitt M. D. (2016). Human serum albumin homeostasis:
a new look at the
roles of synthesis, catabolism, renal and gastrointestinal excretion,
and the clinical value of serum albumin measurements. Int. J. Gen. Med..

[54] Wiessler, M. ; Kliem, H.-C. ; Sauerbrei, B. ; Schmauser, B. Method for producing water-soluble saccharide conjugates and saccharide mimetics by diels-alder reaction. WO 2002016378 A1, 2002.

[55] Zhang Z., Marshall A. G. (1998). A universal algorithm for fast and automated charge
state deconvolution of electrospray mass-to-charge ratio spectra. J. Am. Soc. Mass Spectrom..

[56] Morris G. M., Huey R., Lindstrom W., Sanner M. F., Belew R. K., Goodsell D. S., Olson A. J. (2009). AutoDock4
and AutoDockTools4: Automated
docking with selective receptor flexibility. J. Comput. Chem..

[57] Goddard T. D., Huang C. C., Meng E. C., Pettersen E. F., Couch G. S., Morris J. H., Ferrin T. E. (2018). UCSF ChimeraX: Meeting
modern challenges in visualization and analysis. Protein Sci..

[58] Bowers, K. J. ; Chow, E. ; Xu, H. ; Dror, R. O. ; Eastwood, M. P. ; Gregersen, B. A. ; Klepeis, J. L. ; Kolossvary, I. ; Moraes, M. A. ; Sacerdoti, F. D. Scalable algorithms for molecular dynamics simulations on commodity clusters. Proceedings of the 2006 ACM/IEEE Conference on Supercomputing; ACM, 2006; p 84.

[59] Schrödinger Release 2025–4: Desmond Molecular Dynamics System, D. E. Shaw Research, New York, NY, 2024; Maestro-Desmond Interoperability Tools, Schrödinger: New York, NY, 2025.

[60] Lu C., Wu C., Ghoreishi D., Chen W., Wang L., Damm W., Ross G. A., Dahlgren M. K., Russell E., Von Bargen C. D. (2021). OPLS4: Improving Force Field Accuracy on Challenging
Regimes of Chemical
Space. J. Chem. Theory Comput..

