# Supplementary material for: Tumor-Targeted Delivery of an EGFR Inhibitor Prodrug via Site-Specific Albumin Conjugation
Source: J Med Chem. 2026 Jan 23;69(3):2554–74. doi: 10.1021/acs.jmedchem.5c02536 (PMC12910647; doi:10.1021/acs.jmedchem.5c02536)
Supplement: Supplementary file 1 [file jm5c02536_si_001.pdf]

Supporting Information

Tumor-Targeted Delivery of an EGFR Inhibitor Prodrug via Site-Specific Albumin Conjugation

Anja Federa[+,a,b], Rastislav Pitek[+,c], Orsolya Dömötör[d], Éva A. Enyedy[d], Alessio Terenzi[e], Monika Caban[c] , Alessia Stefanelli,[c] Luisa D'Anna[e], Faye White[c], Petra Heffeter*[,c,f] and Christian R. Kowol*[,a,f]

[a] Institute of Inorganic Chemistry, Faculty of Chemistry, University of Vienna, Waehringer Str. 42, A-1090 Vienna, Austria.

[b] Vienna Doctoral School in Chemistry, University of Vienna, Waehringer Str. 42, 1090 Vienna, Austria.

[c] Center for Cancer Research and Comprehensive Cancer Center, Medical University of Vienna, Borschkegasse 8a, A-1090 Vienna, Austria.

[d] Department of Molecular and Analytical Chemistry, University of Szeged, Dóm tér 7-8. H-6720, Szeged, Hungary.

[e] University of Palermo, Department of Biological, Chemical and Pharmaceutical Sciences and Technologies; Viale delle Scienze, Edificio 17, 90128 Palermo, Italy.

[f] Research Cluster "Translational Cancer Therapy Research", 1090 Vienna, Austria.

+ Contributed equally to the main findings of the manuscript.

* Corresponding authors: christian.kowol@univie.ac.at, petra.heffeter@meduniwien.ac.at

Contents

Scheme S1: Synthesis of the osimertinib derivatives S3

Figure S1: Cell-free IC_{50} data of EGFR inhibitors S4

Figure S2: Cell viability data assessed by MTT assay. S4

Figure S3: Cell viability data assessed by short-term MTT assay. S5

Figure S4: *In silico* molecular docking and dynamics data.. S5

Figure S5: Mass spectra of pMal-O-Pip-GlyGly-PAB-PNP and pMal-O-Pip-ValCit-PAB-PNP .. S6

Figure S6: Mass spectra of pMal-O-Pip-GlyGly-PAB-PNP and pMal-O-Pip-ValCit-PAB-PNP .. S7

Scheme S2: Synthesis of the target compounds **Mal-PEG-GlyGly** and **Mal-PEG-ValCit**......... S8

Figure S7: LC-MS spectra of **Mal-Pip-ValCit** and **Mal-Pip-GlyGly** stability kinetics. S9

Figure S8: HPLC stability measurements of **Mal-Pip-ValCit** and **Mal-Pip-GlyGly** S10

Figure S9: Cell-free IC_{50} measurements of **Mal-Pip-GlyGly** and **Mal-Pip-ValCit**................ S111

Figure S10: Cathepsin B cleavage assay of **Mal-Pip-ValCit**... S12

Figure S11: LC-MS chromatograms of **Mal-Pip-ValCit** and **Mal-Pip-GlyGly**......................... S13

Figure S12: Release of **OsiNHMe** over 3 h during cathepsin B cleavage assay. S14

Figure S13: Cathepsin B cleavage assay of **Mal-Pip-ValCit** and **Mal-Pip-GlyGly** S15

Figure S14: Cell viability data assessed by MTT assay in Caki-1 cells. S116

Figure S15: Western blot of intracellular cathepsin B in different cell lines. S116

Figure S16: Albumin binding of **Mal-Pip-ValCit** and **Mal-Pip-GlyGly**. S177

Figure S17: Cathepsin B cleavage assay of the HSA-Mal-Pip-ValCit conjugate. S117

Figure S18: Free Cys^{34} thiol content of HSA in the presence of **Mal-Pip-GlyGly**. S188

Figure S19: Body weights of the H1650 xenograft in vivo experiment. S199

Figure S20: Histology of H1650 tumors from the in vivo experiment. S199

^{1}H- and ^{13}C NMR spectra. S20–S40

HPLC runs of final compounds. S41–S42

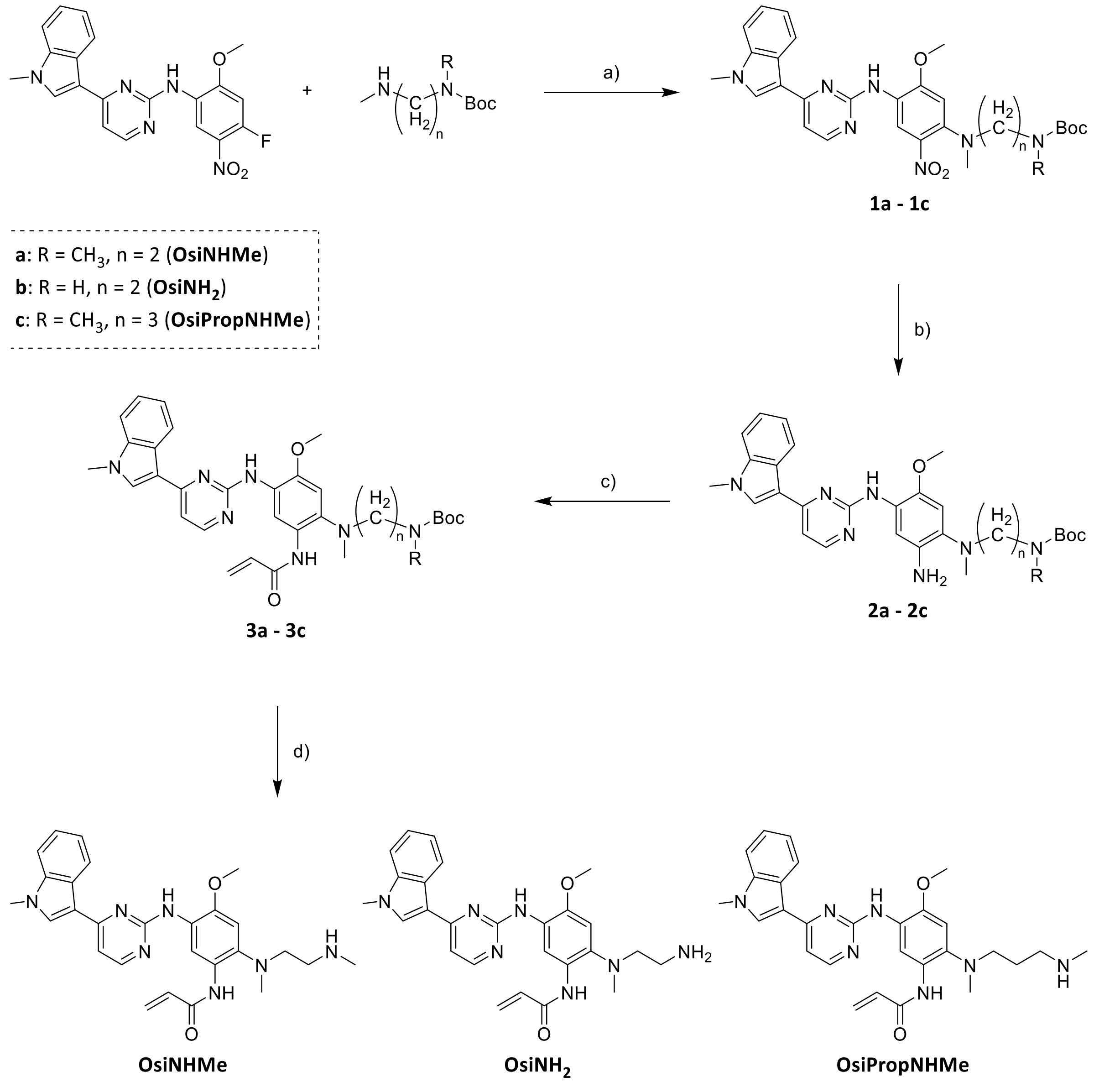

Scheme S1: Synthesis of the osimertinib derivatives **OsiNHMe**, **OsiNH₂** and **OsiPropNHMe**. Reaction conditions: a) K_2CO_3 in DMF, 100°C (yield 1a: 95%, yield 1b: 100%, yield 1c: 95%); b) Fe, NH_4Cl in EtOH/H_2O, 100°C (yield 2a: 91%, yield 2b: 92%, yield 2c: 89%); c) acryloyl chloride, DIPEA in DCM, –80°C (yield 3a: 74%, yield 3b: 87%, yield 3c: 84%); d) TFA in DCM, room temperature (yield **OsiNHMe**: 69%, yield **OsiNH₂**: 75%, yield **OsiPropNHMe**: 64%).

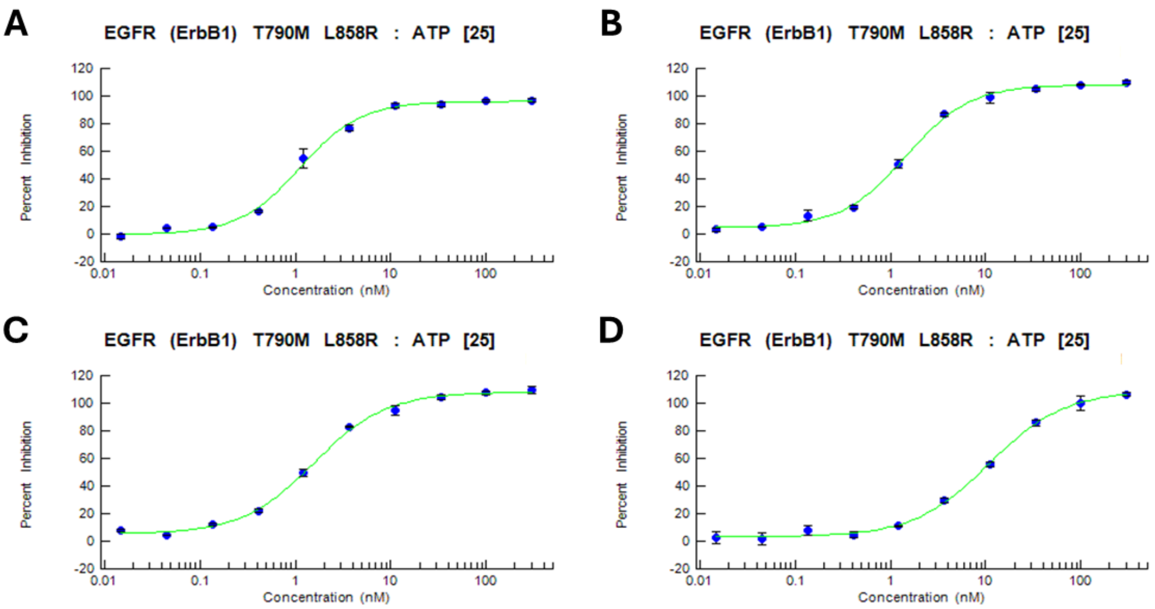

Figure S1: Cell-free IC$_{50}$ measurements of **A**) osimertinib (IC$_{50}$ = 1.11 nM), **B**) **OsiNHMe** (IC$_{50}$ = 1.45 nM), **C**) **OsiNH$_2$** (IC$_{50}$ = 1.56 nM) and **D**) **OsiPropNHMe** (IC$_{50}$ = 10.8 nM) on double-mutant EGFR (L858R/T790M), provided by a commercial vendor (Thermo Fisher).

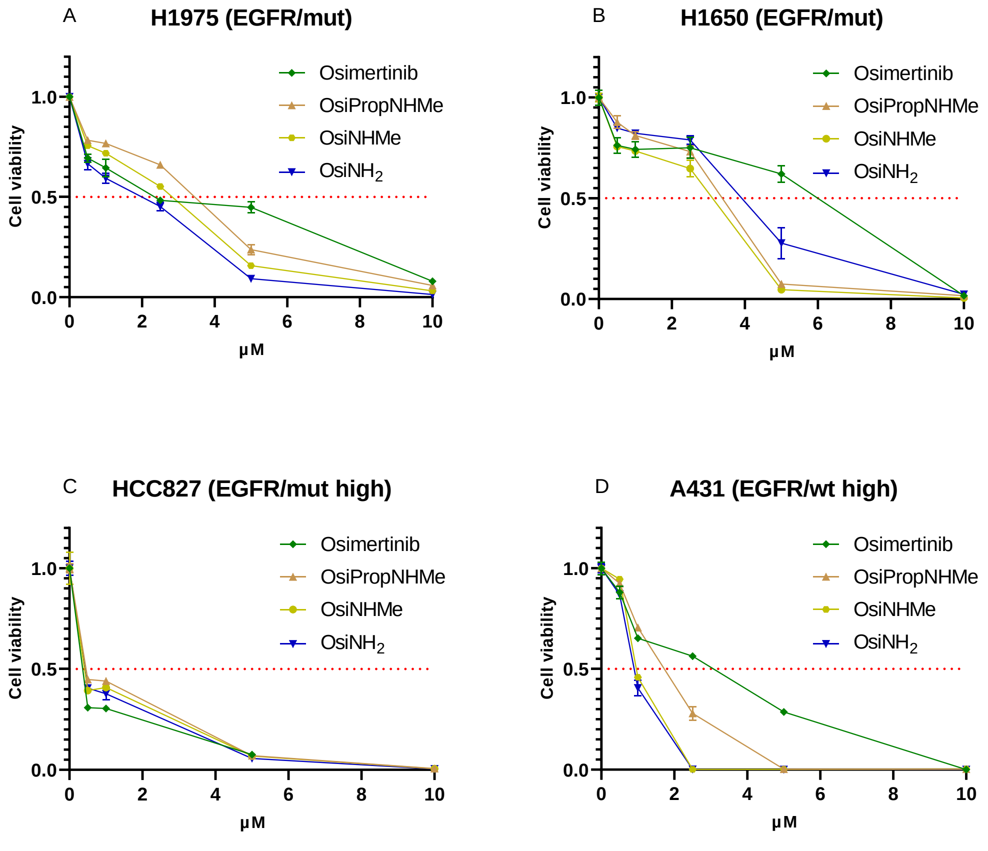

Figure S2: Cell viability of the indicated cell lines assessed by MTT assay after 72 h of incubation with the indicated EGFR inhibitors. Drug response curves were normalized to untreated control cells. Results are reported as mean ± SEM.

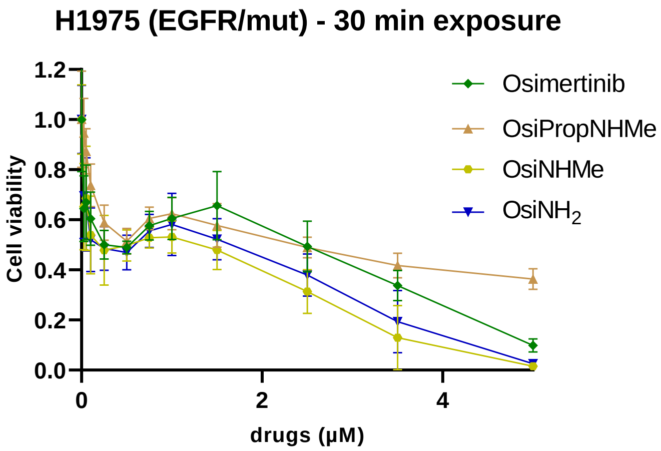

Figure S3: Cell viability of osimertinib, **OsiPropNHMe**, **OsiNHMe** and **OsiNH₂** in H1975 cells, assessed after short-term (30 min) exposure with MTT solution after 72 h of incubation with the indicated EGFR inhibitors. Drug response curves were normalized to untreated control cells. Results are reported as mean ± SEM.

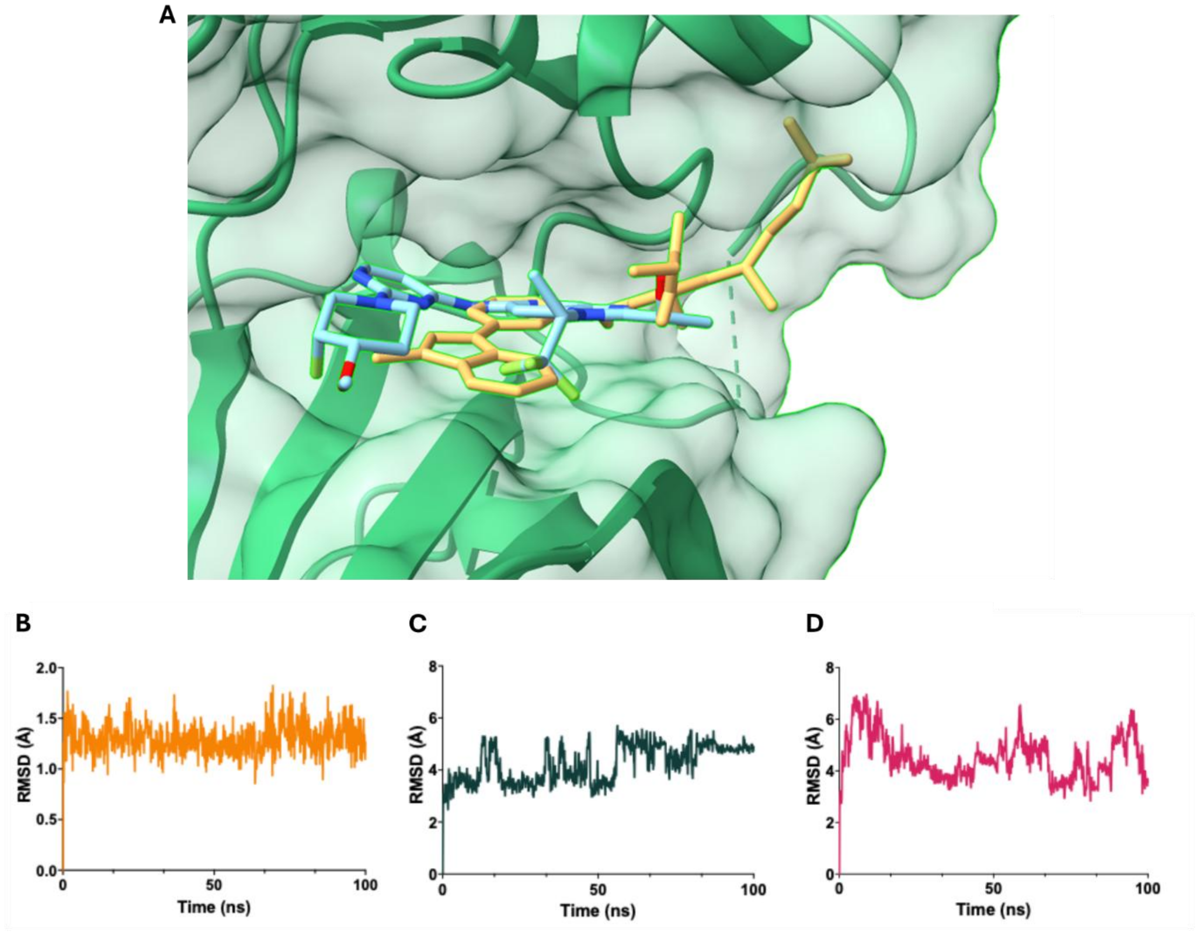

Figure S4: **A**) *In silico* docking studies: 3D representation of mutant EGFR (PDBid: 5CAS, green surface) interacting with the original co-crystalized ligand (in cyan) and osimertinib (in gold). **B**–**D**) Molecular Dynamics (MD): Root-mean-square deviation (RMSD, Å) vs simulation time (ns) for the MD simulations for **B**) **OsiNHMe**, **C**) **Mal-Pip-ValCit**, and **D**) **Mal-Pip-GlyGly**.

A

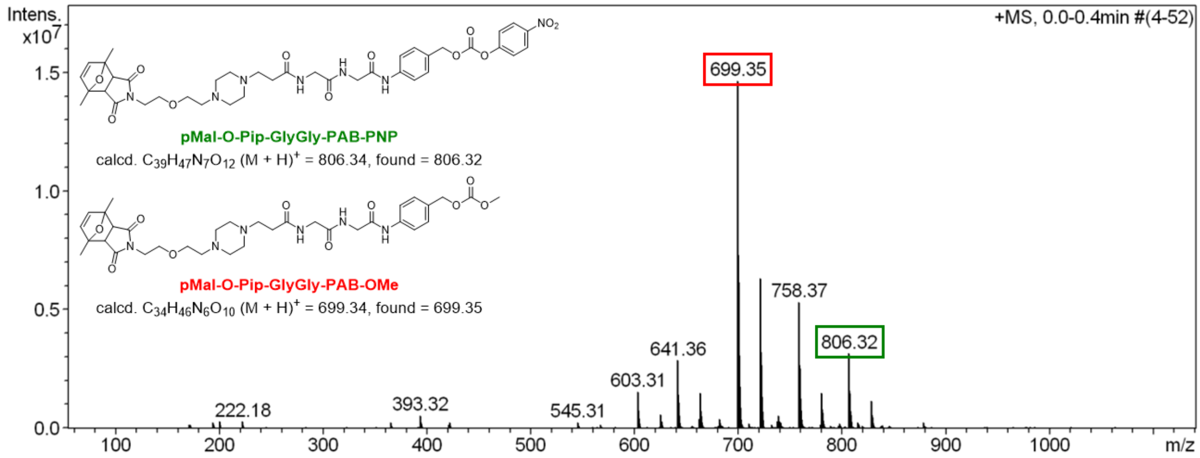

B

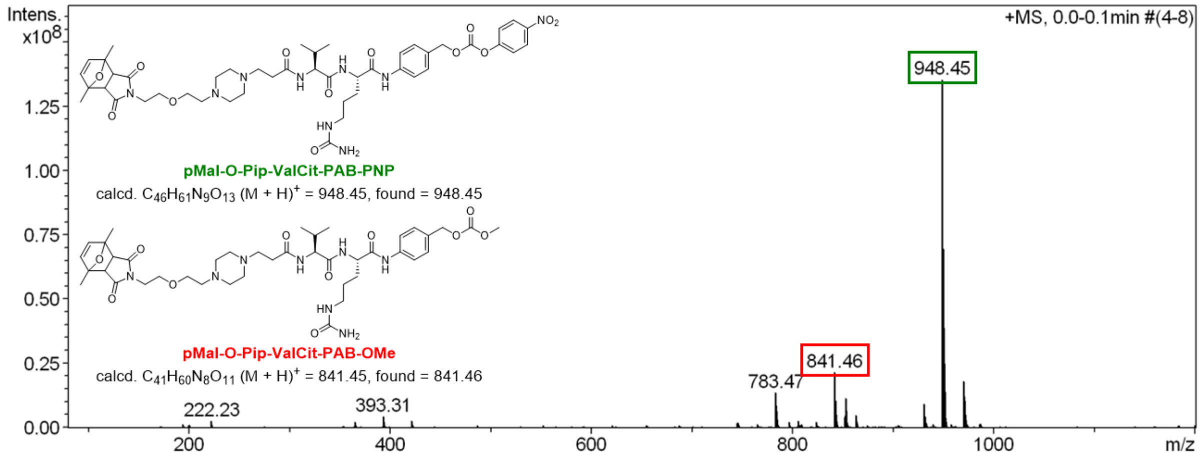

Figure S5: Mass spectra after the column chromatographic purification of pMal-O-Pip-GlyGly-PAB-PNP and pMal-O-Pip-ValCit-PAB-PNP after solvent removal (DCM/MeOH). The product masses (green) were observed as well as the methyl carbonate ester side product (red), which forms by nucleophilic substitution of the *para*-nitrophenyl carbonate active ester with methanol. Observed masses: **A**) pMal-O-Pip-GlyGly-PAB-PNP, calcd. $C_{39}H_{47}N_7O_{12}$ $(M + H)^+$ = 806.34, found = 806.32; pMal-O-Pip-GlyGly-PAB-OMe, calcd. $C_{34}H_{46}N_6O_{10}$ $(M + H)^+$ = 699.34, found = 699.35. **B**) pMal-O-Pip-ValCit-PAB-PNP, calcd. $C_{46}H_{61}N_9O_{13}$ $(M + H)^+$ = 948.45, found = 948.45; pMal-O-Pip-ValCit-PAB-OMe, calcd. $C_{41}H_{60}N_8O_{11}$ $(M + H)^+$ = 841.45, found = 841.46.

A

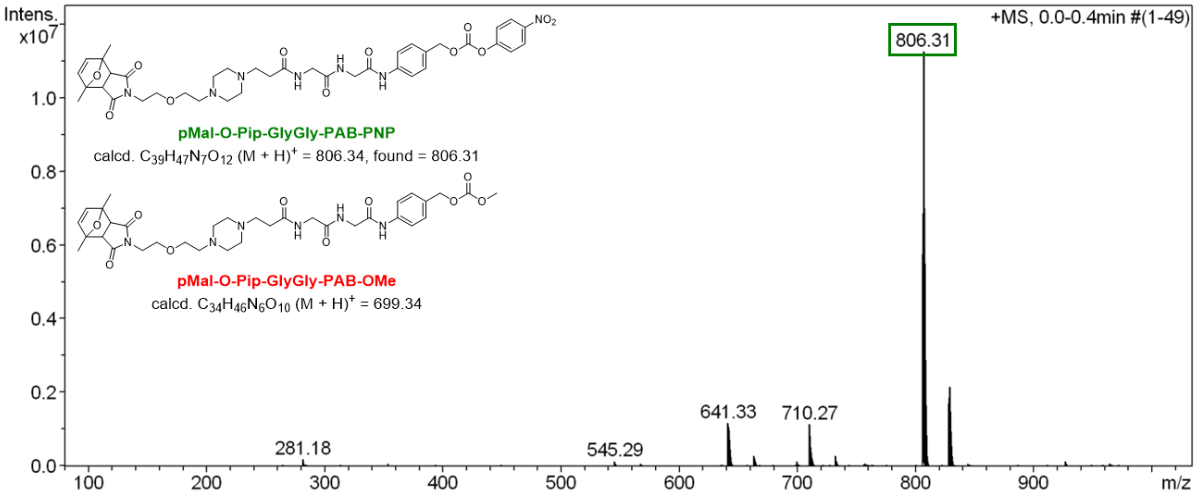

B

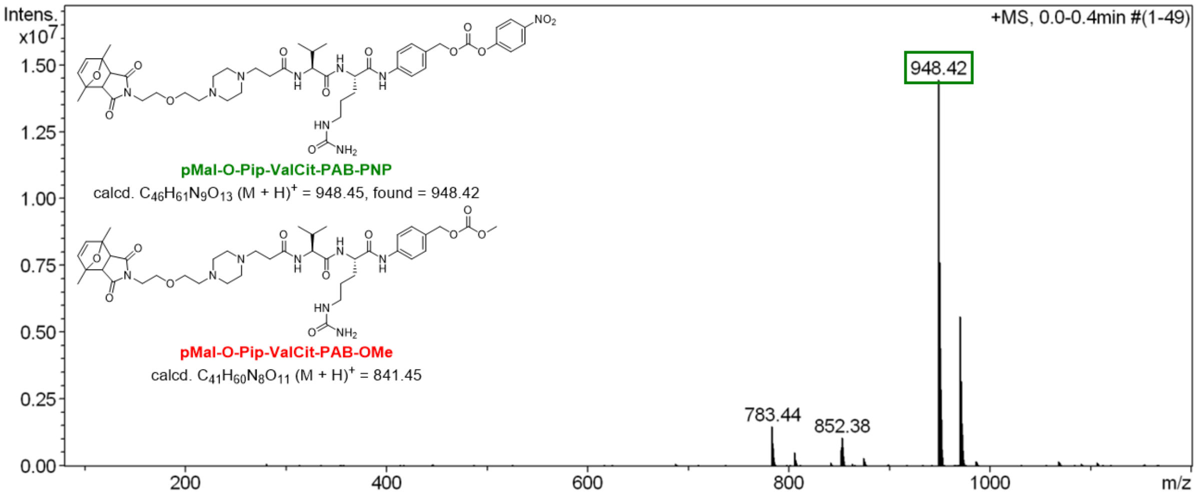

Figure S6: Mass spectra after the column chromatographic purification of pMal-O-Pip-GlyGly-PAB-PNP and pMal-O-Pip-ValCit-PAB-PNP after solvent removal (DCM/MeOH) with the addition of toluene. The product masses (green) were observed, but no methyl carbonate side product. Observed masses: **A**) pMal-O-Pip-GlyGly-PAB-PNP, calcd. $C_{39}H_{47}N_7O_{12}$ $(M + H)^+$ = 806.34, found = 806.31. **B**) pMal-O-Pip-ValCit-PAB-PNP, calcd. $C_{46}H_{61}N_9O_{13}$ $(M + H)^+$ = 948.45, found = 948.42.

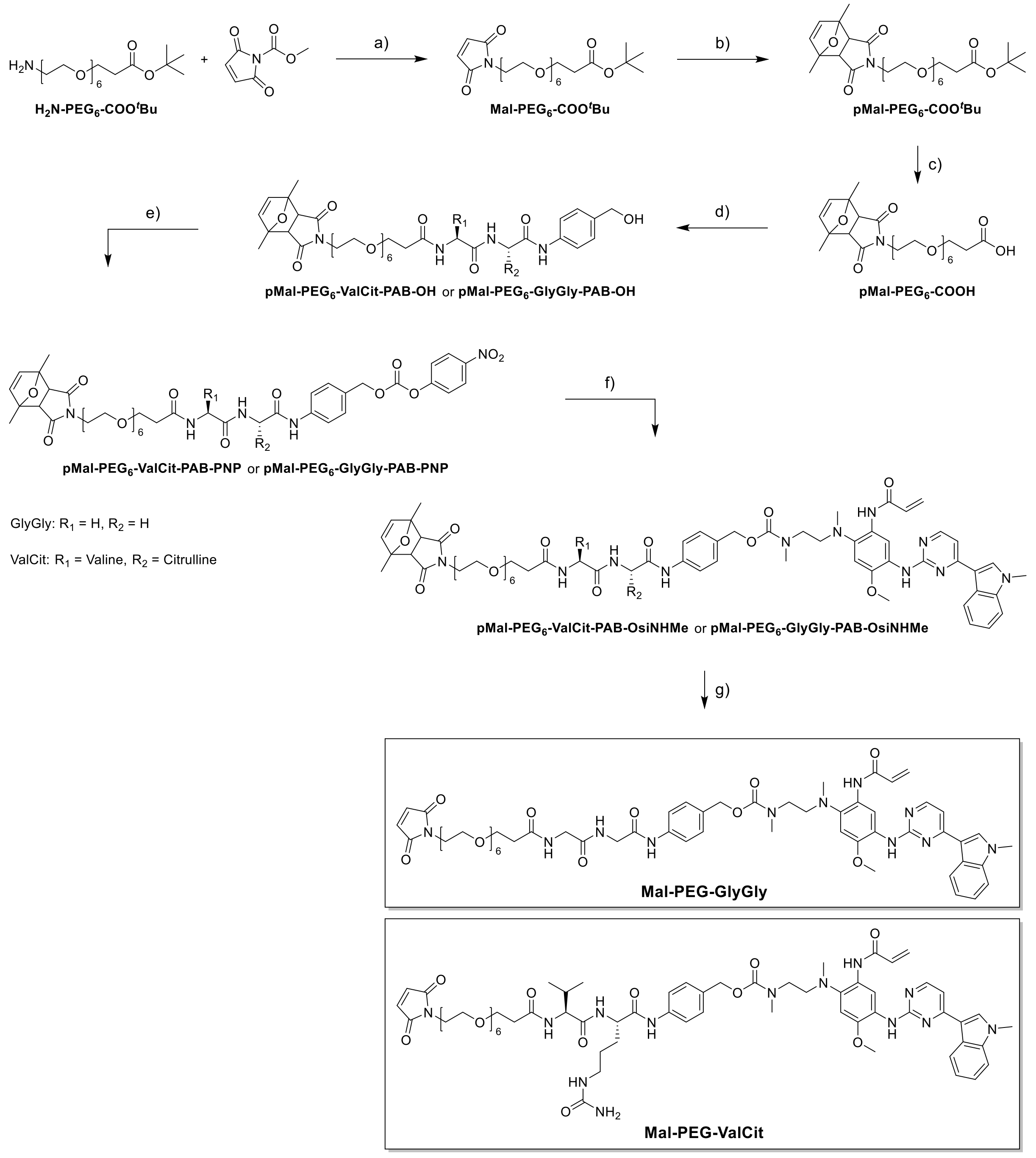

Scheme S2: Synthesis of the target compounds **Mal-PEG-GlyGly** and **Mal-PEG-ValCit**. Reaction conditions: a) tetrabutylammonium bisulfate, NEt_3 in 1:2 $CHCl_3$ and saturated $NaHCO_3$ solution, 0°C → room temperature (yield: 89%); b) 2,5-dimethylfuran in MeCN, 60°C (yield: 93%); c) TFA in DCM, room temperature (yield: 83%); d) H_2N-GlyGly-PAB-OH (1) or H_2N-ValCit-PAB-OH (2), EDC·HCl, HOBt, NEt_3 in dry DMF, room temperature (yield 1: 81%, yield 2: 87%; e) bis(4-nitrophenyl) carbonate, DIPEA in dry DMF, room temperature (yield 1: 89%, yield 2: 79%); f) **OsiNHMe**, DIPEA in dry DMF, room temperature (yield 1: 95%, yield 2: 71%); g) DMSO, 90°C (yield **Mal-PEG-GlyGly**: 66%, yield **Mal-PEG-ValCit**: 63%).

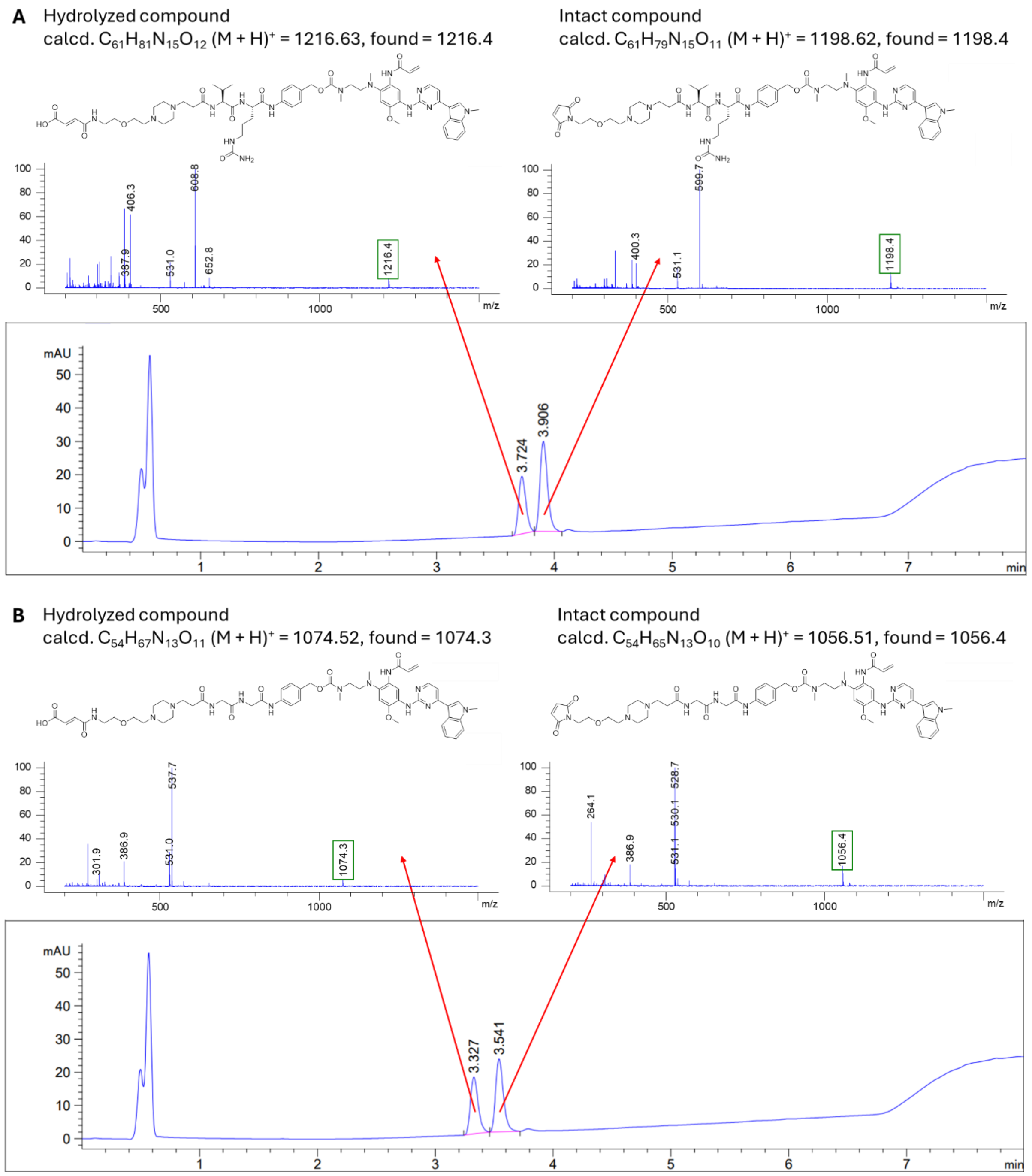

Figure S7: HPLC-MS chromatograms of **Mal-Pip-ValCit** and **Mal-Pip-GlyGly** during the stability measurements in PB at pH 7.4 (Figure S8). The mass spectra belonging to the individual UV-peaks are indicated with red arrows. **A**) **Mal-Pip-ValCit** elutes at 3.906 min and the maleamic acid hydrolysis product at 3.724 min. **B**) **Mal-Pip-GlyGly** elutes at 3.541 min and the maleamic acid hydrolysis product at 3.327 min.

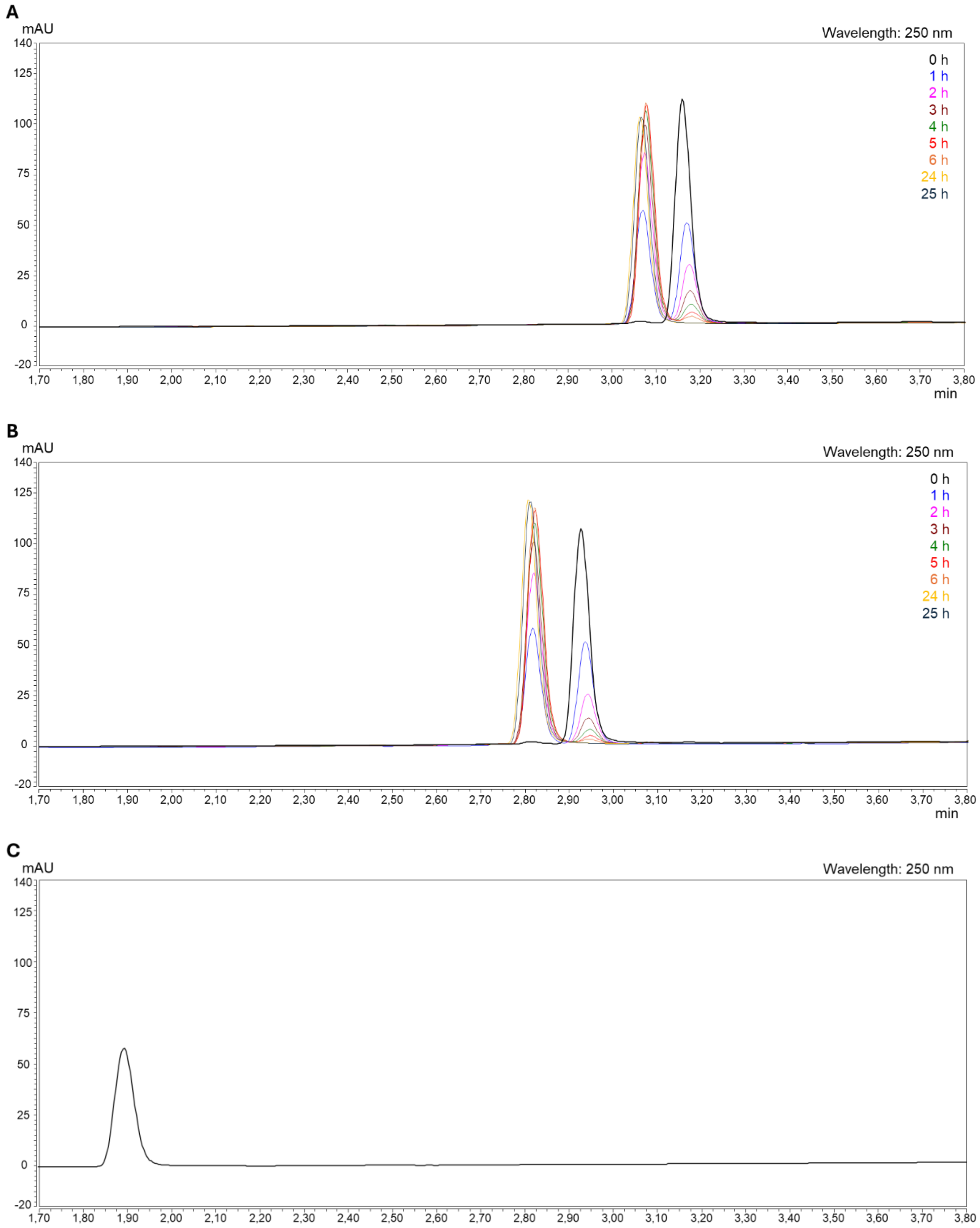

Figure S8: Stability measurements of **Mal-Pip-ValCit** and **Mal-Pip-GlyGly** *via* HPLC. Both compounds were incubated at 10 µM in 10 mM PB at pH 7.4 and 37°C and were stable >25 h, apart from maleimide hydrolysis (Figure S7). **A**) **Mal-Pip-ValCit** elutes at 3.17 min and the maleamic acid hydrolysis product at 3.07 min. **B**) **Mal-Pip-GlyGly** elutes at 2.93 min and the maleamic acid hydrolysis product at 2.81 min. **C**) Reference spectrum of **OsiNHMe**, eluting at 1.89 min, showing that the TKI is not being released >25 h.

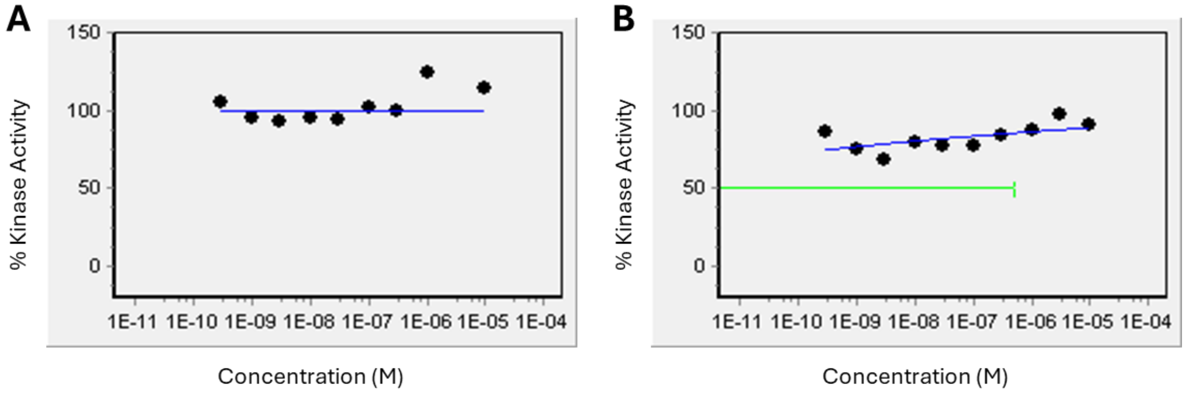

Figure S9: Cell-free IC_{50} measurements of **A**) **Mal-Pip-GlyGly** and **B**) **Mal-Pip-ValCit** on double-mutated (T790M/L858R) EGFR, provided by a commercial vendor (Reaction Biology). IC_{50} values could not be determined within the tested concentration range due to the low EGFR-inhibitory potential of both compounds.

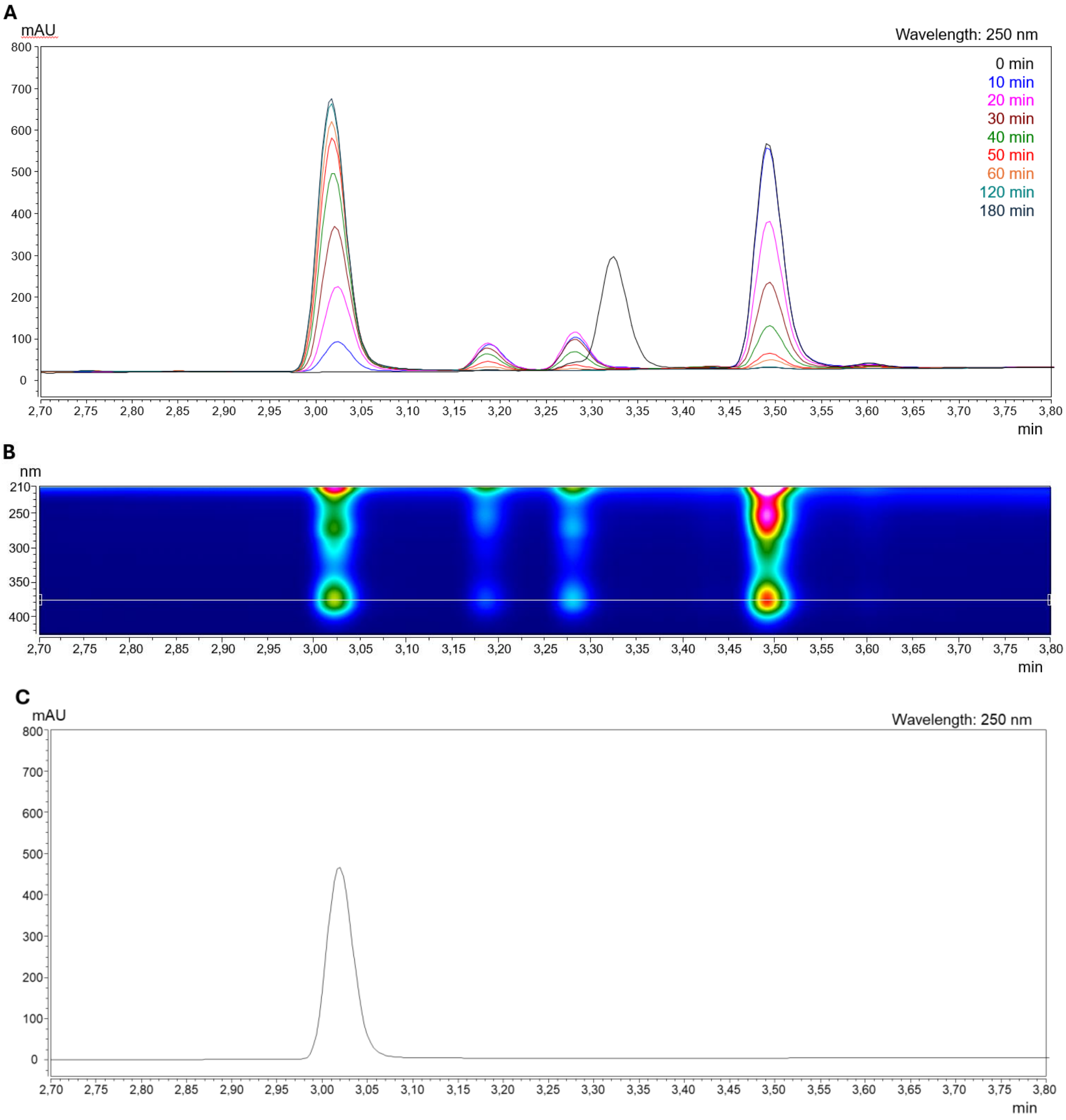

Figure S10: Cathepsin B cleavage assay of **Mal-Pip-ValCit** to analyze the release of **OsiNHMe**, monitored *via* HPLC. Depicted are the chromatograms in the range of 2.70–3.80 min. Cathepsin B was incubated with DTT and EDTA in water at room temperature. The mixture was then diluted into 1.5 mL NaOAc buffer containing 1 mM EDTA at pH 5.0, the compound was added and the mixture incubated at 37°C. The final compound concentration was 46 µM. **A**) **Mal-Pip-ValCit** with enzyme: **Mal-Pip-ValCit** eluted at 3.32 min and the DTT-adduct at 3.49 min, which formed by Michael addition of the DTT-thiol to maleimide (see Figure S11). The release of **OsiNHMe** was indicated by the growing peak at 3.02 min and was complete after ~1.5 h (see Figure S12). During this time, intermediate compounds at 3.19 and 3.28 min were observed, bearing **OsiNHMe** as shown by the UV spectrum of the respective chromatograms. **B**) UV-Vis spectrum of the 20 min timepoint chromatogram in "**A**": all peaks show the characteristic local maximum of **OsiNHMe** at 375 nm, indicated by the white horizontal line. **C**) Reference spectrum of **OsiNHMe**.

A Intact compound
calcd. $C_{61}H_{79}N_{15}O_{11}$ $(M + H)^+$ = 1198.62, found = 1198.4

DTT-Pip-ValCit adduct
calcd. $C_{126}H_{168}N_{30}O_{24}S_2$ $(M + 4H)^{4+}$ = 638.32, found = 638.5

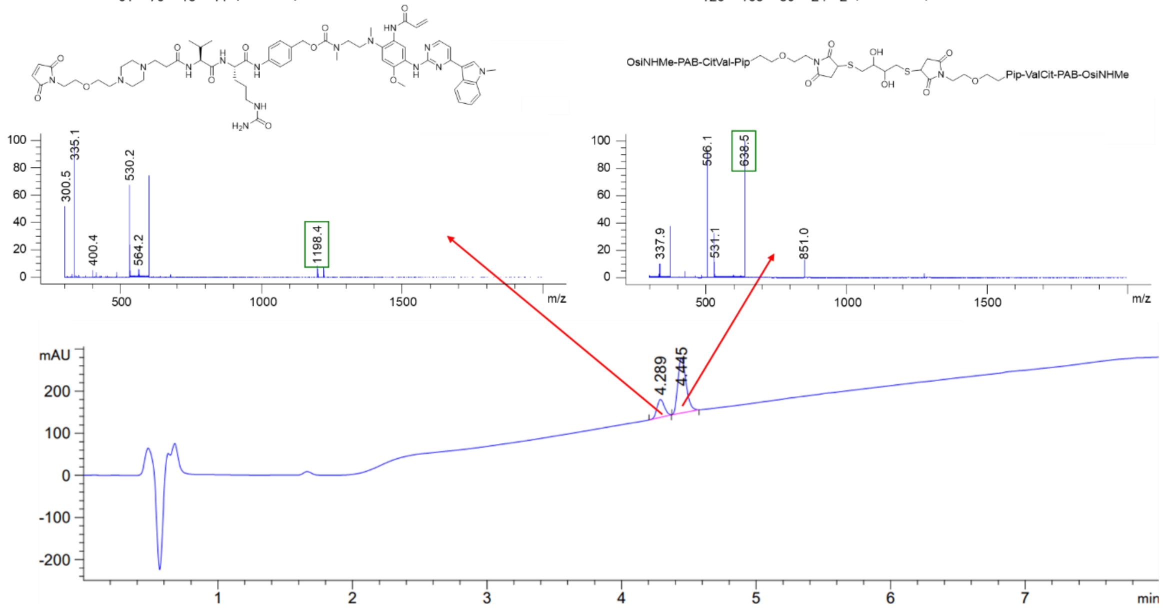

B Intact compound
calcd. $C_{54}H_{65}N_{13}O_{10}$ $(M + H)^+$ = 1056.51, found = 1056.3

DTT-Pip-GlyGly adduct
calcd. $C_{112}H_{140}N_{26}O_{22}S_2$ $(M + 4H)^{4+}$ = 567.26, found = 567.4

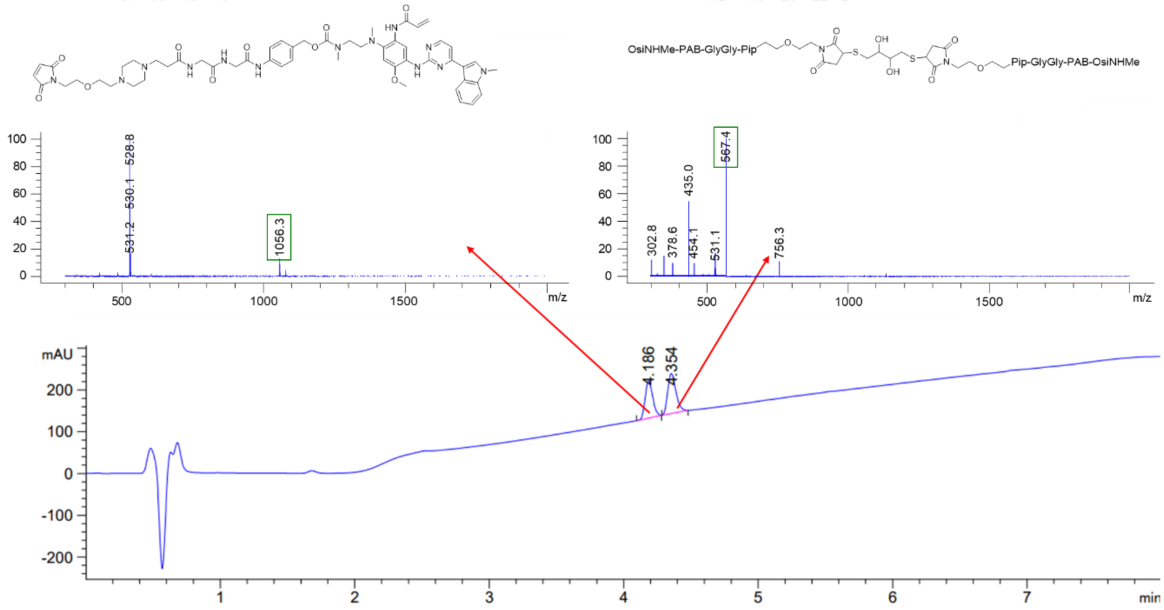

Figure S11: HPLC-MS chromatograms of **Mal-Pip-ValCit** and **Mal-Pip-GlyGly** in the presence of DTT and EDTA (without cathepsin B). For both compounds, the DTT-adduct was observed only as the $(M + 4H)^{4+}$ ion. The mass spectra belonging to the individual UV-peaks are indicated with red arrows. **A**) **Mal-Pip-ValCit** elutes at 4.289 min and the DTT-adduct at 4.445 min. **B**) **Mal-Pip-GlyGly** elutes at 4.186 min and the DTT-adduct at 4.354 min.

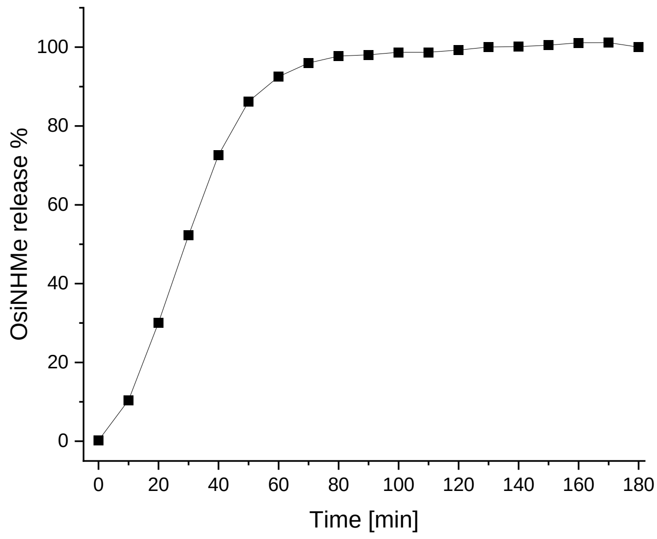

Figure S12: Release of **OsiNHMe** over 3 h, quantified from the cleavage assay of **Mal-Pip-ValCit** with cathepsin B (Figure S10A). The timepoints were measured *via* HPLC and the peak areas of **OsiNHMe** were normalized to that in the last run (180 min). The drug release was complete after ~1.5 h.

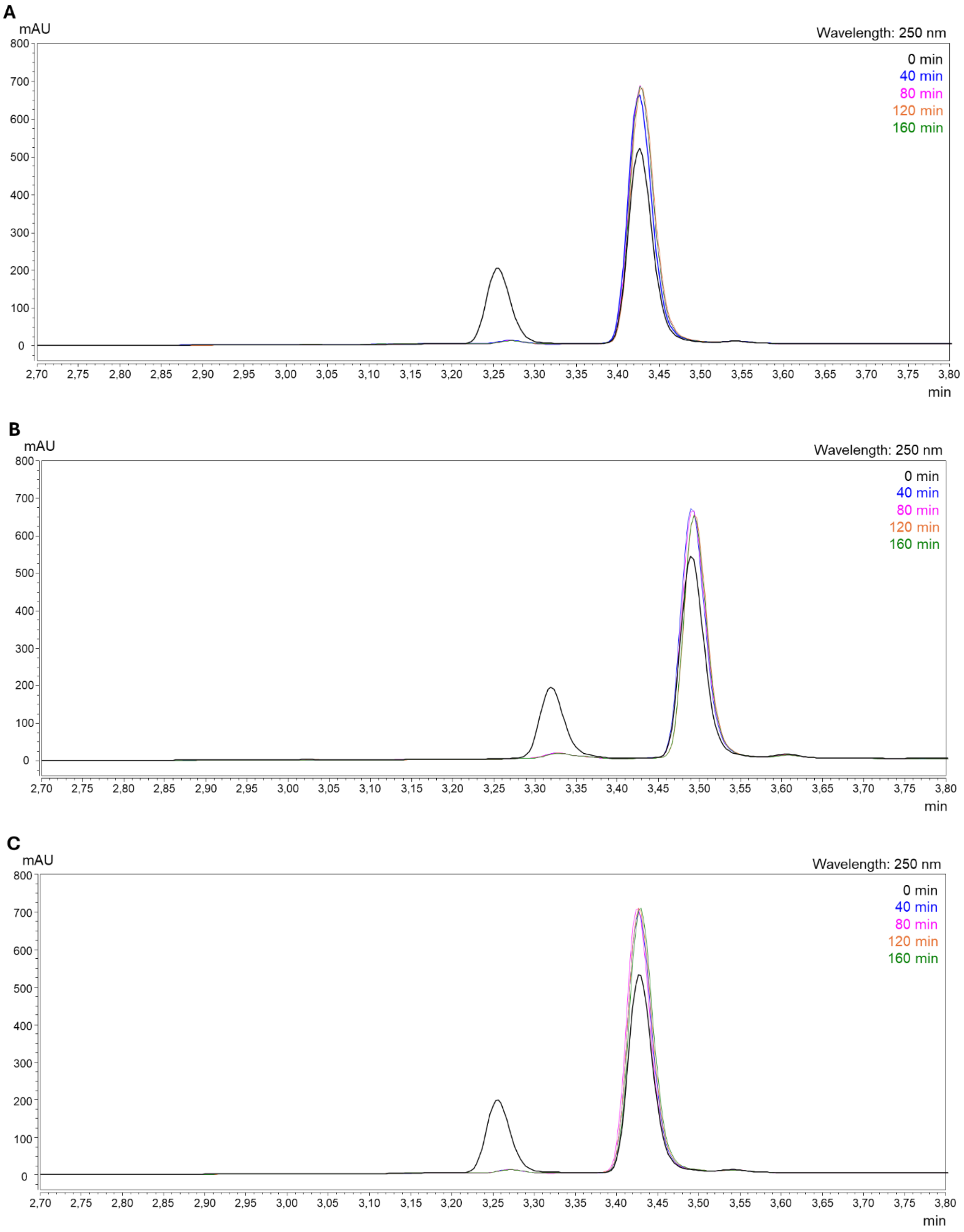

Figure S13: Cleavage assay of **Mal-Pip-GlyGly** with Cathepsin B and as control samples **Mal-Pip-ValCit** and **Mal-Pip-GlyGly** without the enzyme, monitored *via* HPLC. Depicted are the chromatograms in the range of 2.70–3.80 min. Cathepsin B or water was incubated with DTT and EDTA in water at room temperature. The mixture was then diluted into 1.5 mL NaOAc buffer containing 1 mM EDTA at pH 5.0, the compounds were added and the mixtures incubated at 37°C. The final compound concentration was 46 µM. Both compounds reacted quantitatively to form the DTT adduct and no release of **OsiNHMe** was detected over 160 min (**OsiNHMe** elutes at 3.02 min, Figure 9C). **A**) **Mal-Pip-GlyGly** with enzyme: **Mal-Pip-GlyGly** eluted at 3.26 min and the DTT-adduct at 3.43 min. **B**) **Mal-Pip-ValCit** without enzyme: **Mal-Pip-ValCit** eluted at 3.32 min and the DTT-adduct at 3.49 min. **C**) **Mal-Pip-GlyGly** without enzyme: **Mal-Pip-GlyGly** eluted 3.26 min and the DTT-adduct at 3.43 min.

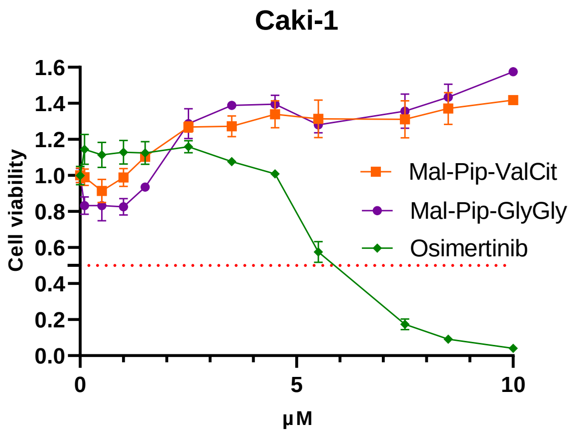

Figure S14: Activity of **Mal-Pip-ValCit**, **Mal-Pip-GlyGly** and osimertinib in Caki-1 cells after 72 h of drug treatment. Cell viability was assessed using MTT assays. Drug response curves were normalized to untreated control cells. Data were pooled from three independent experimental replicates, each yielding comparable results. Data are expressed as mean ± SEM.

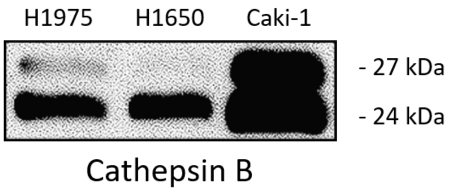

Figure S15: Intracellular cathepsin B expression on the indicated cancer cell lines analyzed by Western blotting.

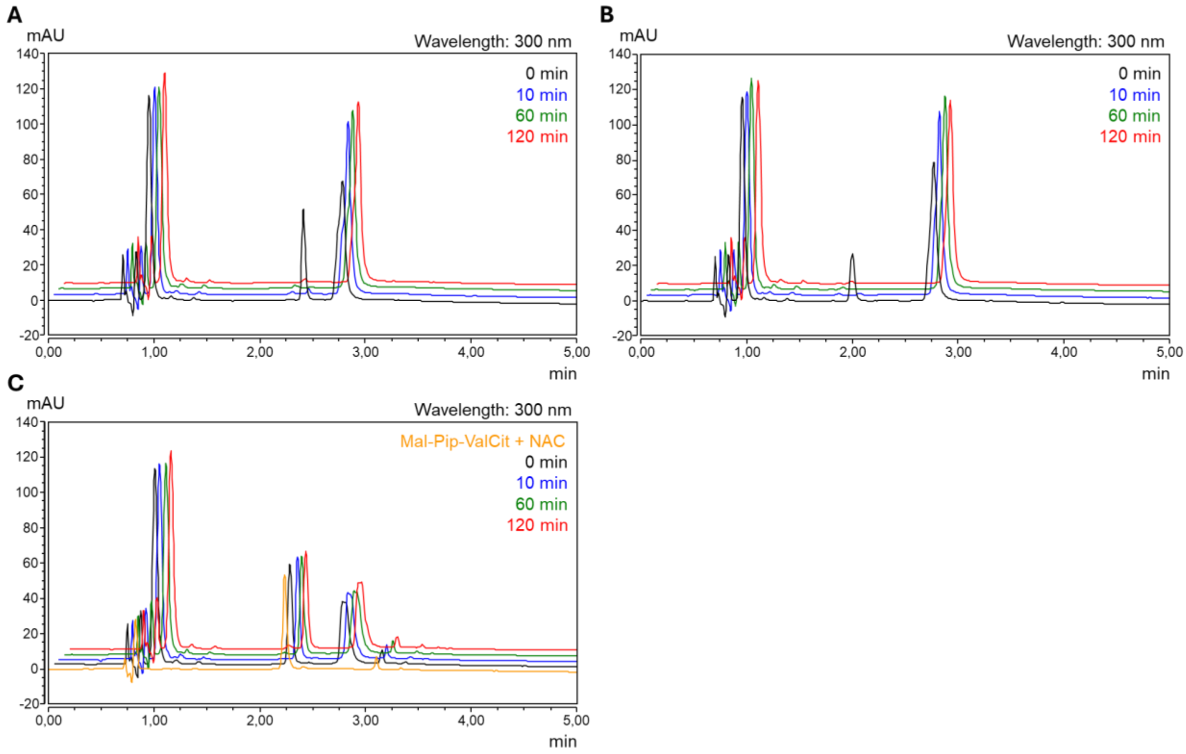

Figure S16: Albumin binding of the target compounds **Mal-Pip-ValCit** and **Mal-Pip-GlyGly**. Depicted are the chromatograms in the range of 0–5 min. For “**A**” and “**B**”, compound stocks (2.5 mM in water with 25% DMSO) were diluted 1:50 into ~300 µM HSA (prepared from Albunorm®, in 150 mM PB, pH = 7.4) to get a final compound concentration of 50 µM. The samples were incubated at 37°C and analyzed *via* HPLC. **A**) **Mal-Pip-ValCit** elutes at 2.41 min and albumin between ~2.7–3.0 min. **B**) **Mal-Pip-GlyGly** elutes at 2.00 min and albumin between ~2.7–3.0 min. In both cases, after 10 min already >95% were bound. The small peaks at 2.26 min in “**A**” and 1.82 min in “**B**”, presumably correspond to the hydrolyzed maleimide. **C**) 10 µL of *N*-acetylcysteine (NAC; 5 mM in 150 mM PB, pH = 7.4) and 100 µL **Mal-Pip-ValCit** (0.5 mM in H_2O, containing 5% DMSO) were combined. The NAC-Mal-Pip-ValCit adduct elutes at 2.23 min. After conjugation, the solution was incubated with ~300 µM HSA (prepared from Albunorm®, in 150 mM PB, pH = 7.4) to get a final compound concentration of 50 µM. Albumin elutes between ~2.7–3.0 min. In all spectra, the peak at 0.95 min belongs to tryptophane, which is an additive in Albunorm® solution.

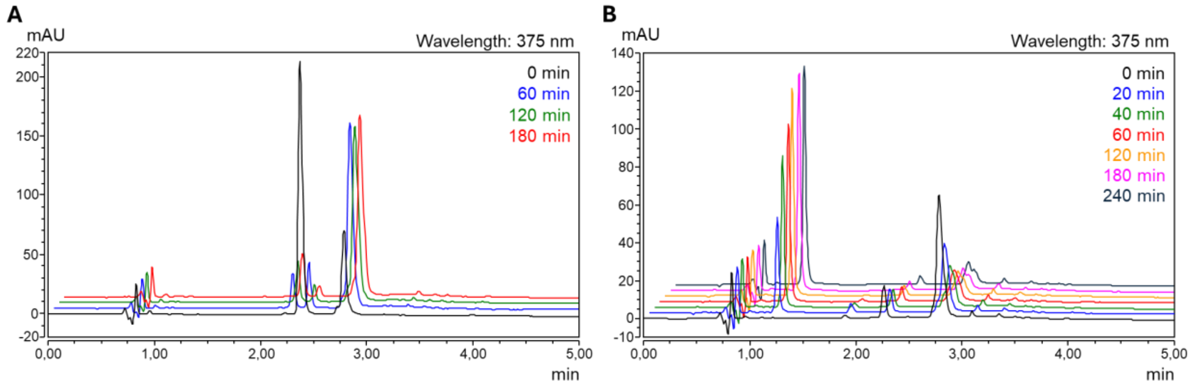

Figure S17: Cathepsin B cleavage assay showing **OsiNHMe** release from the albumin-drug conjugate. **A**) Incubation of 100 µM **Mal-Pip-ValCit** (peak at 2.37 min) with HSA (~300 µM in 10 mM PB, peak between ~2.7–3.0 min) over 3h, until ~85% binding was achieved. **B**) Incubation of the HSA-drug conjugate with cathepsin B to give final conditions of 46 µM drug in ~50 mM NaOAc buffer at pH 5. Free **OsiNHMe** was observed at 1.21 min. In both Figures, the hydrolyzed **Mal-Pip-ValCit** elutes at 2.25 min and albumin between 2.7–3.0 min. The 375 nm wavelength was chosen to easily show all peaks with an EGFR inhibitor unit.

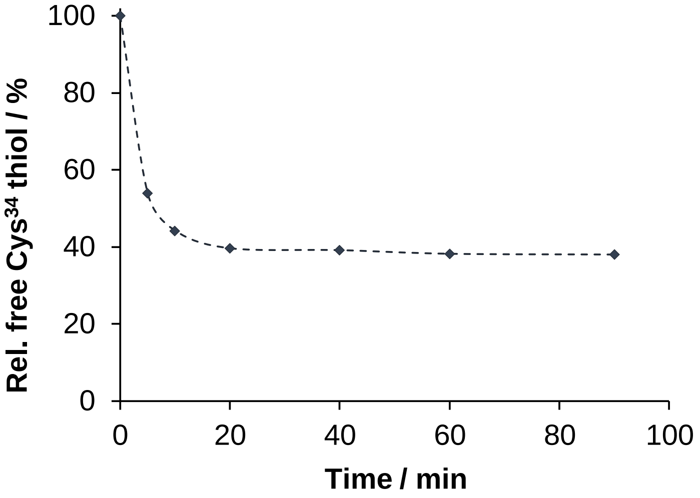

Figure S18: Relative free Cys[34] thiol content of HSA after various incubation times in the presence of 1 eq. **Mal-Pip-GlyGly** [c_{Cys34} = $c_{compound}$ = 6.2 µM; (c_{HSA} = 19 µM); pH = 7.40 (100 mM phosphate buffer), 37°C].

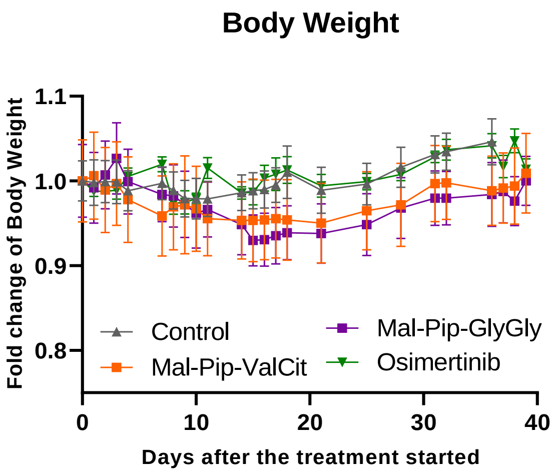

Figure S19: Body weights of the H1650 xenograft *in vivo* experiment treated i.v. twice per week with equimolar doses of **Mal-Pip-ValCit** (83.7 mg/kg), **Mal-Pip-GlyGly** (75.7 mg/kg) or osimertinib mesylate (29.8 mg/kg) for two weeks.

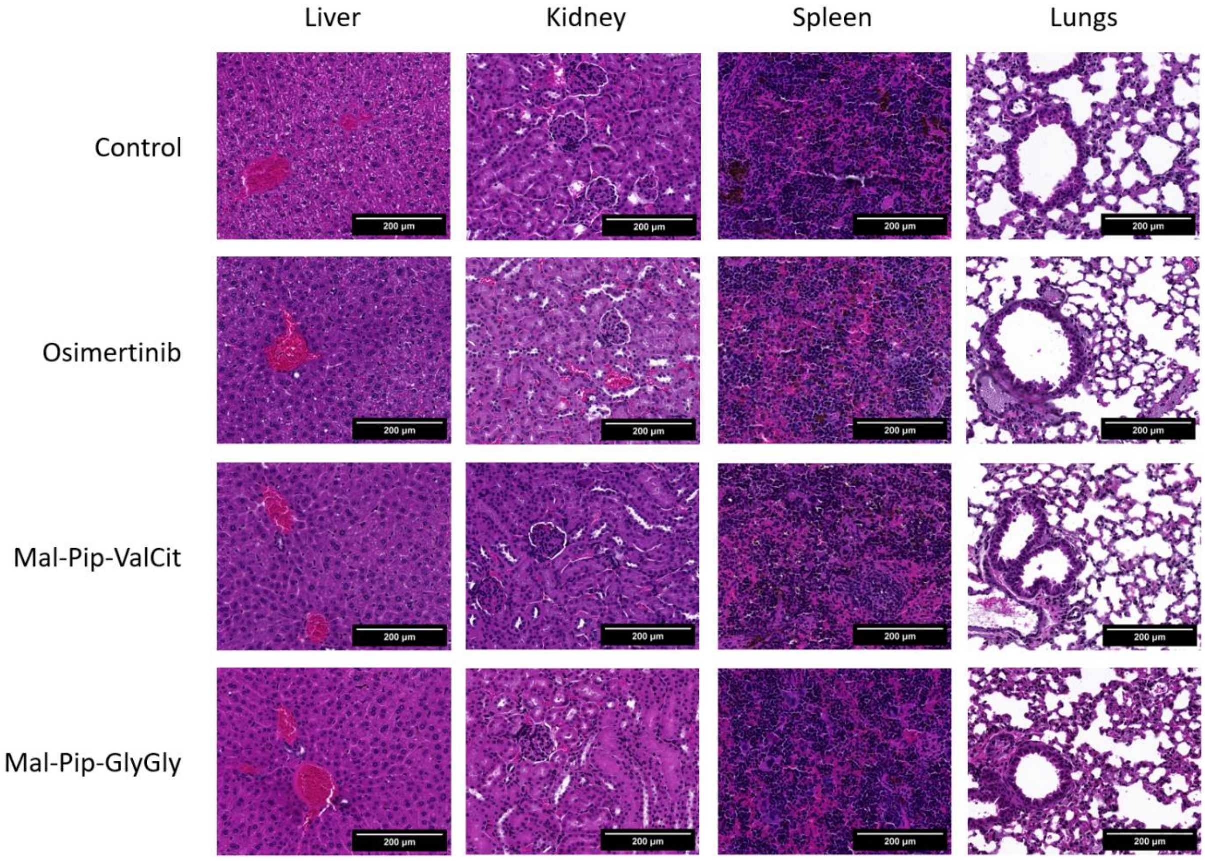

Figure S20: Sample pictures of organs (60x magnification) from the long-term overall survival animal experiment (see main manuscript, Figure 8D/E). Animals were treated two times a week for two weeks. Scale bars = 200 µm.

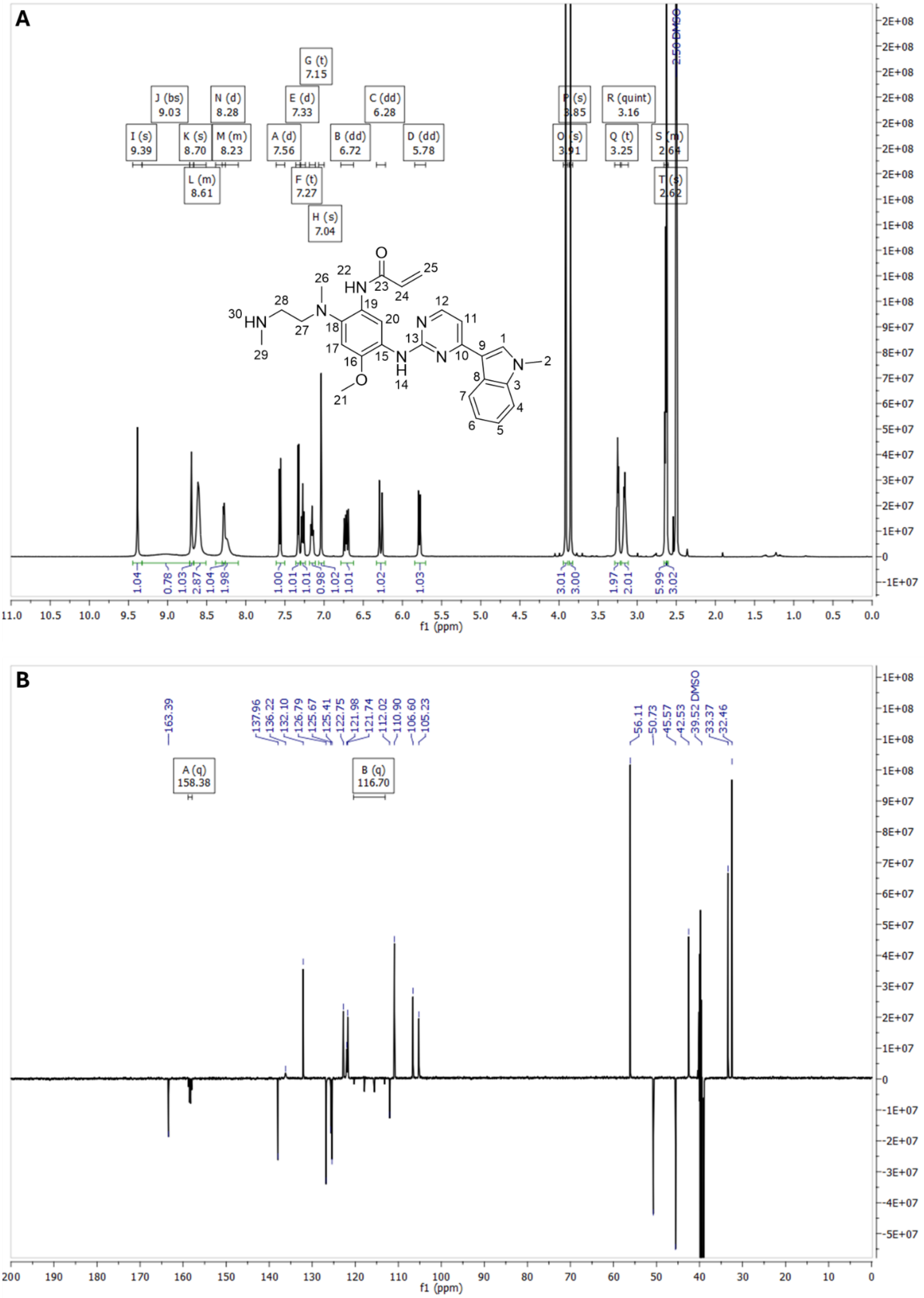

Figure S21: **A**) ^{1}H NMR spectrum, **B**) ^{13}C NMR spectrum and structure of **OsiNHMe**.

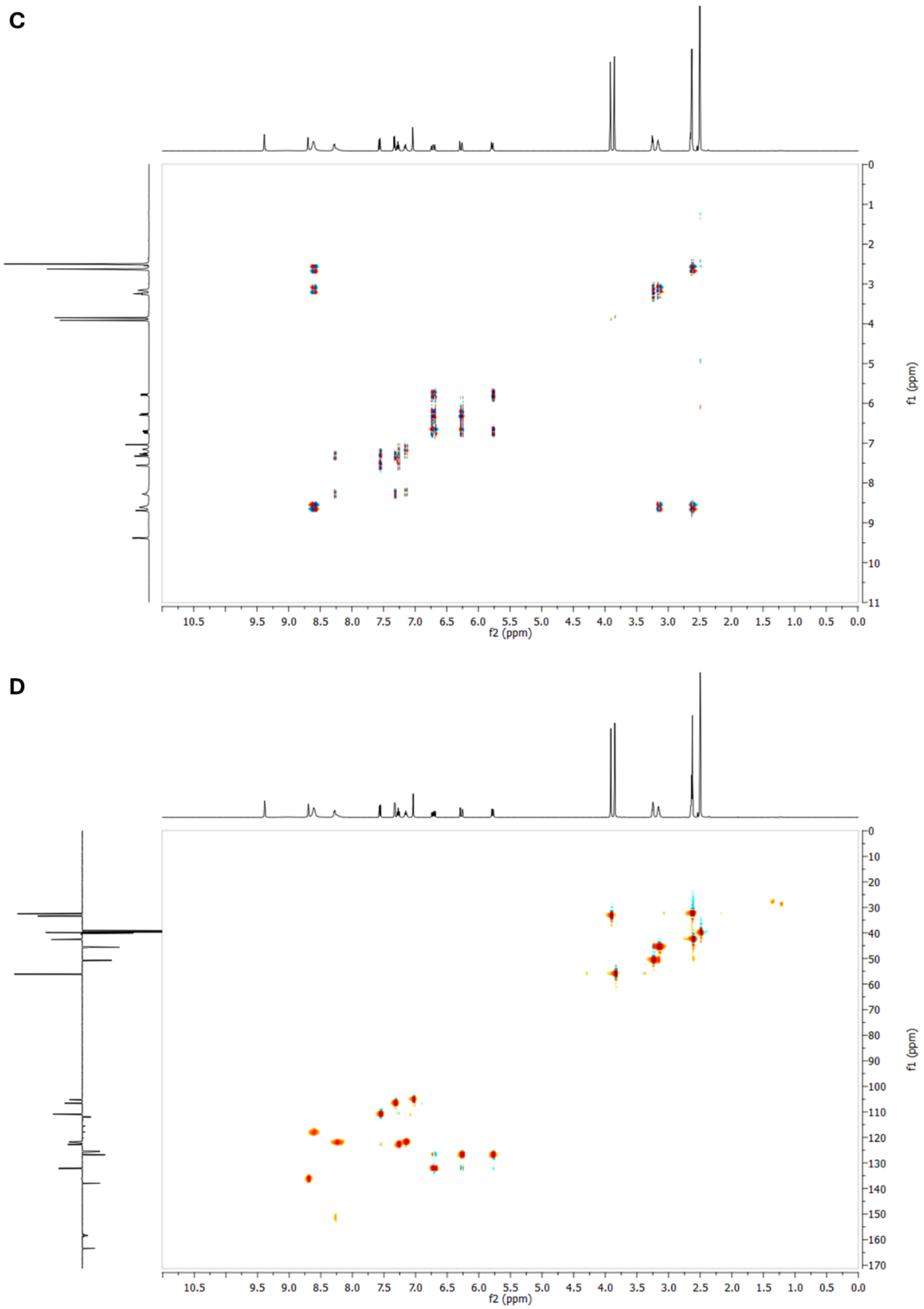

Figure S21 (continued): **C**) COSY NMR spectrum and **D**) HSQC NMR spectrum of **OsiNHMe**.

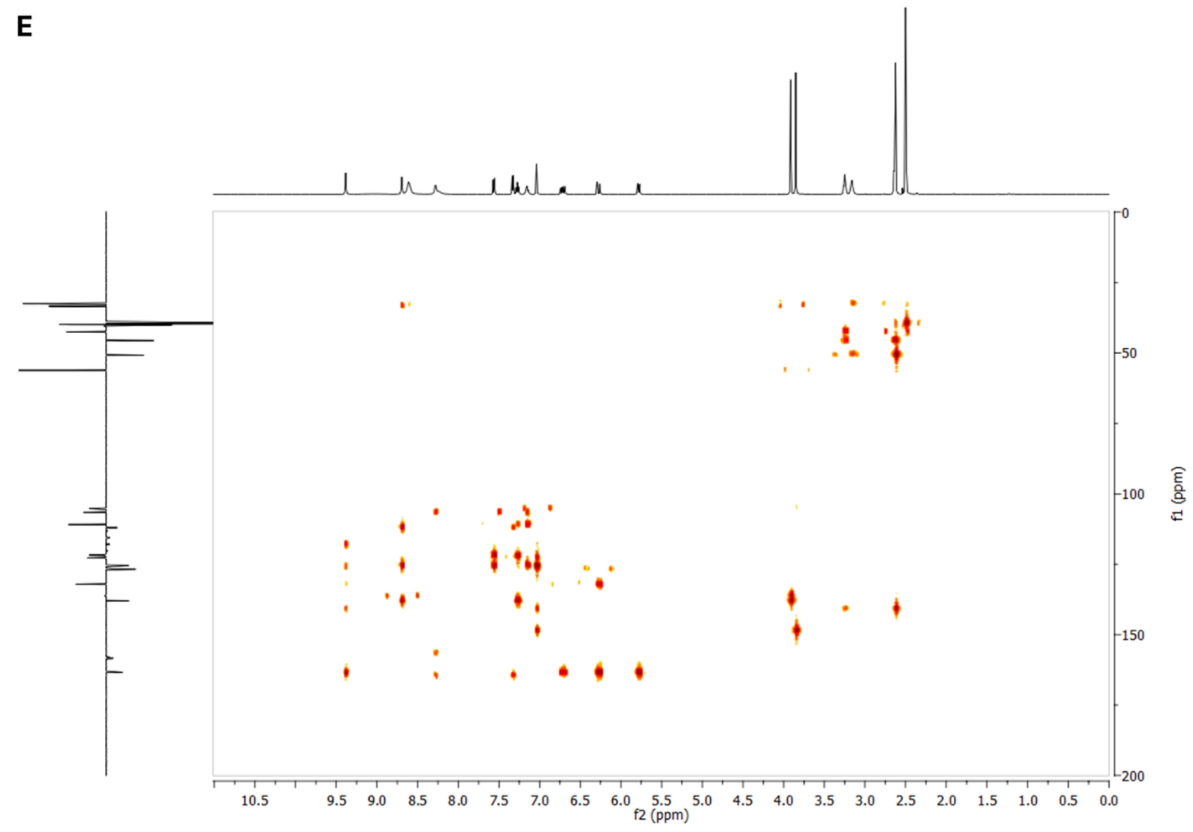

Figure S21 (continued): **E**) HMBC NMR spectrum of **OsiNHMe**.

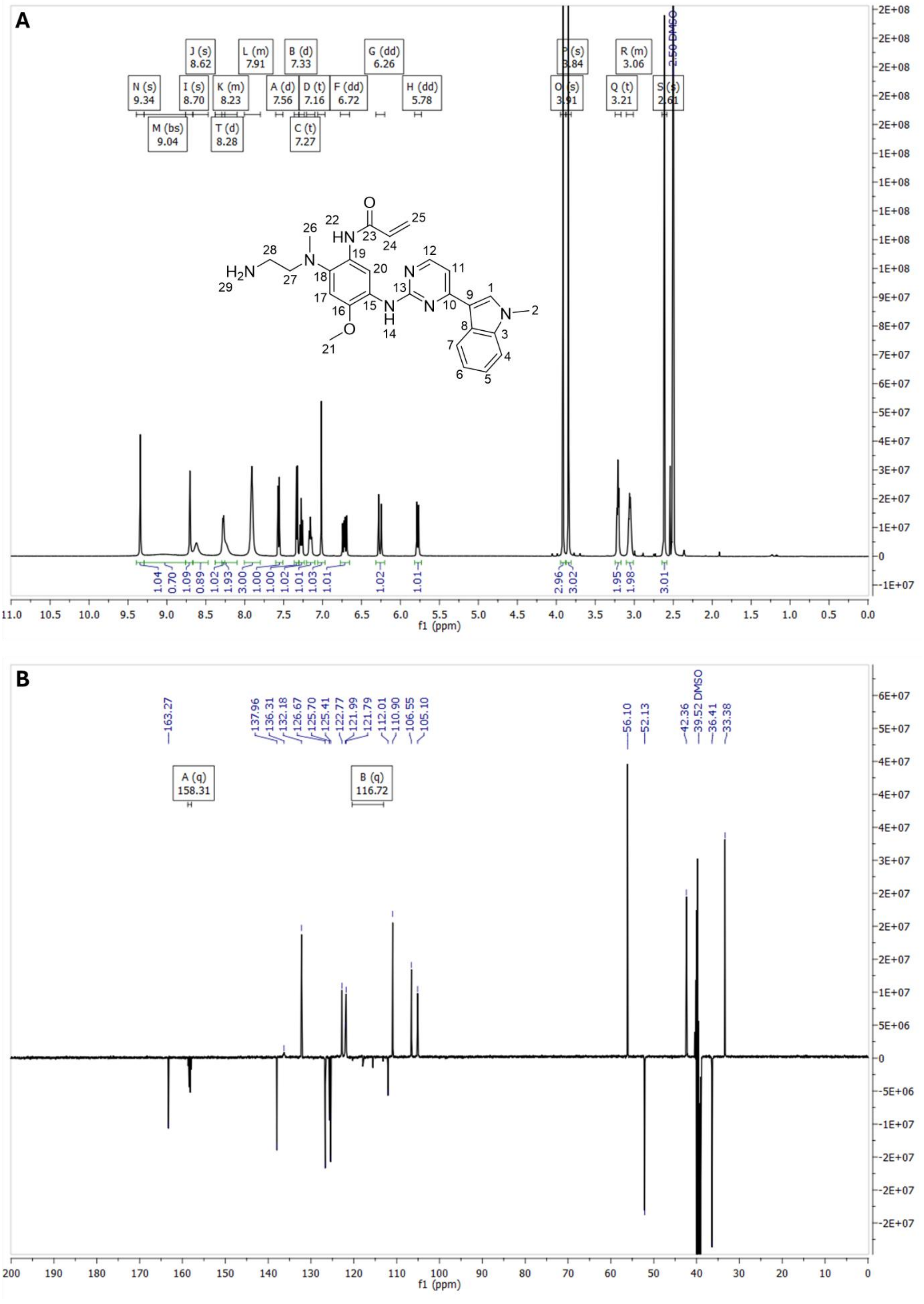

Figure S22: **A**) ^{1}H NMR spectrum, **B**) ^{13}C NMR spectrum and structure of **OsiNH$_2$**.

C

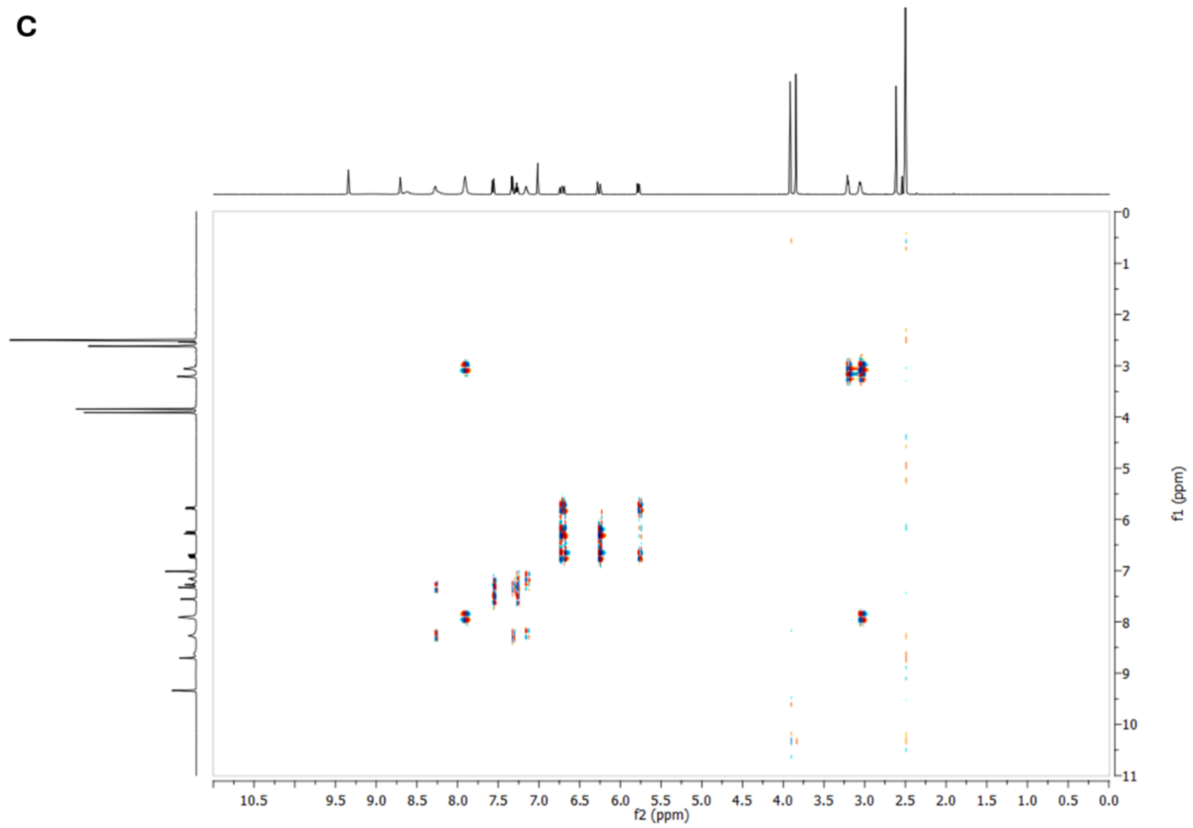

D

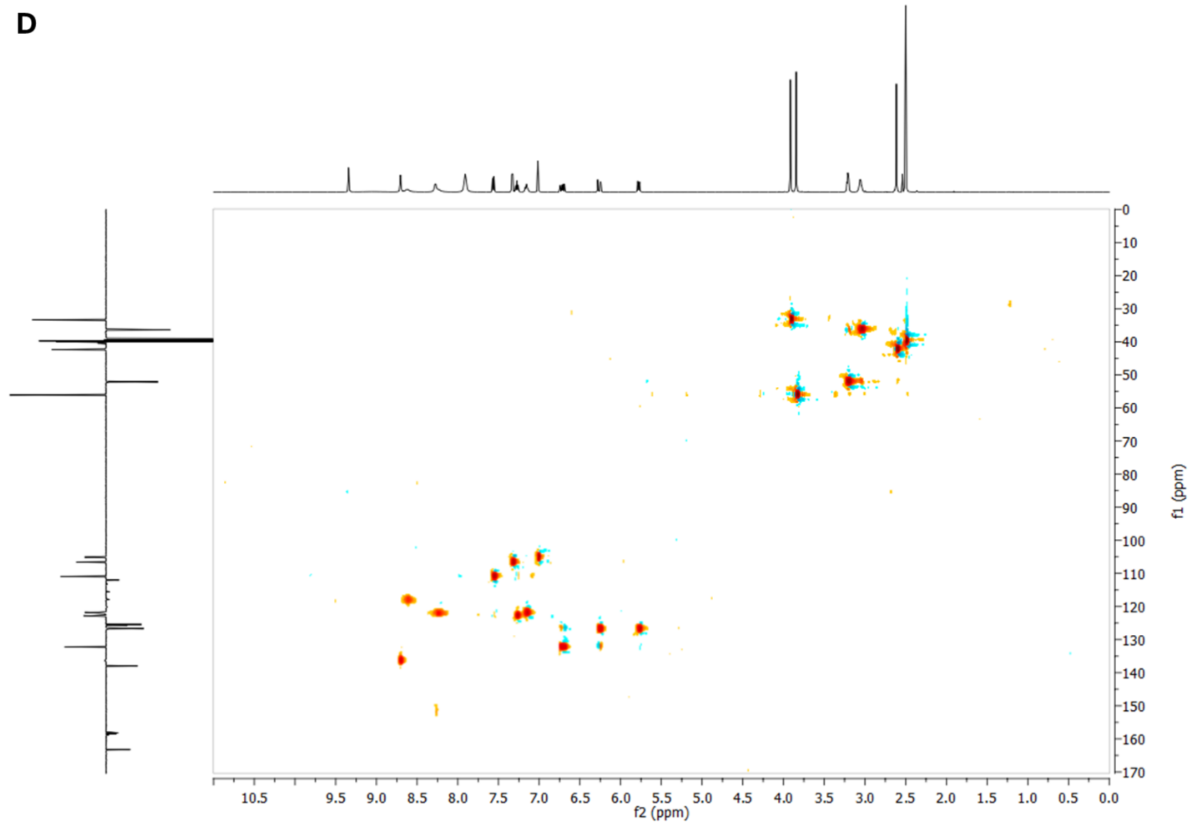

Figure S22 (continued): **C**) COSY NMR spectrum and **D**) HSQC NMR spectrum of **OsiNH$_2$**.

E

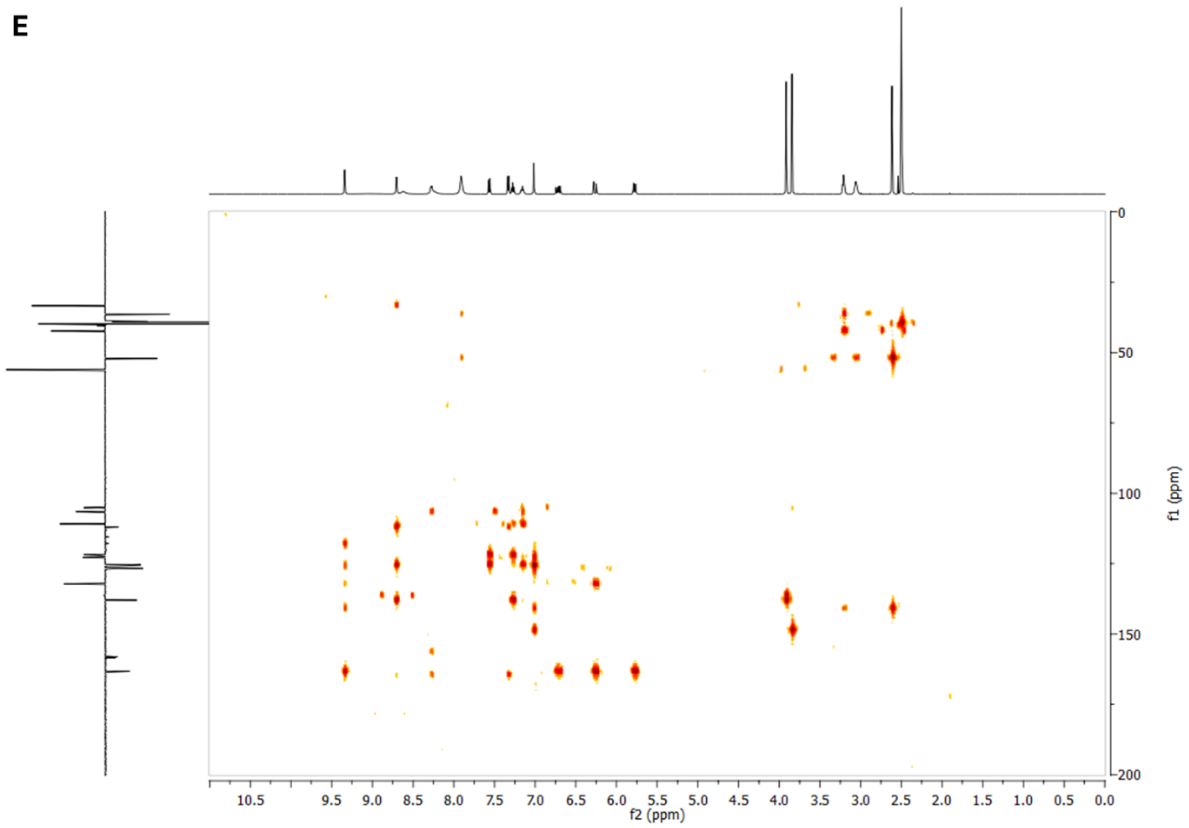

Figure S22 (continued): **E**) HMBC NMR spectrum of **OsiNH$_2$**.

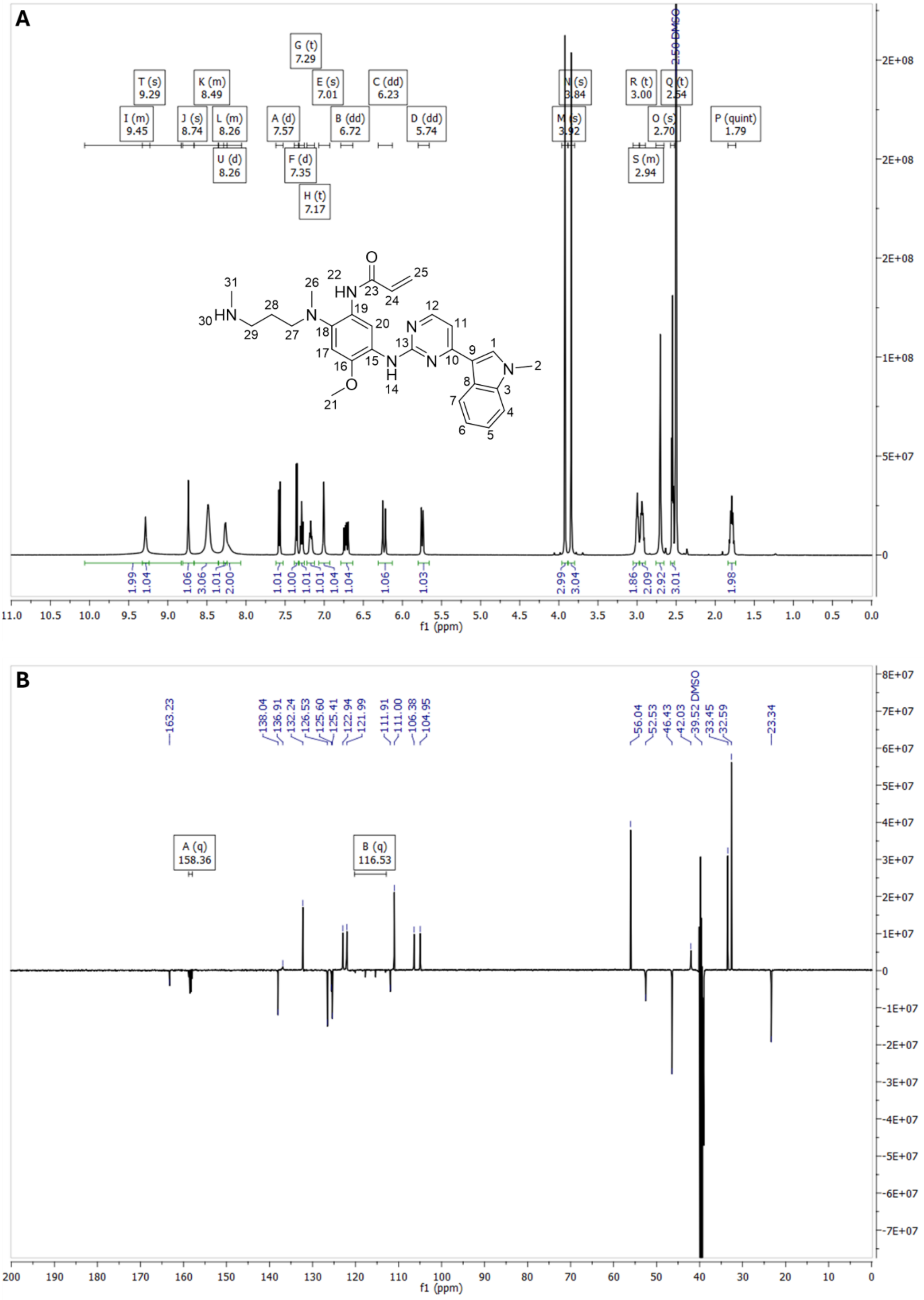

Figure S23: **A**) ^{1}H NMR spectrum, **B**) ^{13}C NMR spectrum and structure of **OsiPropNHMe**.

C

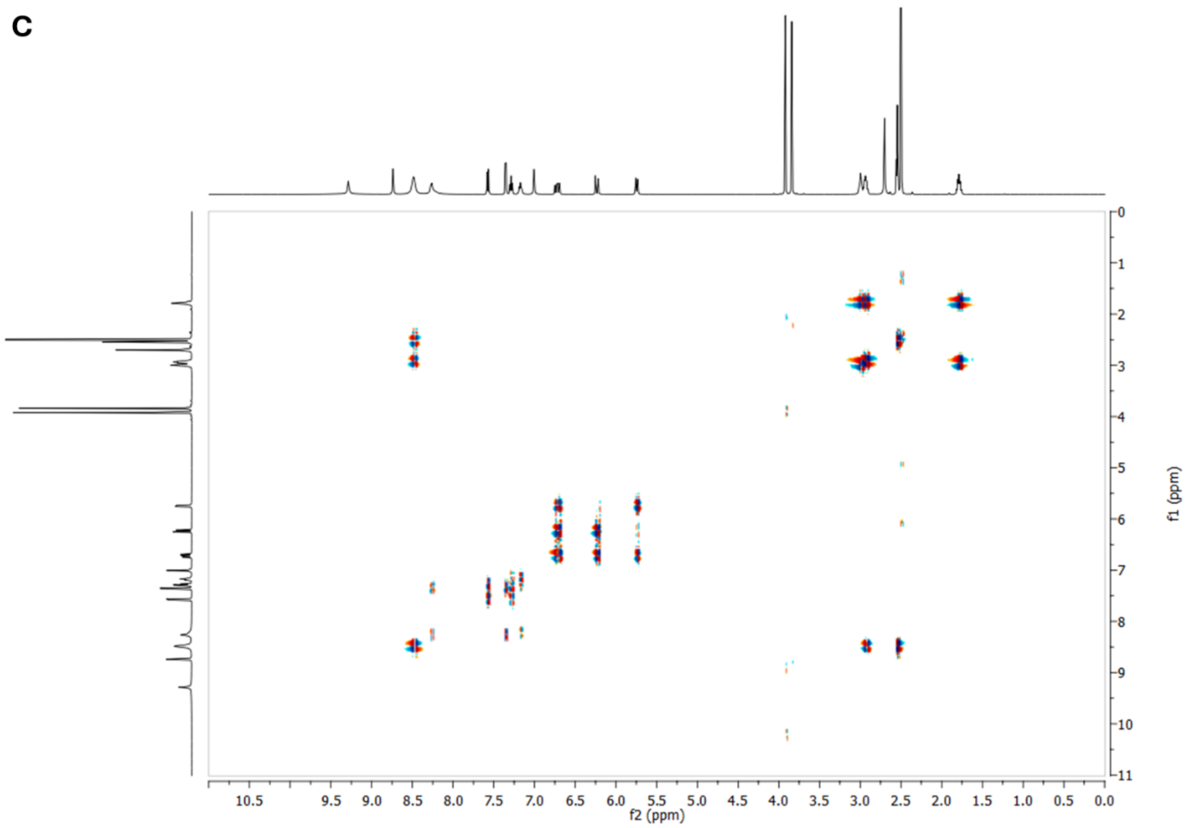

D

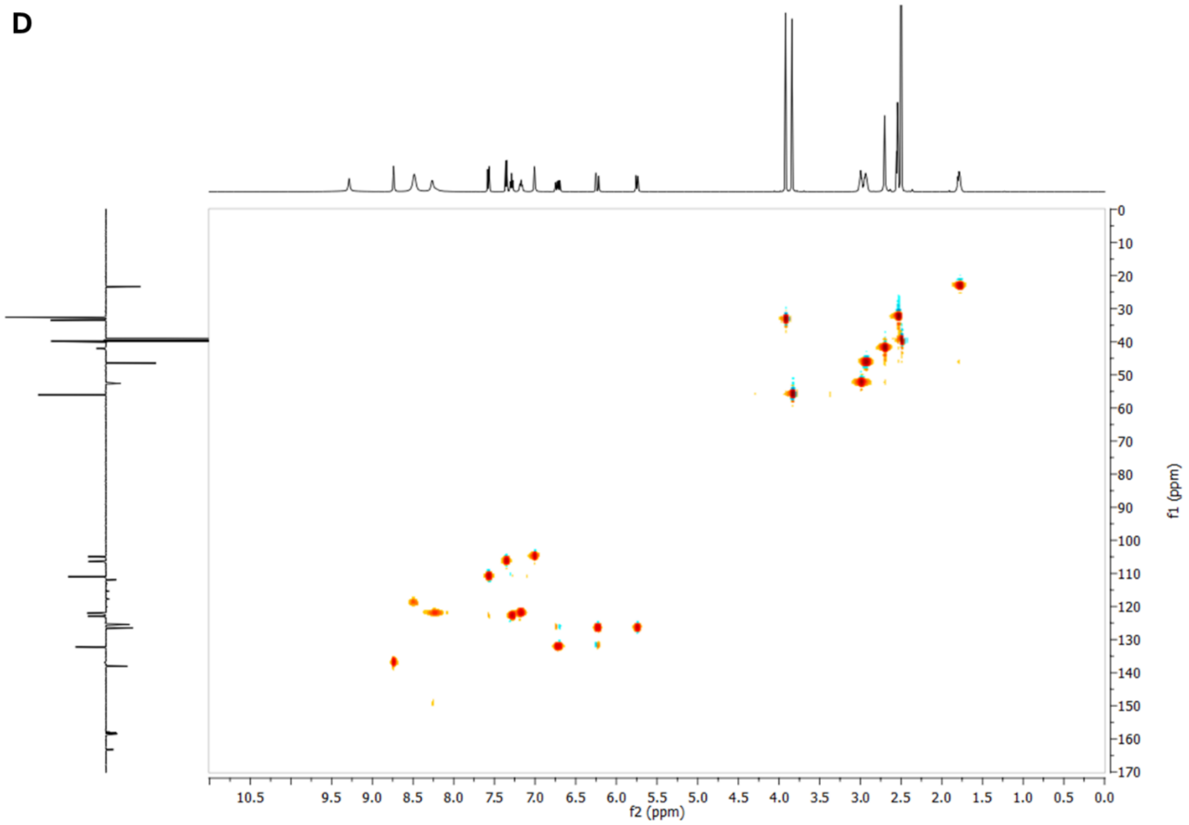

Figure S23 (continued): **C**) COSY NMR spectrum and **D**) HSQC NMR spectrum of **OsiPropNHMe**.

E

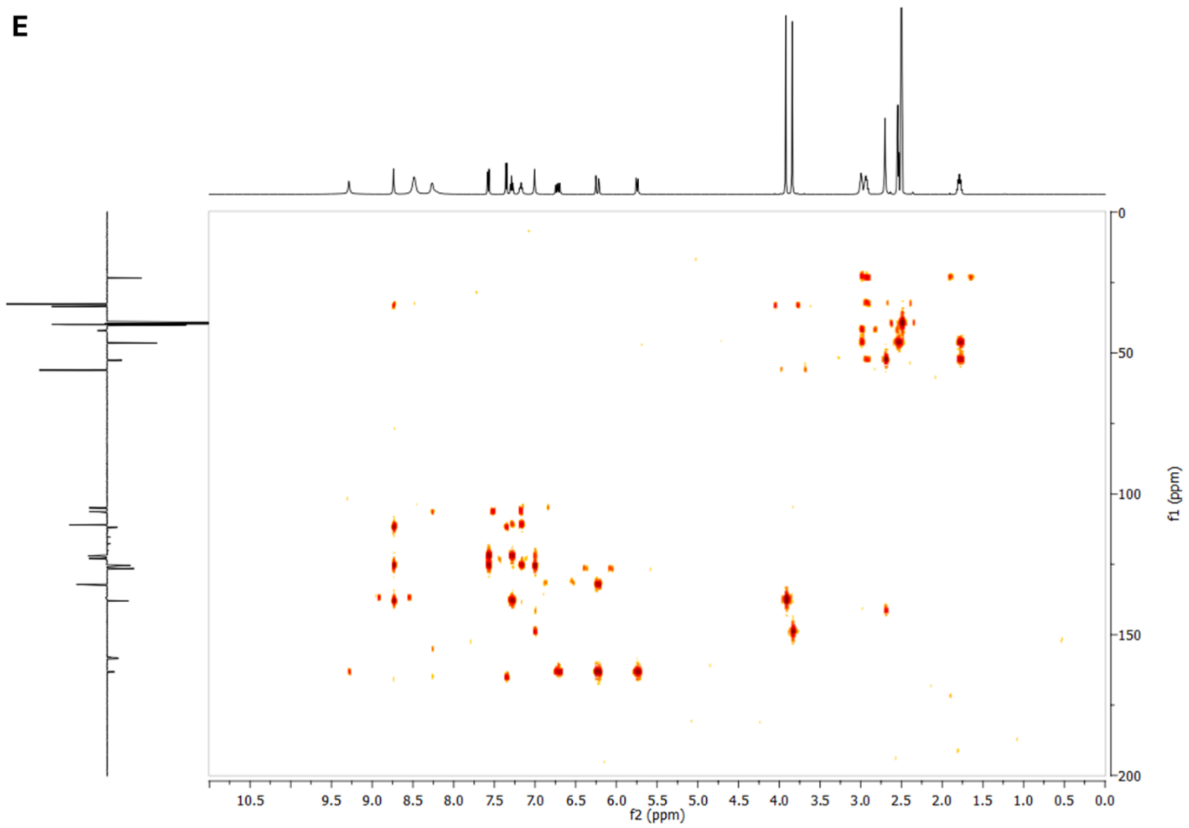

Figure S23 (continued): **E**) HMBC NMR spectrum of **OsiPropNHMe**.

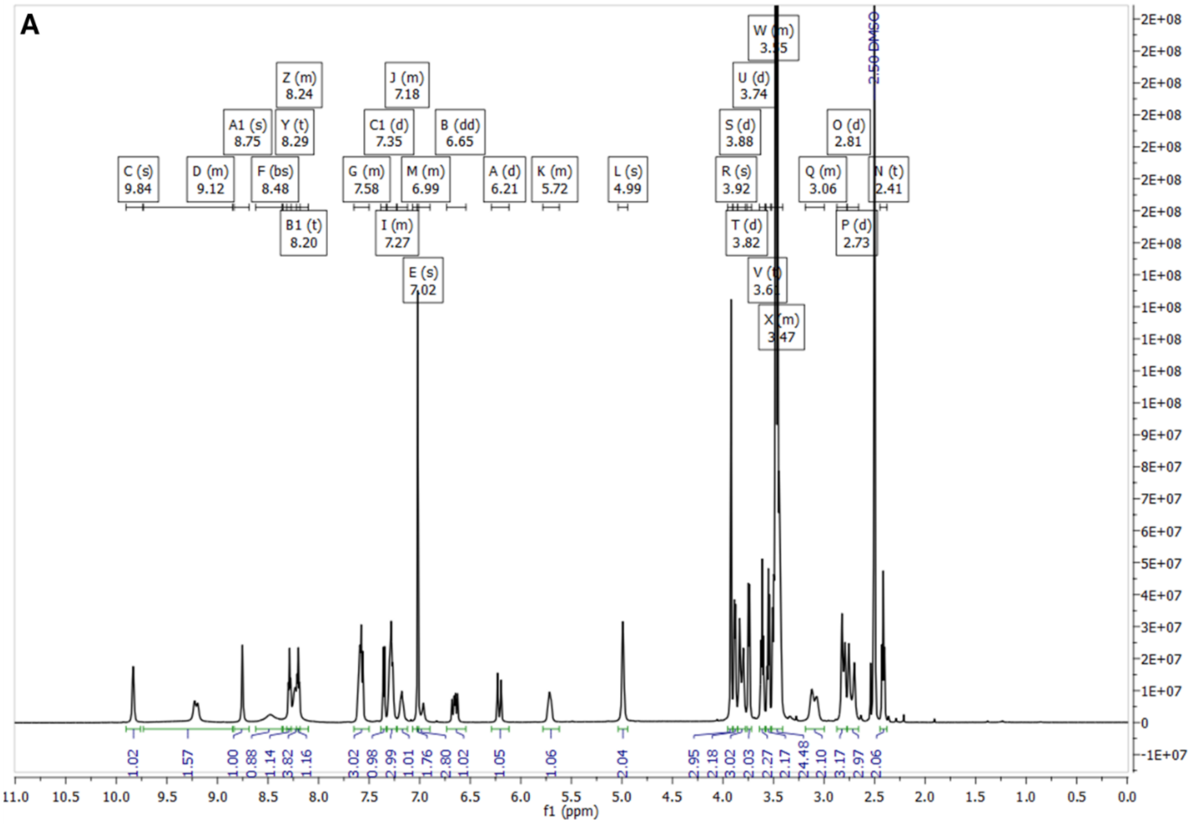

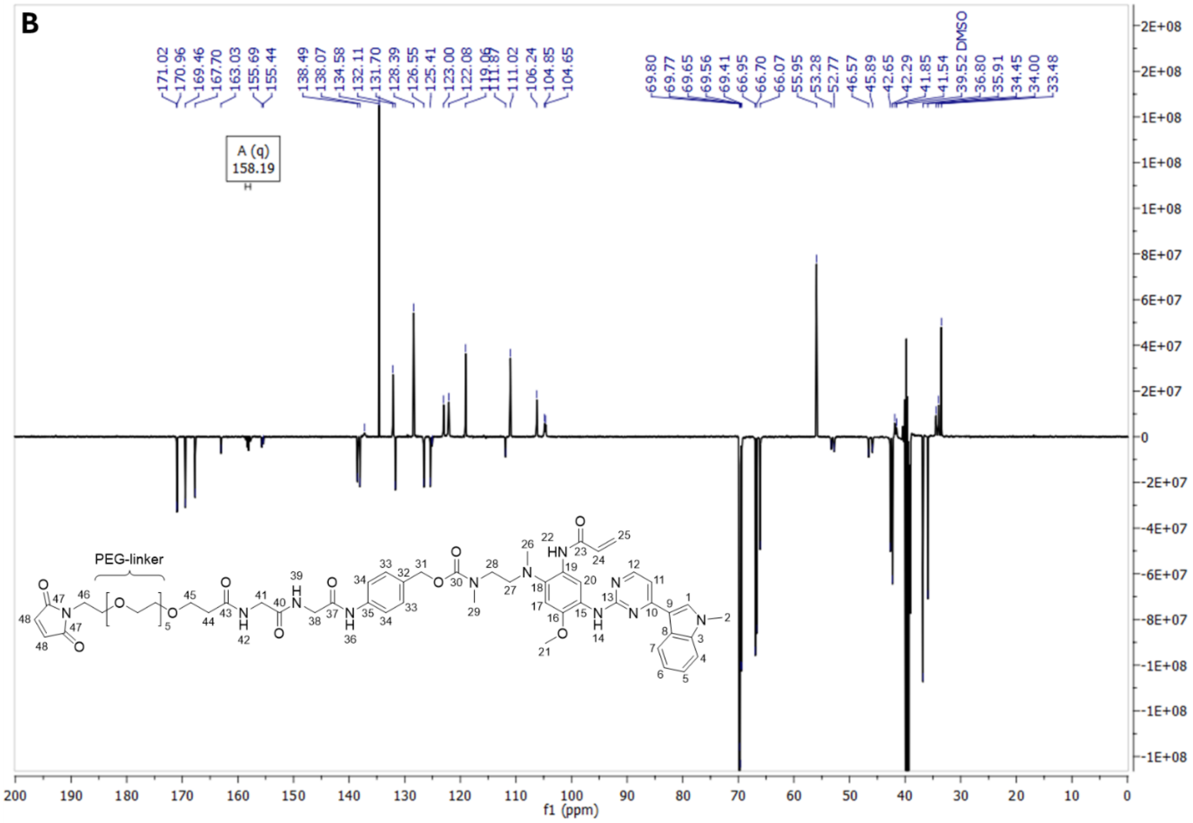

Figure S24: **A**) ^{1}H NMR spectrum, **B**) ^{13}C NMR spectrum and structure of **Mal-PEG-GlyGly**.

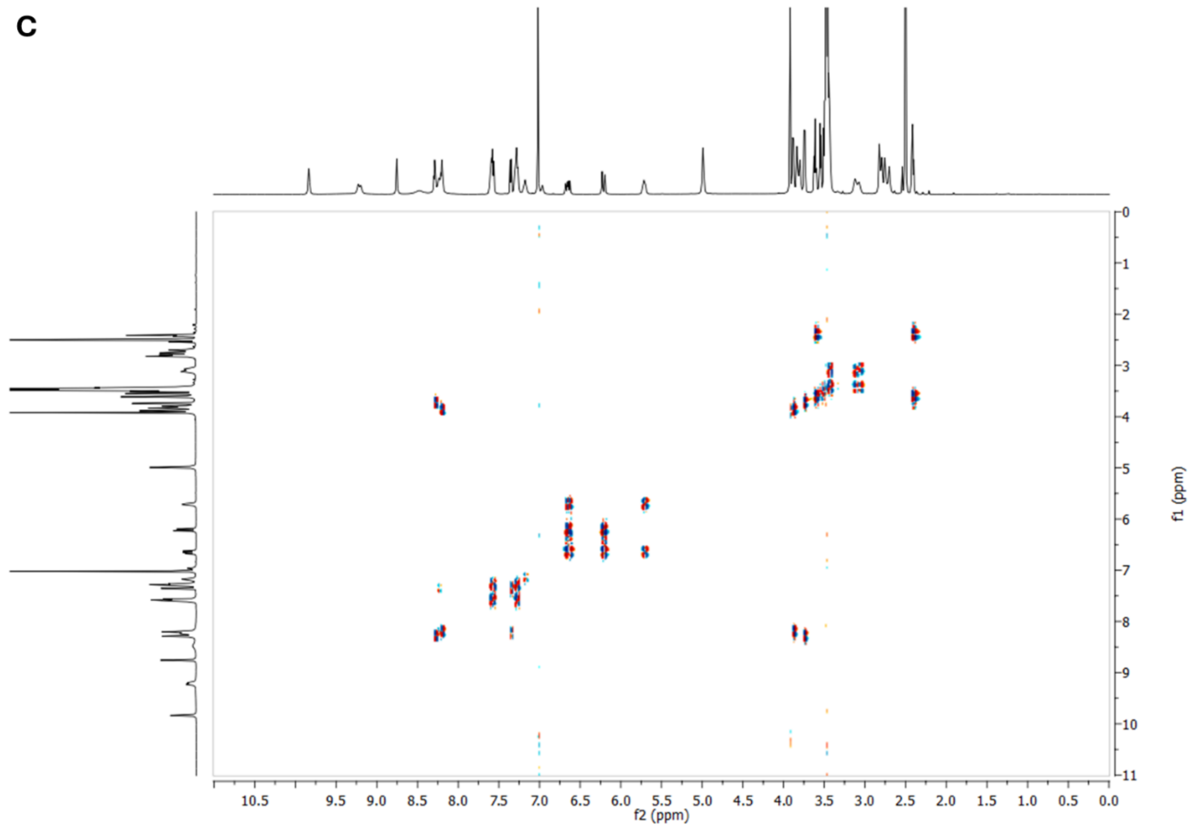

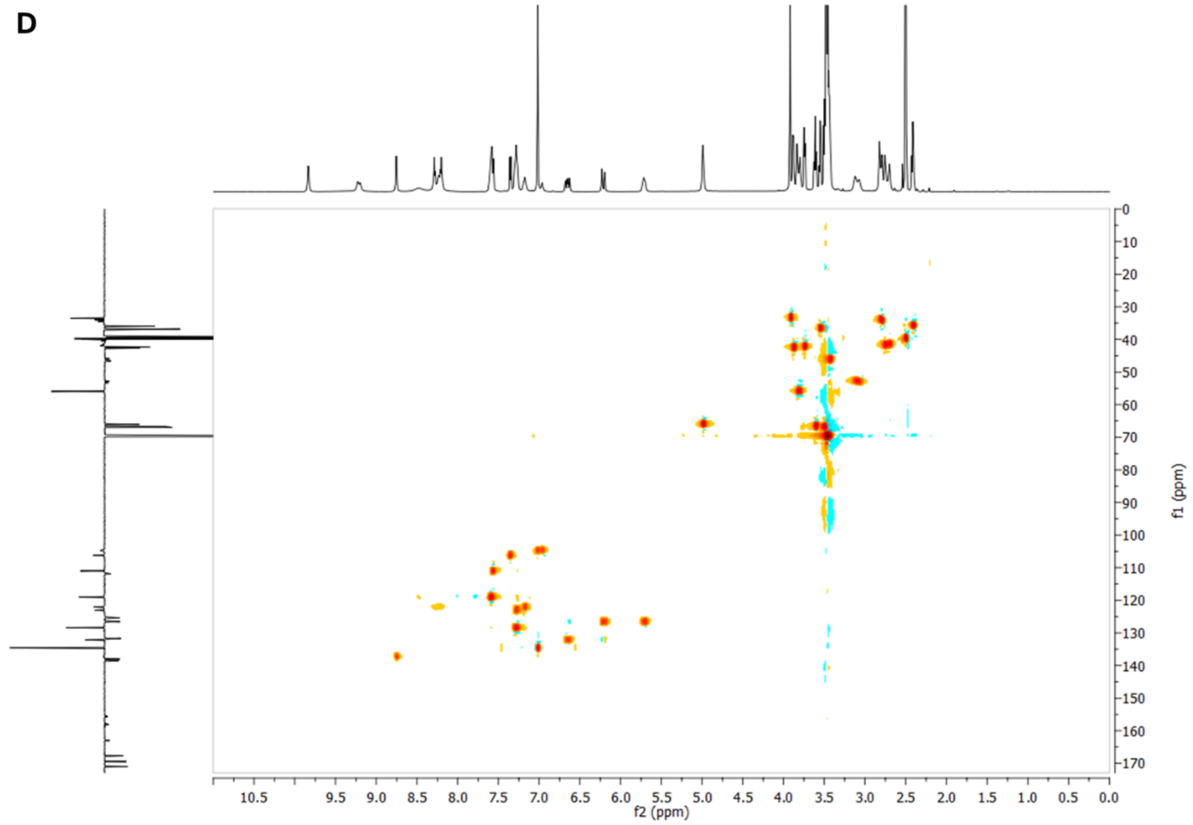

Figure S24 (continued): **C**) COSY NMR spectrum and **D**) HSQC NMR spectrum of **Mal-PEG-GlyGly**.

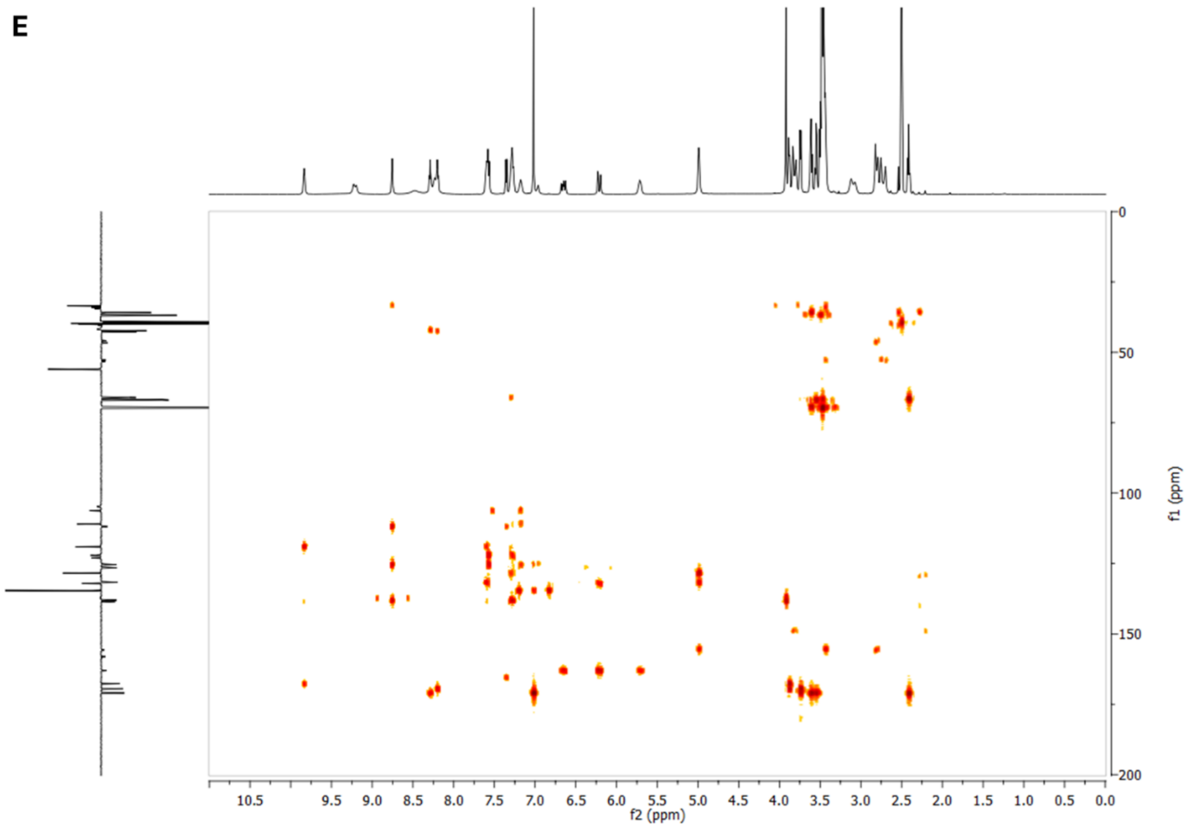

Figure S24 (continued): **E**) HMBC NMR spectrum of **Mal-PEG-GlyGly**.

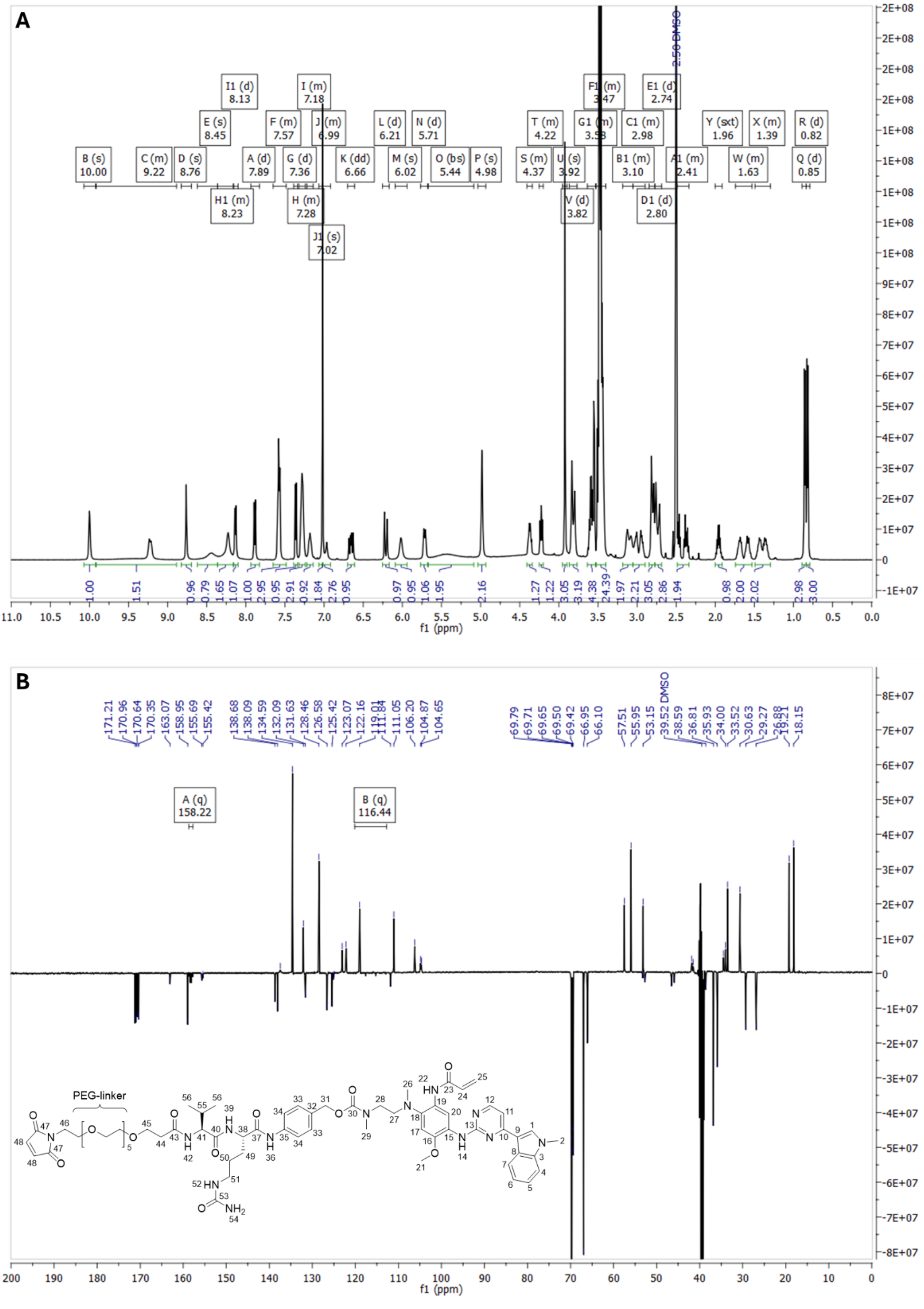

Figure S25: **A**) ^{1}H NMR spectrum, **B**) ^{13}C NMR spectrum and structure of **Mal-PEG-ValCit**.

C

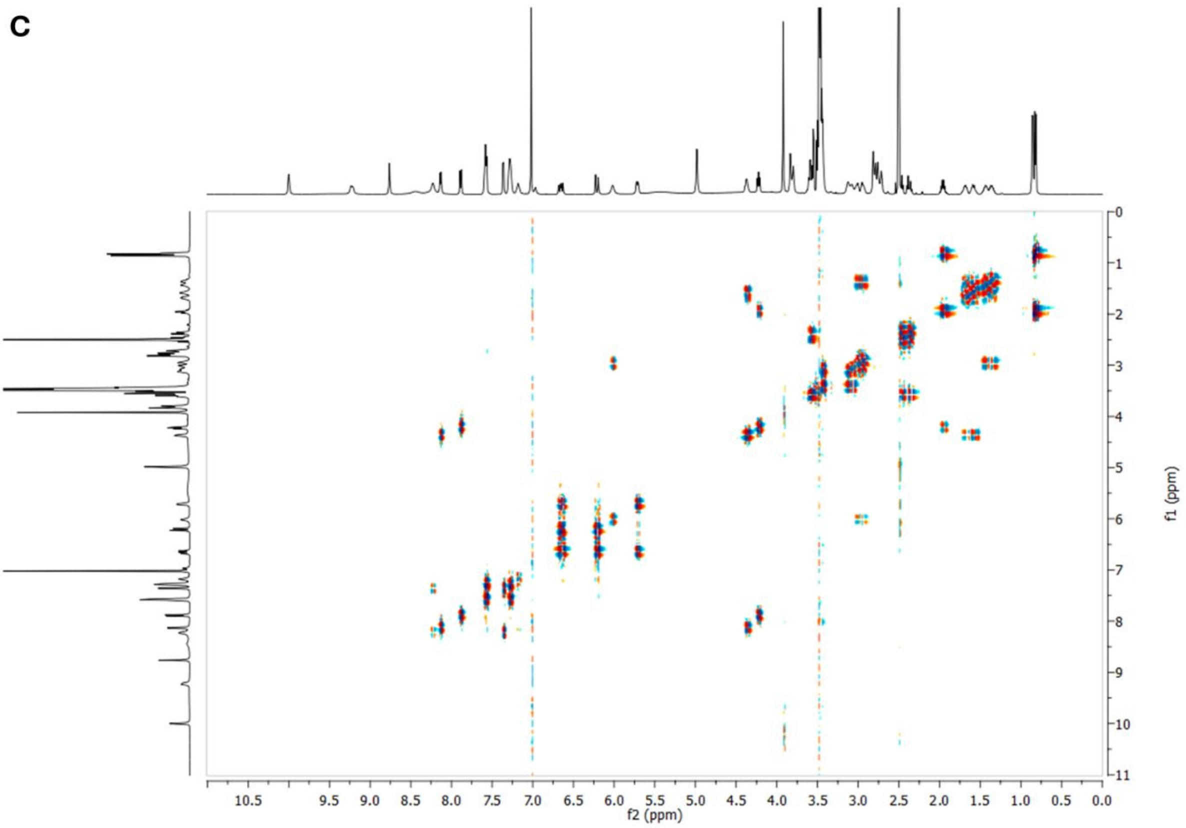

D

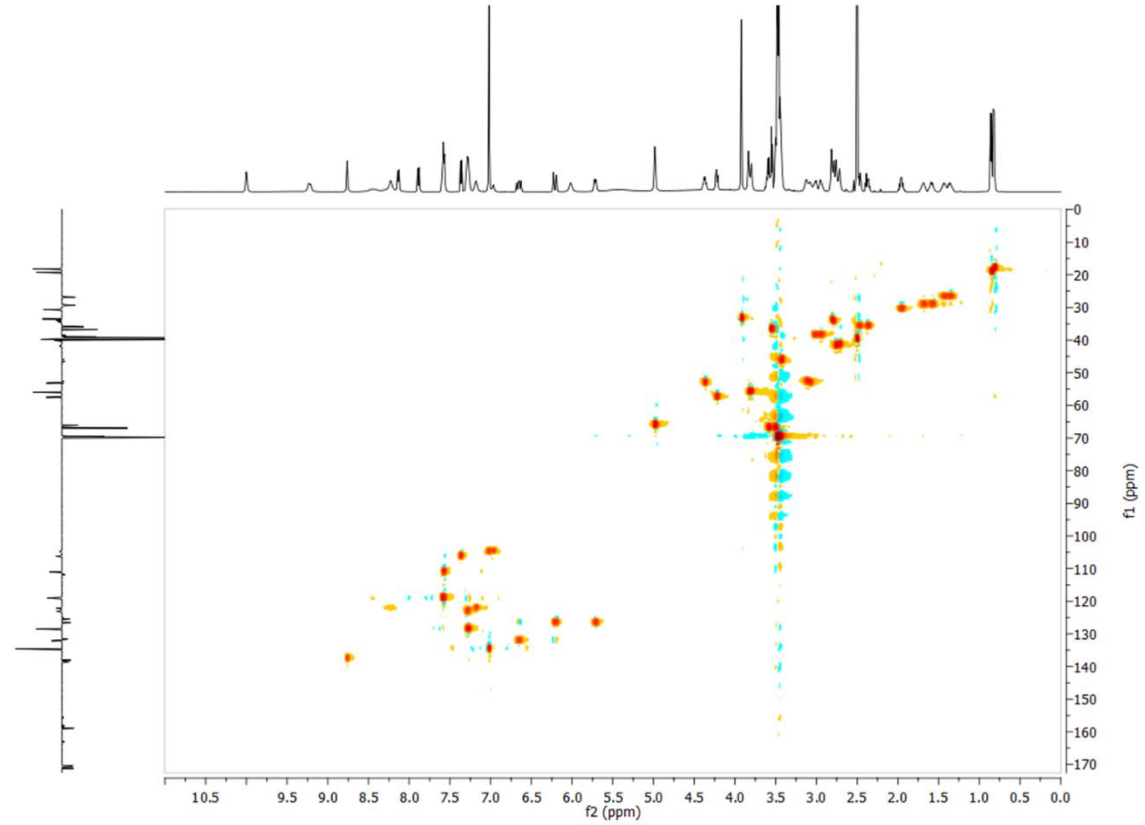

Figure S25 (continued): **C**) COSY NMR spectrum and **D**) HSQC NMR spectrum of **Mal-PEG-ValCit**.

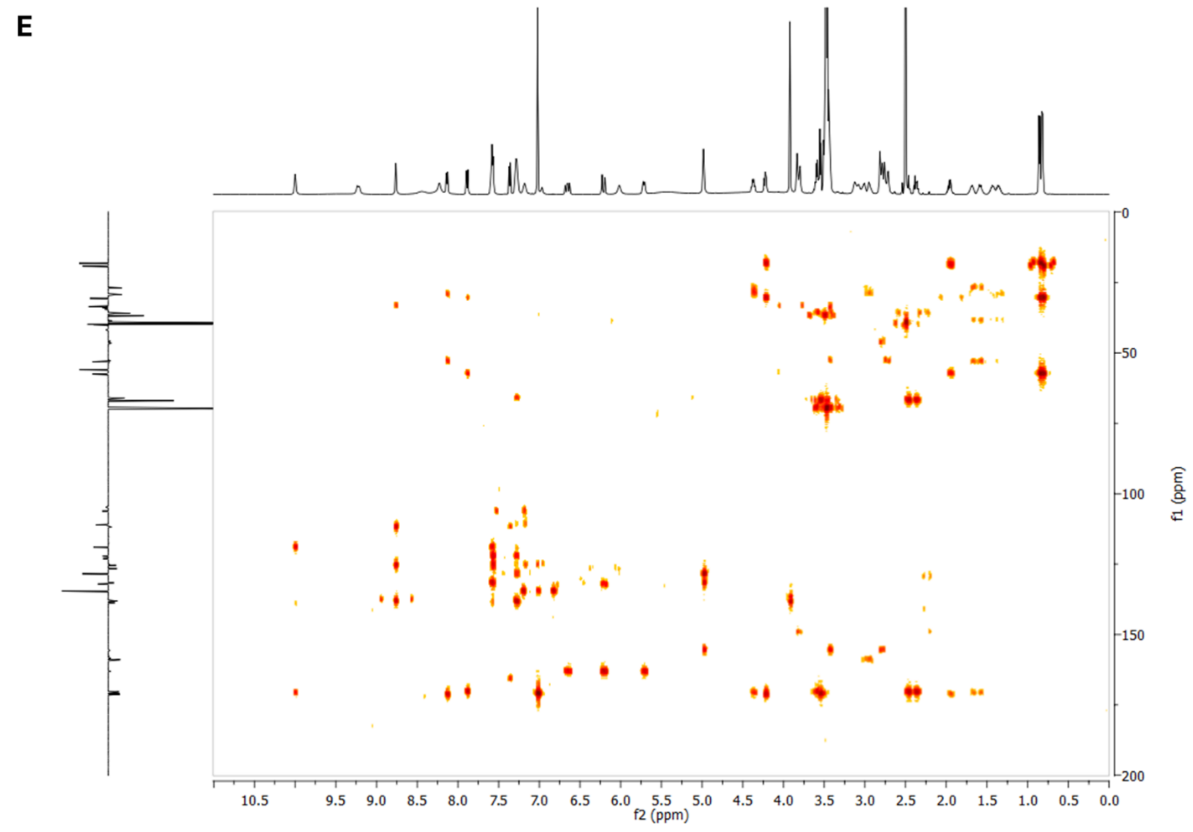

Figure S25 (continued): **E**) HMBC NMR spectrum of **Mal-PEG-ValCit**.

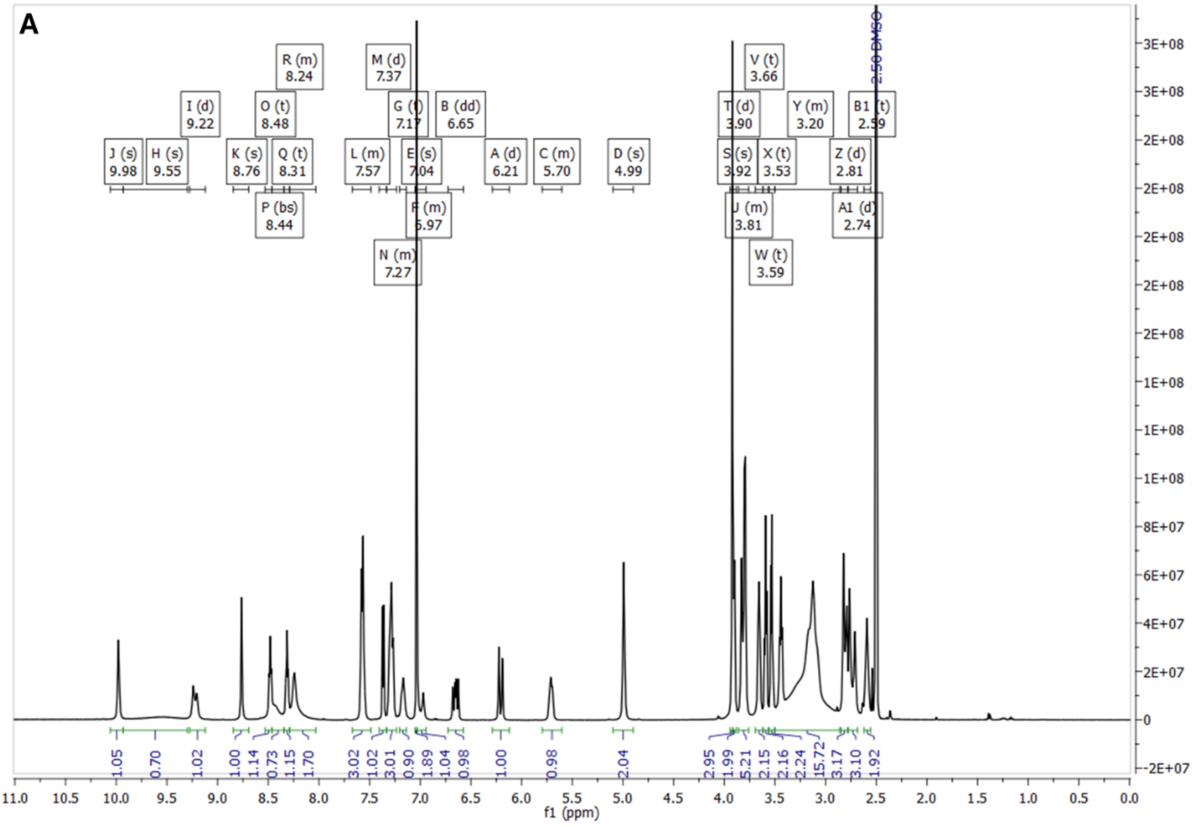

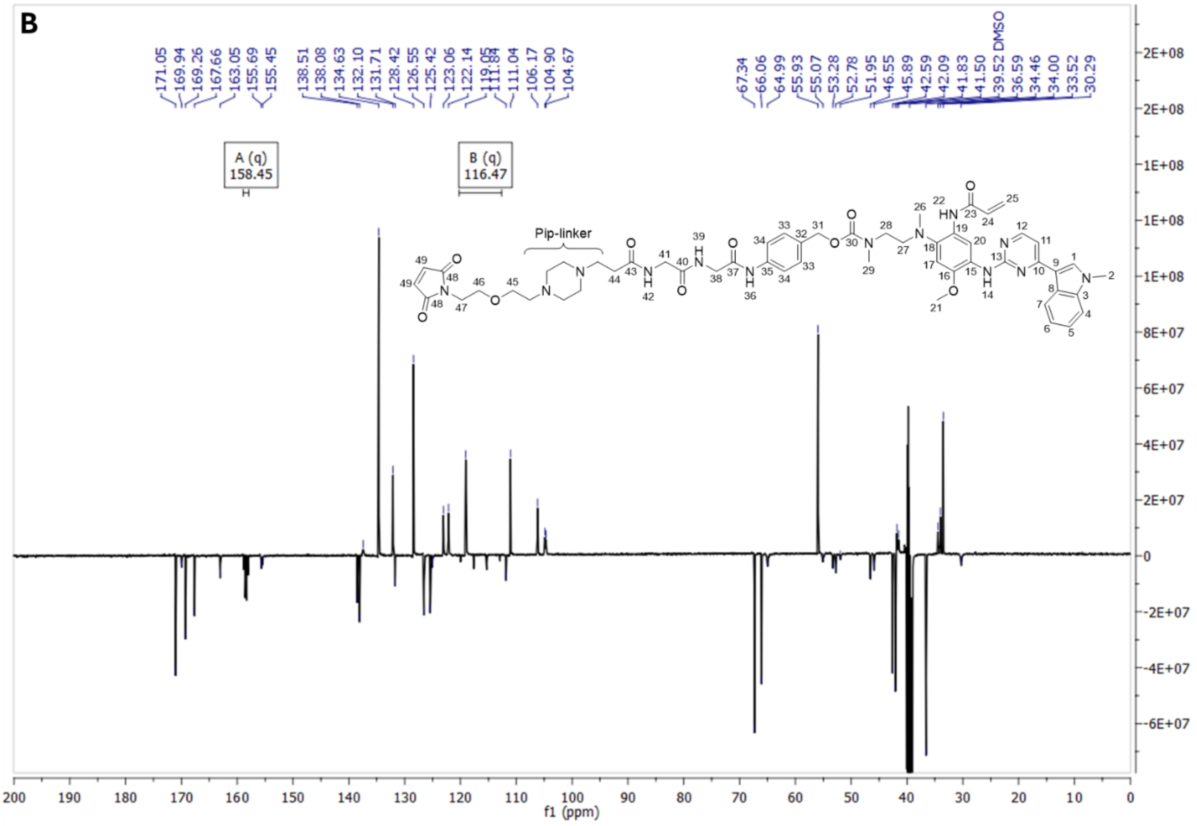

Figure S26: **A**) ^{1}H NMR spectrum, **B**) ^{13}C NMR spectrum and structure of **Mal-Pip-GlyGly**.

C

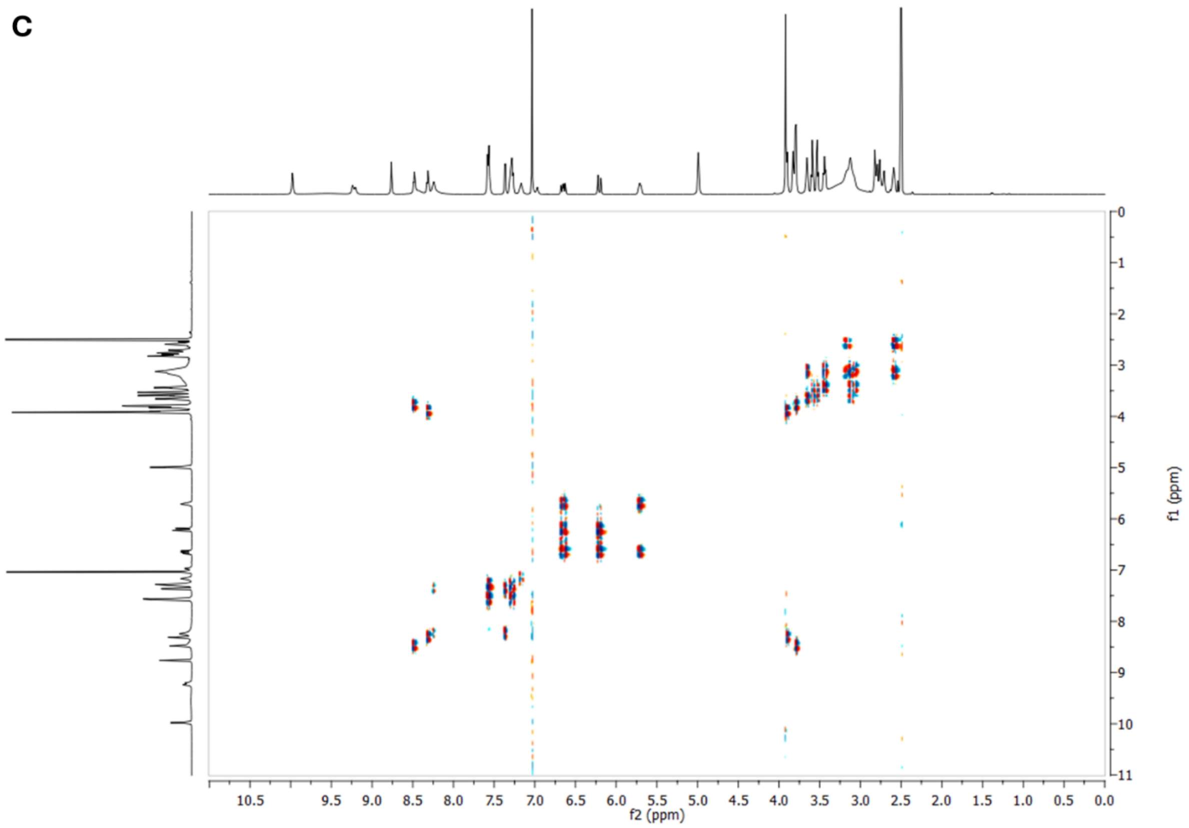

D

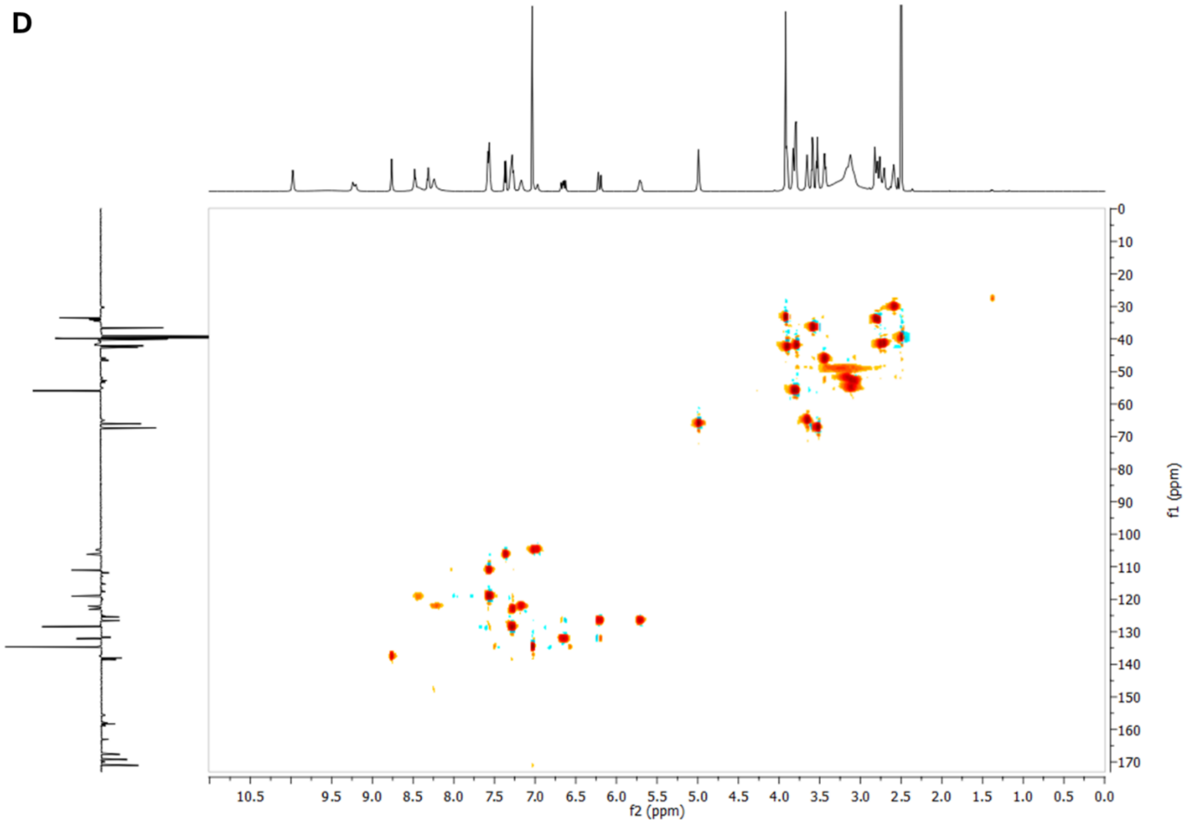

Figure S26 (continued): **C**) COSY NMR spectrum and **D**) HSQC NMR spectrum of **Mal-Pip-GlyGly**.

E

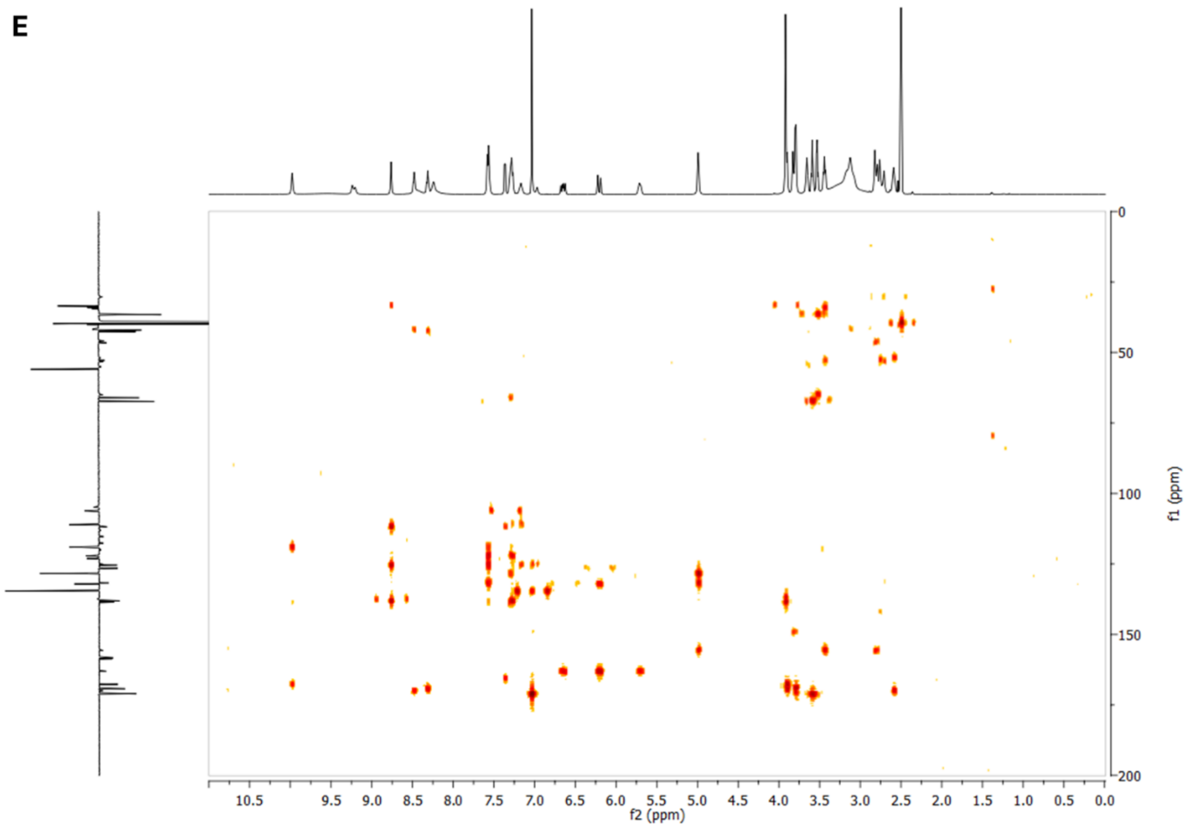

Figure S26 (continued): **E**) HMBC NMR spectrum of **Mal-Pip-GlyGly**.

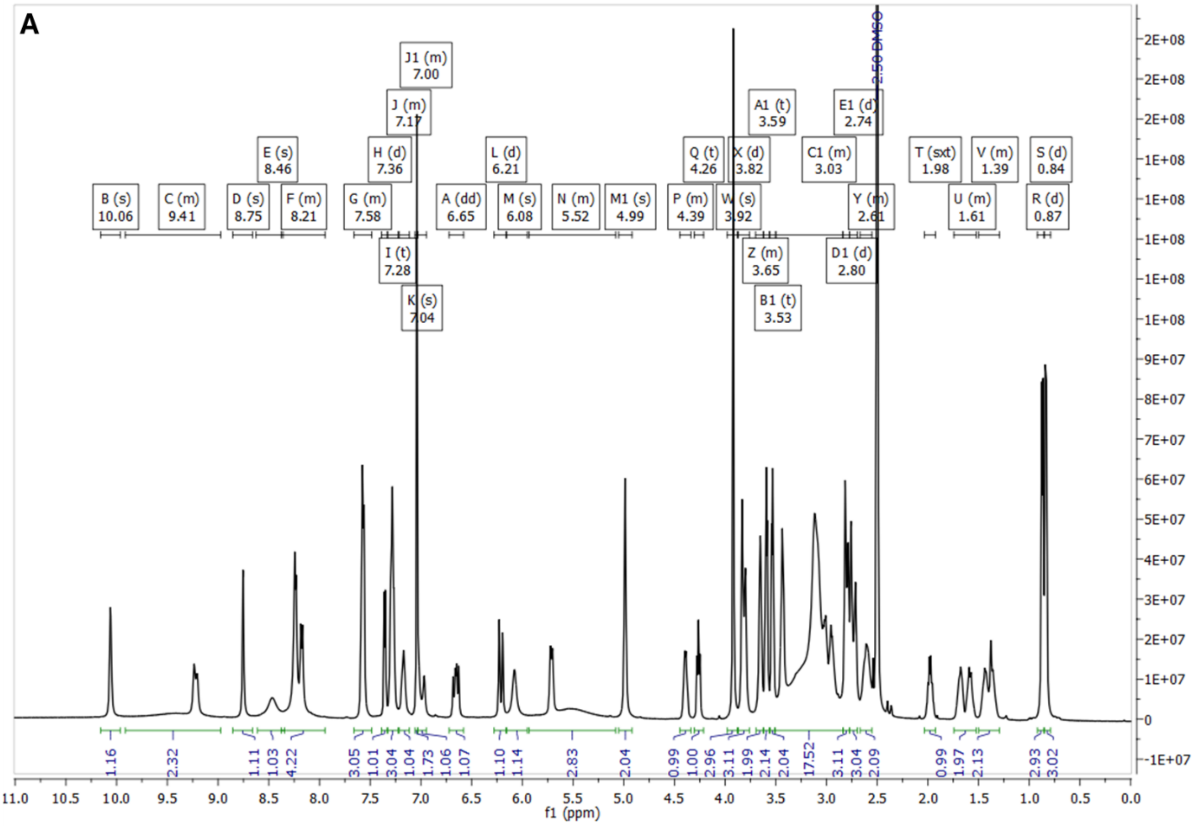

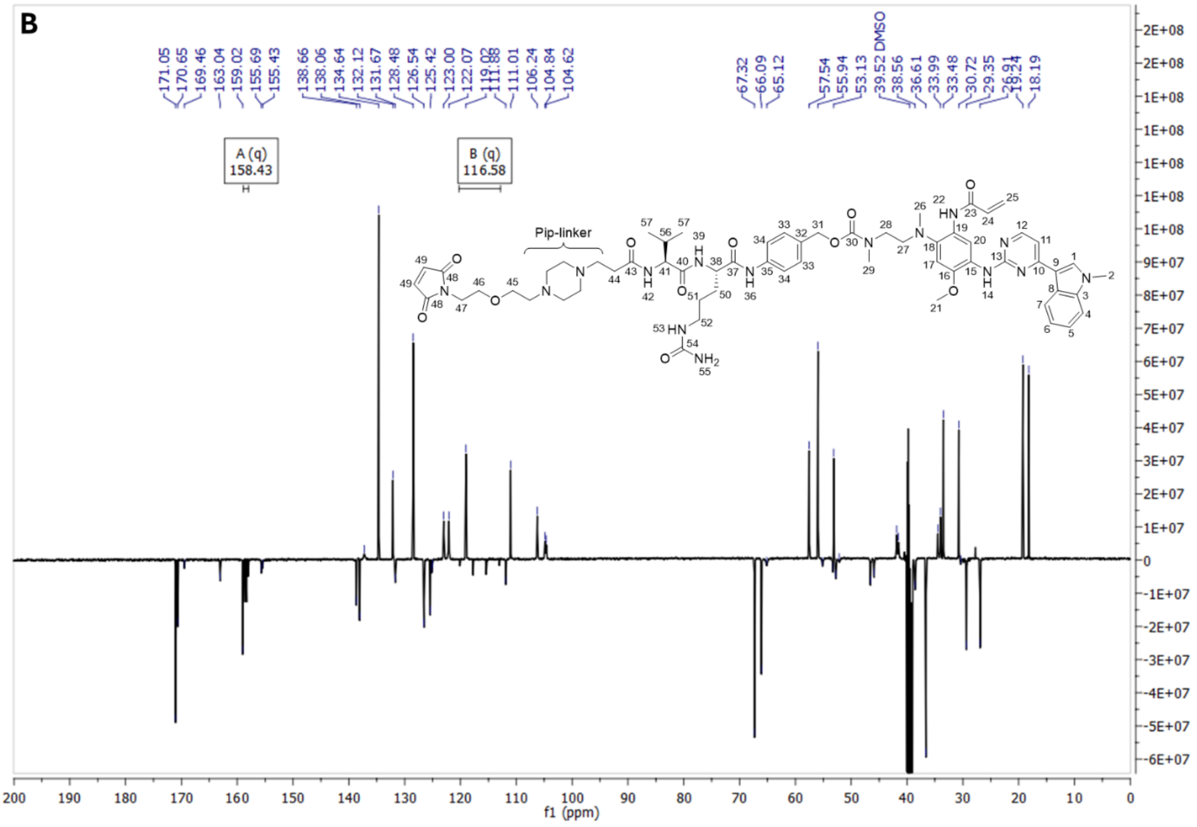

Figure S27: **A**) ^{1}H NMR spectrum, **B**) ^{13}C NMR spectrum and structure of **Mal-Pip-ValCit**.

C

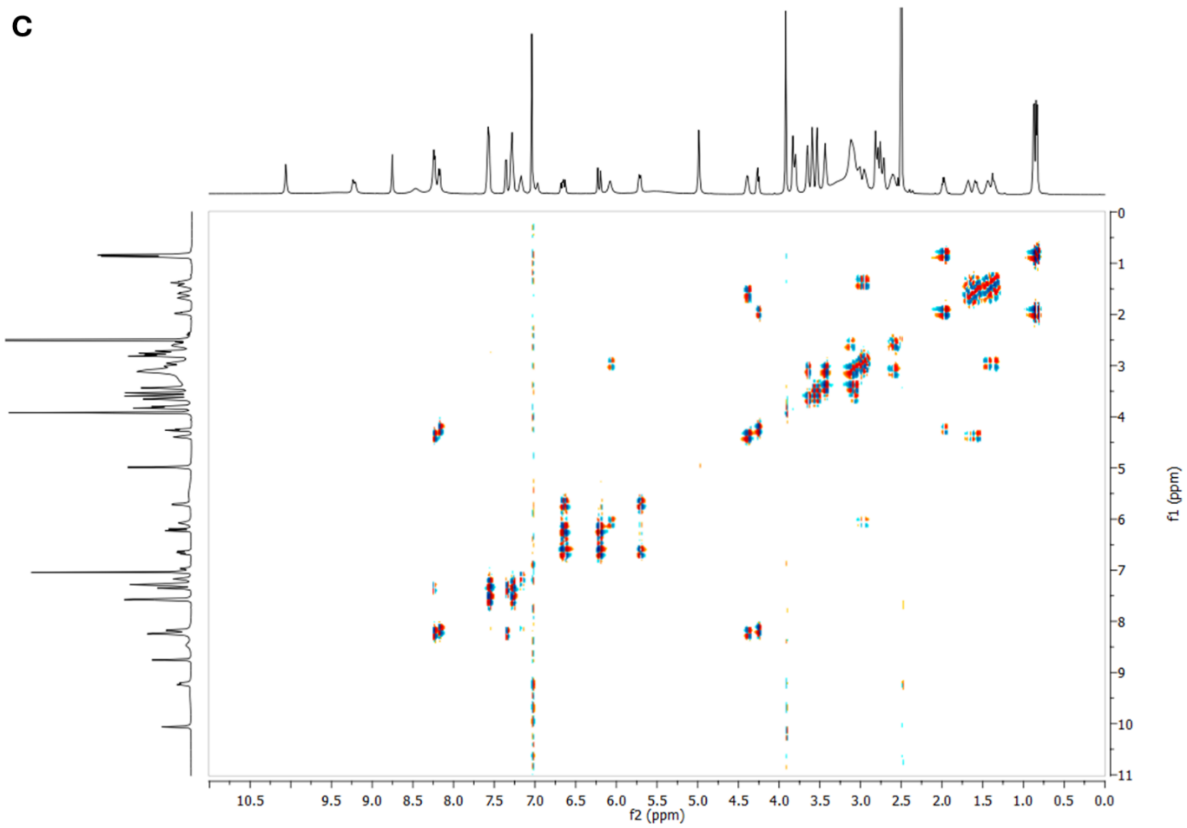

D

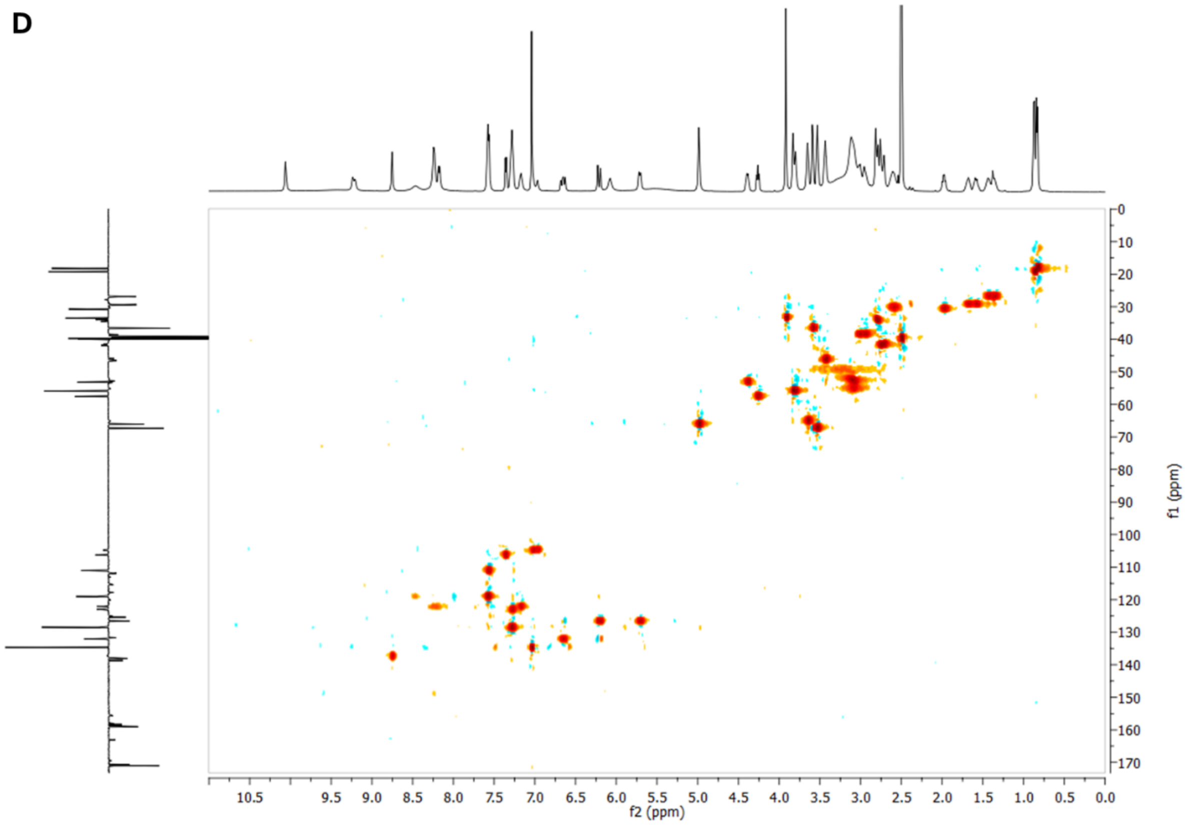

Figure S27: **C**) COSY NMR spectrum and **D**) HSQC NMR spectrum of **Mal-Pip-ValCit**.

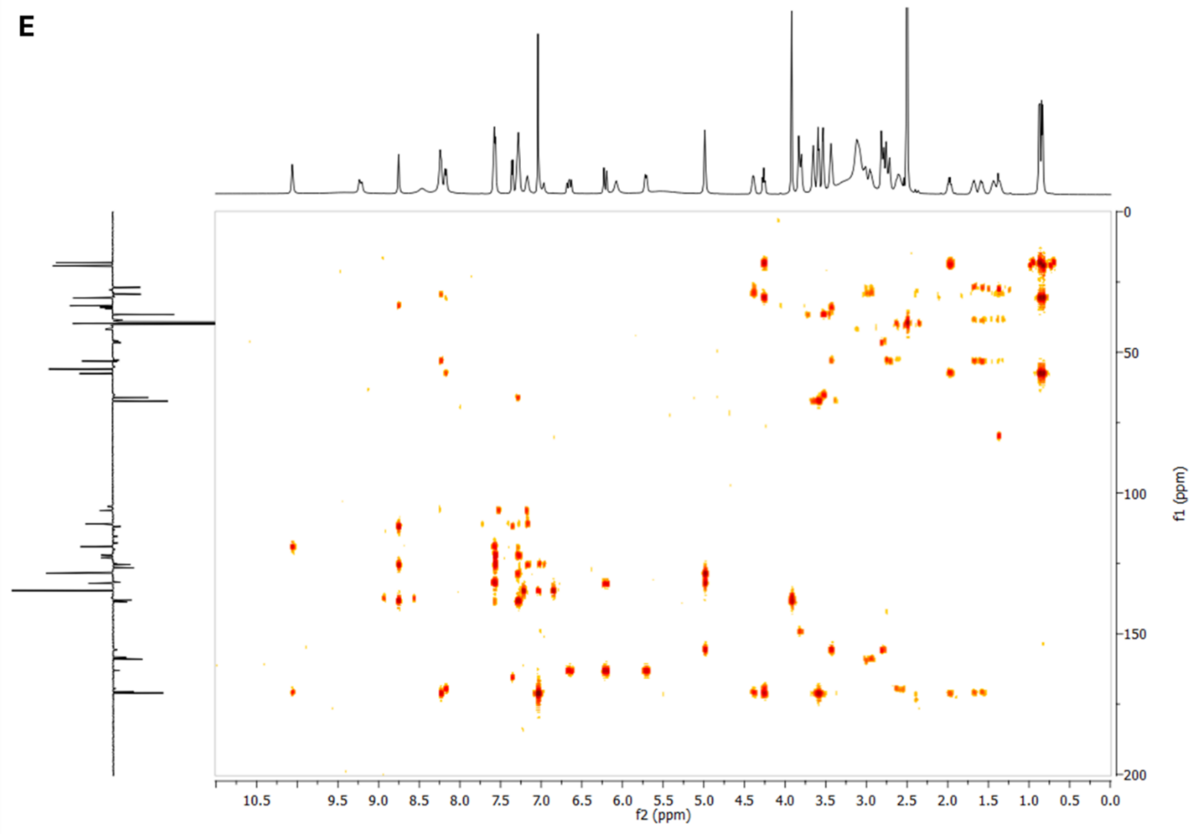

Figure S27: **E**) HMBC NMR spectrum of **Mal-Pip-ValCit**.

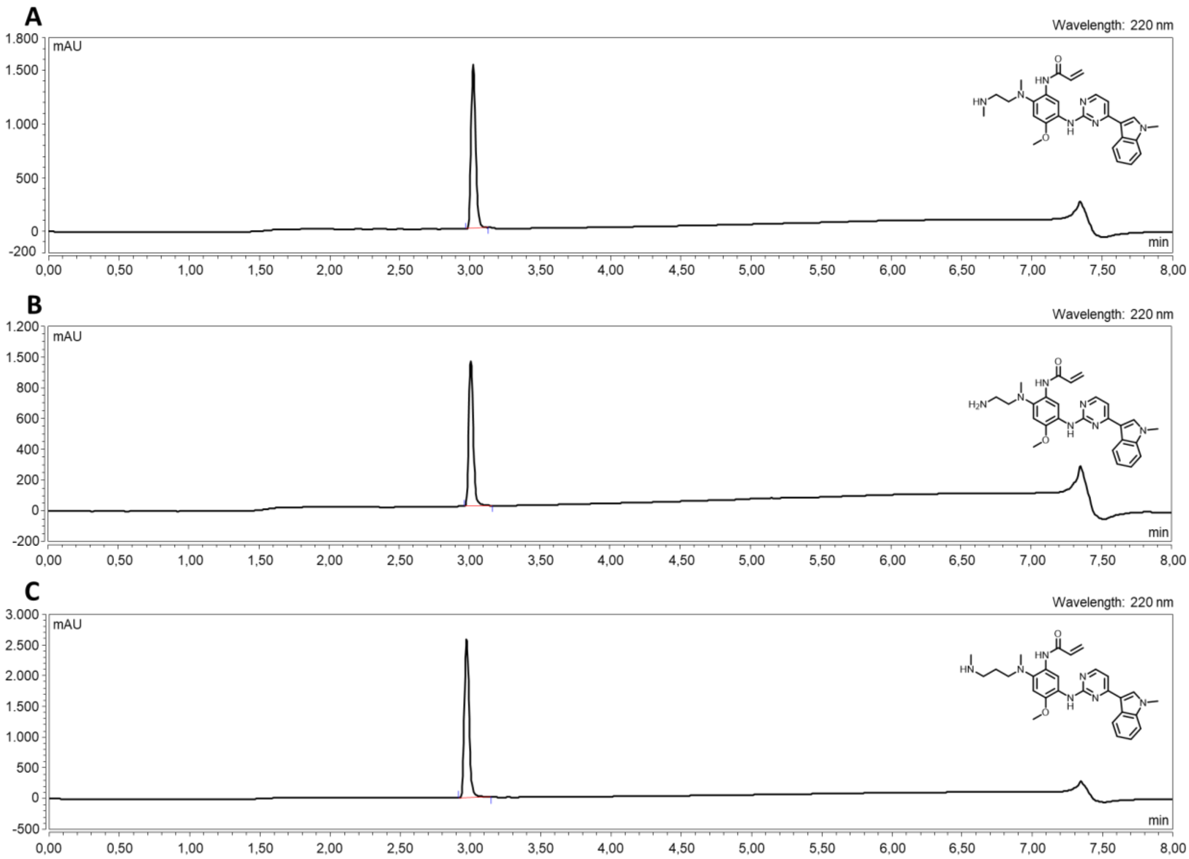

Figure S28: Quality control after purification with preparative HPLC. The compound retention times were **A**) 3.02 min for **OsiNHMe**, **B**) 3.01 min for **OsiNH₂** and **C**) 2.97 min for **OsiPropNHMe**. The solid samples were dissolved in a mixture of 5% v/v MeCN and 95% v/v Milli-Q water containing 0.1% TFA and measured with HPLC on the same instrument used for stability and cathepsin B cleavage measurements (gradient: 0–0.5 min A 95:5 B, 0.5–6-0 min linear gradient to A 5:95 B, 6.0–7.0 min A 5:95 B, 7.0–7.1 min linear gradient to A 95:5 B). For all compounds only one peak was observed, confirming purity.

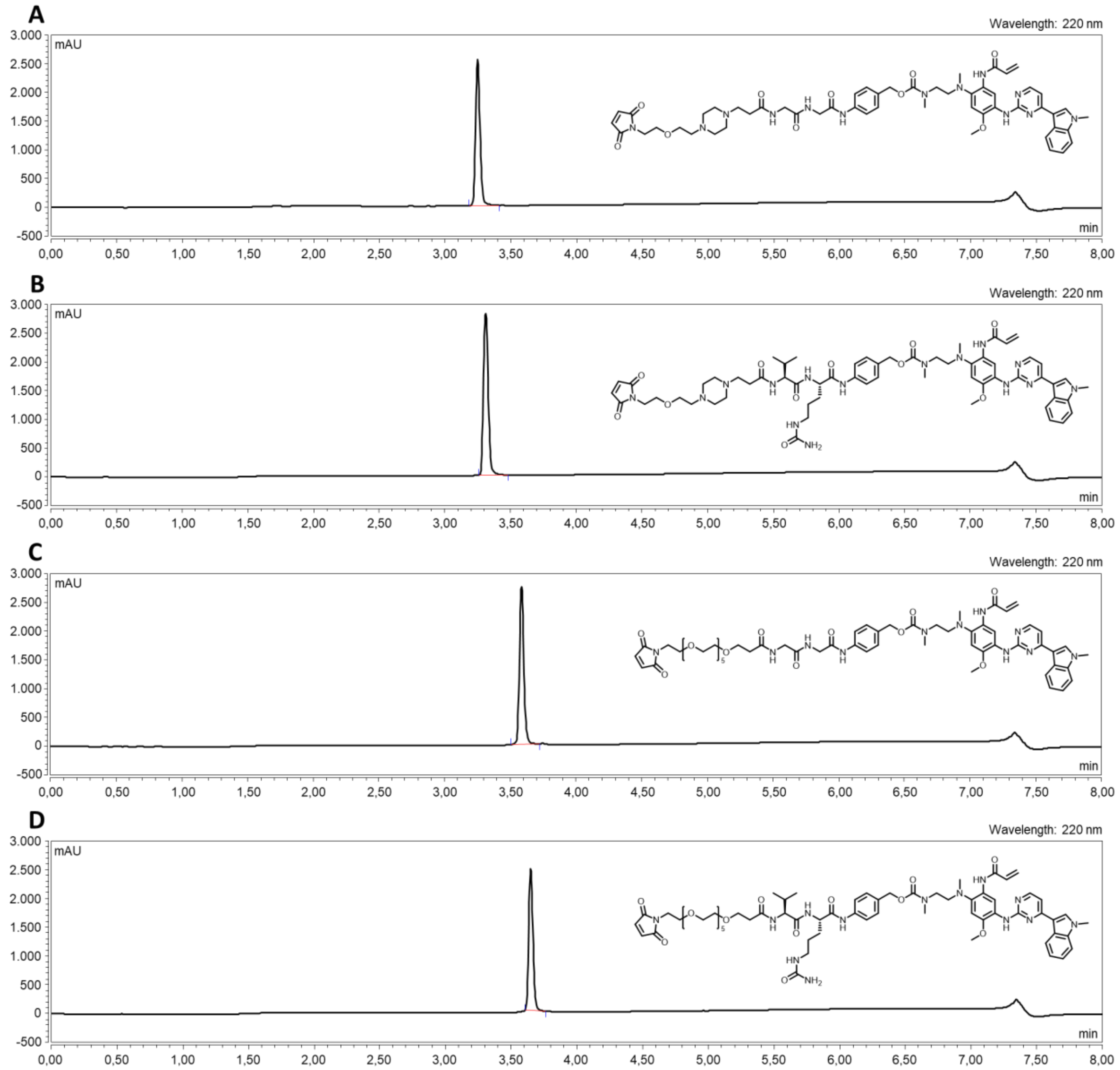

Figure S29: Quality control after purification with preparative HPLC. The compound retention times were **A**) 3.25 min for **Mal-Pip-GlyGly**, **B**) 3.31 min for **Mal-Pip-ValCit**, **C**) 3.51 min for **Mal-PEG-GlyGly** and **D**) 3.65 min for **Mal-PEG-ValCit**. The solid samples were dissolved in a mixture of 5% v/v MeCN and 95% v/v Milli-Q water containing 0.1% TFA and measured with HPLC on the same instrument used for stability and cathepsin B cleavage measurements (gradient: 0–0.5 min A 95:5 B, 0.5–6-0 min linear gradient to A 5:95 B, 6.0–7.0 min A 5:95 B, 7.0–7.1 min linear gradient to A 95:5 B). For all compounds only one peak was observed, confirming purity.
